# How do Nanohoops Exercise Their Strain in [5]Helicene Racemization?

**DOI:** 10.1021/acs.joc.5c02280

**Published:** 2025-11-10

**Authors:** Kovida Kovida, Juraj Malinčík, Thijs de Groot, Tomáš Šolomek

**Affiliations:** Van’t Hoff Institute for Molecular Sciences (HIMS), 1234 University of Amsterdam, P.O. Box 94157, 1090 GD Amsterdam, The Netherlands

## Abstract

The effect of strain in curved carbon nanohoops has a major effect on their optoelectronic properties. How this strain affects their dynamics and reactivity is, however, far less documented in the literature. Here, we investigate the effect of strain on racemization of [5]­helicene, which we embedded in three different [*m*]­cycloparaphenylenes (*m* = 5–7). [5]­Helicene is a prototypical organic molecule with helical chirality that racemizes at room temperature. We synthesized and separated the enantiomers of the strained helicene-paraphenylene macrocycles, and we investigated their racemization as a function of temperature. Our results revealed that the configurational stability of the [5]­helicene increases with increasing nanohoop size, with activation free energies ranging from 25 to 29 kcal mol^–1^. The combination of the experimental data and DFT calculations established a clear relationship between the strain and racemization in helicene-embedded nanohoops.

## Graphical abstract

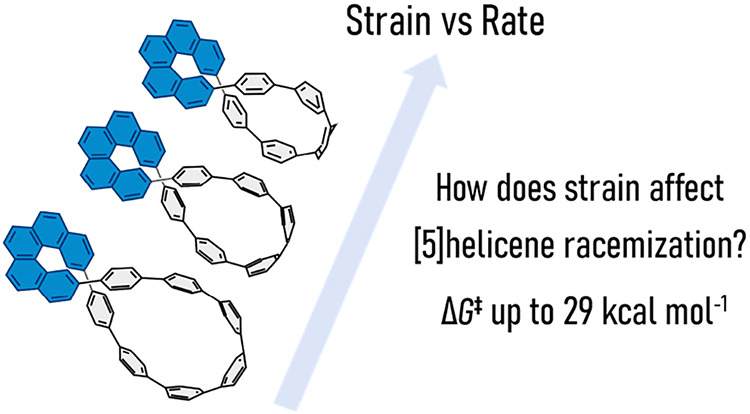

## Introduction

Helicenes, a class of chiral polycyclic aromatic hydrocarbons with *ortho*-fused phenylenes adopting helical shape, are of great interest in asymmetric catalysis, molecular electronics, and chiral sensing.^1–3^ A critical stereodynamic characteristic of [*n*]­helicenes is their configurational stability, which directly impacts any chirality-related application of helicenes. Experimental and theoretical studies^4–10^ have shown that the racemization mechanism in helicenes varies with the size of the helix. Shorter helicenes (*n* = 4–7) undergo a concerted, single-step inversion, while larger systems (*n* ≥ 8) follow a multistep pathway. The shortest of the helicenes, [4]­helicene, is configurationally unstable, but [5]­helicene exhibits an activation free energy (Δ*G*_T_^‡^, *T* = 298 K) of 24.1 kcal mol^–1^,^11^ sufficient for resolution of the enantiomers. However, racemization occurs in a matter of days. From [6]­helicene onward, the enantiomers remain configurationally stable at room temperature.^9^

To improve the configurational stability of [5]­helicene, substituents can be installed to its fjord, noncovalently “locking” the configuration by employing steric hindrance (Figure 1). Placement of a single methoxy group improves the Δ*G*_423_^‡^ to 32 kcal mol^–1^, and increasing the steric bulk with methyl substituents in the 1,14 positions accomplishes the [5]­helicene derivative with the configurational stability that matches [9]­helicene with Δ*G*_503_^‡^ ∼44 kcal mol^–1^.^8^ The substituents in the fjord increase the C_1_–C_14_ distance (*d*_1–14_) and helical pitch. The latter is expressed by a twist angle, θ, which is calculated as an average value of torsional angles of all phenanthrene subunits in [5]­helicene. Alternative approaches to configurational stabilization include structural rigidification through covalent locking of multiple [5]­helicenes within a macrocycle. Cyclobis[5]­helicenes, for instance, interconnect helicene units in a *D*_2_ symmetry structure, resulting in elevated racemization barriers (Figure 1).^12,13^ Ultimate stabilization is achieved using topological locking, with fused [5]­helicene dimers^14^ and structures like infinitene or triply twisted Möbius carbon nanobelt,^15,16^ which cannot racemize. These locking strategies have enabled the development of persistent, enantiopure helicene-based materials with tunable chiroptical properties.

**Figure 1:**
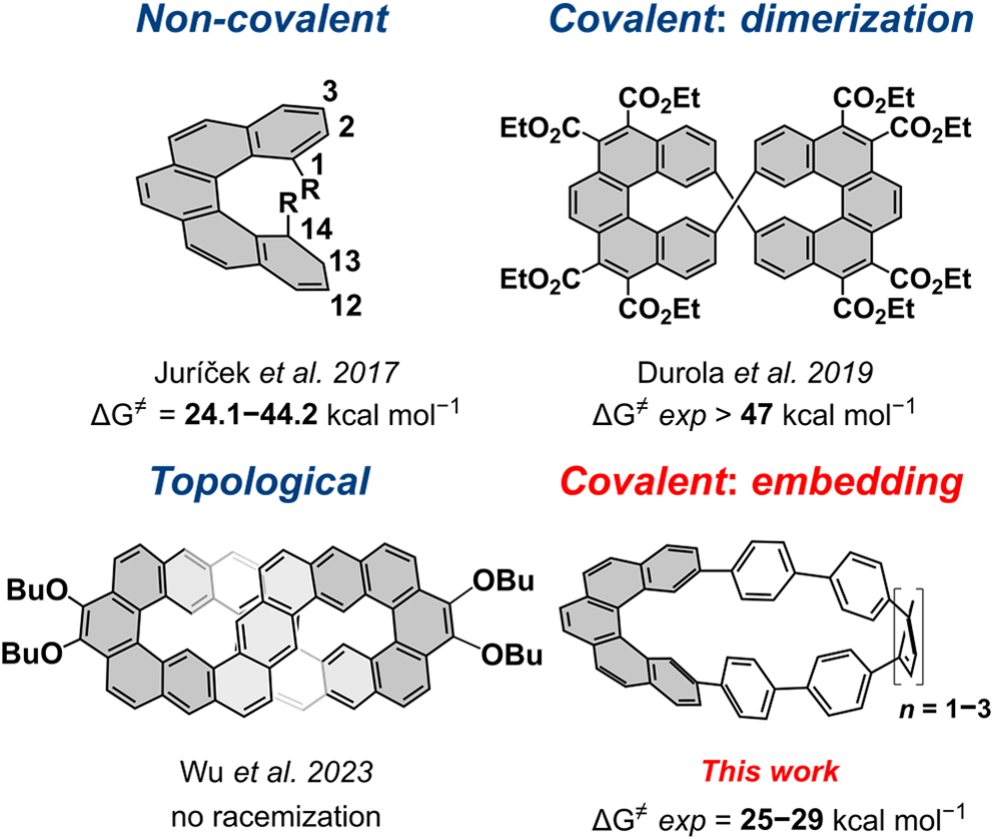
Configurational “locking” in [5]­helicenes.

We previously reported the first [6]­helicene-based chiral nanohoop, in which a single [6]­helicene is formally embedded in highly strained [7]­cycloparaphenylene ([7]­CPP).^17^ The resulting helicene-phenylene (**[6,7]­HPP**) displayed an unusual topological feature, namely, nonorientability of its π-electron system.^18^ The compound exhibited remarkable configurational stability and strong, bright circularly polarized luminescence (CPL). Recently, we extended this design by embedding [5]­helicene and benzothiadiazole (BTD) to [6]­CPP creating^19^ a bright CPL luminophore with λ_*em*_ > 650 nm to test how excited state delocalization affects chiroptical properties of chiral nanohoops.^20^ Despite lacking fjord substitution, [5]­helicene remained configurationally stable, showing no noticeable racemization over the course of extensive CPL measurements.

This led us to explore how strain in **[5**, ***m*** **]­HPPs** (Figure 1), where *m* denotes the size of the embedding CPP, influences [5]­helicene racemization. Strain in carbon nanohoops has been harnessed to enhance their reactivity, for instance, in click chemistry of alkyne-containing [*m*]­CPPs,^21^ their electrophilic bromination,^22^ or in anthracene-dimer cycloreversion.^23^ Strain also enabled efficient photouncaging of Fe^2+^ from a ferrocene nanohoop.^24^ However, a systematic experimental examination of strain in the racemization of a carbon nanohoop system is absent.^25,26^

## Results and Discussion

We opted for the synthesis (Scheme 1) of a series of three helicene carbon nanohoops, [5,*m*]­HPPs (*m* = 5–7), where *m* controls the overall strain in the macrocycle. The synthesis of the smallest **[5,5]­HPP** commences with Suzuki–Miyaura cross-coupling macrocyclization of 2,13-dibromo-[5]­helicene^27^ **1** with building block^28^ **2** containing two proaromatic cyclohexadienes. We isolated the proaromatic macrocyclic ***pro*** **-[5,5]­HPP** precursor in 24% yield after purification via recycling GPC (see SI). The ***pro*** **-[5,5]­HPP** was subjected to reductive aromatization using H_2_SnCl_4_ acid^29^ affording **[5,5]­HPP** in 80% isolated yield. The **[5,6]­HPP** was synthesized through a Suzuki–Miyaura cross-coupling of **1** and building block **3**,^30^ affording the intermediate **4a** in 80% yield. Dichloride **4a** was subsequently subjected to Miyaura borylation to yield **4b**, followed by its oxidative homocoupling under conditions developed by Jasti and co-workers,^31^ affording ***pro*** **-[5,6]­HPP** in 52% yield over the two steps. Final reduction of ***pro*** **-[5,6]­HPP** with H_2_SnCl_4_ furnished **[5,6]­HPP** in an excellent 97% yield. The synthesis of **[5,7]­HPP** commenced with a Suzuki–Miyaura cross-coupling of compounds **4a** and **5**, affording ***pro*** **-[5,7]­HPP**, followed by its aromatization with H_2_SnCl_4_, affording the desired nanohoop in 25% overall yield over two steps. All final compounds were characterized by ^1^H and ^13^C NMR spectroscopy and high-resolution mass spectrometry (see SI). We note that during writing this manuscript, Jiang reported a different synthesis of **[5,7]­HPP**,^32^ but not its stereodynamic behavior.

**Scheme 1:**
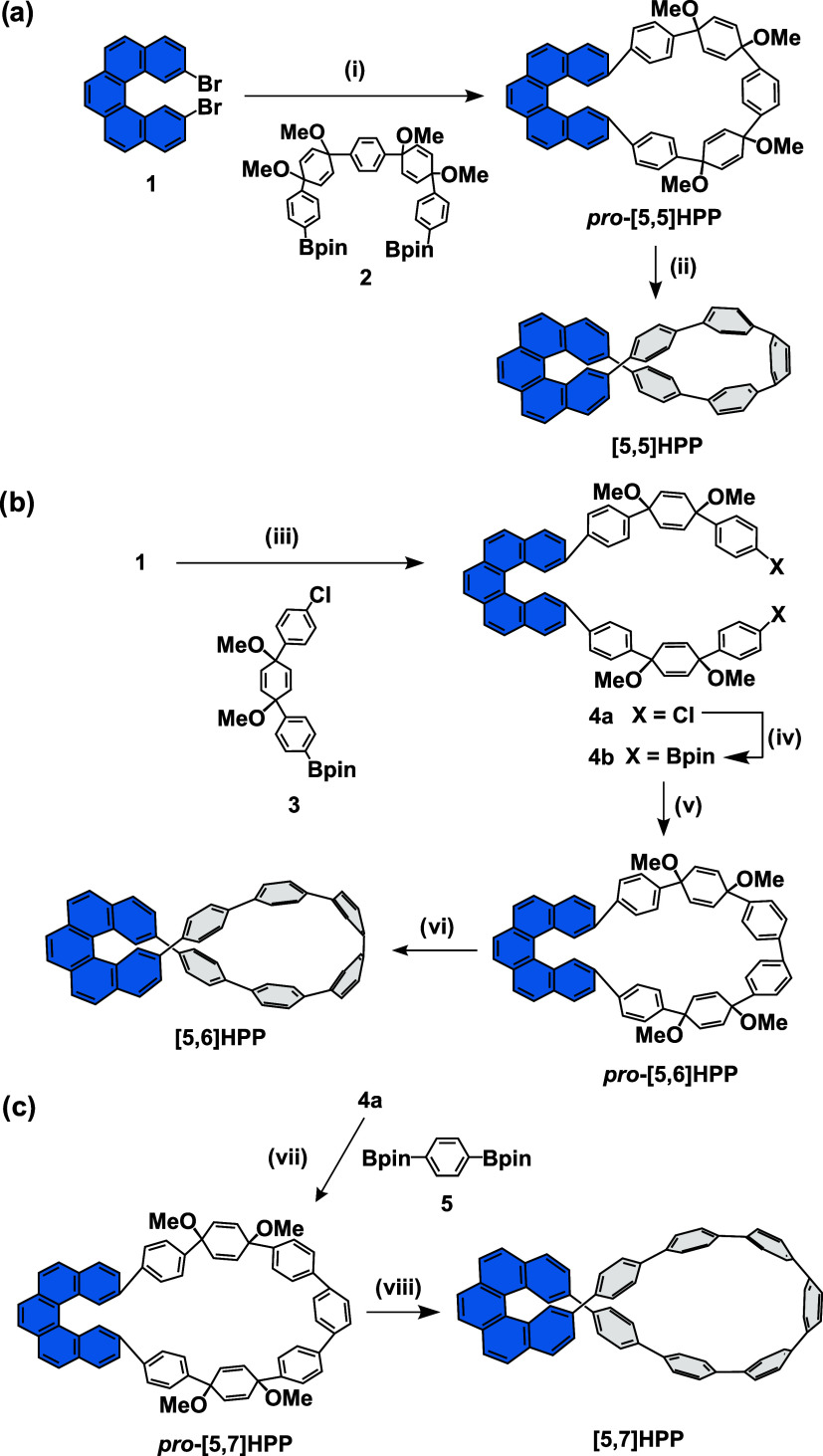
Synthesis of [5,*m*]­HPPs (*m* = 5–7)^a^

a (i) 1 equiv **2**, 0.2 equiv Pd SPhos G3, 8 equiv K_3_PO_4_, dioxane:H_2_O = 10:1, 80 °C, 16 h, 24%. (ii) 10 equiv H_2_SnCl_4_, THF, r.t., 1h, 80%. (iii) 2 equiv **3**, 0.1 equiv Pd SPhos G3, 8 equiv K_3_PO_4_, dioxane:H_2_O = 10:1, r.t., 24 h, 80%. (iv) 4 equiv (Bpin)_2_, 6.6 equiv KOAc, 0.05 equiv Pd_2_dba_3_, 0.2 equiv XPhos, dioxane, 80 °C, 16 h. (v) 5 equiv B­(OH)_3_, 20 equiv KF, 1 equiv Pd­(PPh_3_)_2_Cl_2_, THF:H_2_O = 10:1, r.t., 16 h, 52% over two steps. (vi) 2.2 equiv H_2_SnCl_4_, THF, r.t., 10 min, 97%. (vii) 1.1 equiv **5**, 0.1 equiv Pd SPhos G3, 8 equiv K_3_PO_4_, dioxane:H_2_O = 10:1, 80 °C, 16 h. (viii) 9 equiv H_2_SnCl_4_, THF, r.t., 30 min 25% over two steps.

The absorption and emission spectra of the [5,*m*]­HPP series were measured to elucidate the influence of the ring size on the optical properties (Figure 2). As expected, the calculated strain energy in individual compounds increases as the size of the macrocycle decreases (*E*_strain_, Table 1). All three HPPs exhibit a prominent absorption maximum at λ_abs_ = 340 nm characteristic of CPP systems.^35^ A distinct shoulder at >400 nm is observed in all spectra, corresponding to the HOMO–LUMO transition that reflects the curvature of the nanohoops. The smallest, most strained **[5,5]­HPP** displays the most red-shifted transition, resulting in a separate absorption band at 419 nm. The recorded emission maxima λ_em_ indicate a slight red-shift as the nanohoop size decreases, which is consistent with narrowing the HOMO–LUMO energy gap known for CPPs (Table 1). Notably, the fluorescence quantum yield (ϕ_fl_) increases with increasing ring size, suggesting that nonradiative decay channels are suppressed in larger HPPs more efficiently.

**Table 1:** Photophysical Properties and Racemization Kinetics Parameters

molecule	λ_em_, nm	ϕ_fl_	τ_fl_, ns	*E*_strain_ ^a^, kcal mol^–1^	Δ*G*_363_^‡^, kcal mol^–1^	Δ*H* ^‡^ ^b^, kcal mol^–1^	Δ*S* ^‡^ ^b^, cal mol^–1^ K^–1^	Δ*H*_en_^‡^ ^c^, kcal mol^–1^
**[5]helicene**	424^d^	0.04^d^	26^d^	0.0	24.4^e^	22.9	–4.1	24.7
**[5,5]HPP**	518	<0.01	n.a.^f^	61.9	25.5^g^ ± 0.070	26.8 ± 2.62	3.7 ± 7.92	26.2
**[5,6]HPP**	512	0.29	2.5	55.5	27.7^h^ ± 0.060	36.3 ± 1.24	23.4 ± 3.48	29.4
**[5,7]HPP**	498	0.46	2.6	50.0	28.5^h^ ± 0.030	32.5 ± 0.82	10.8 ± 2.25	30.4

a Calculated using homodesmotic reaction approach (B3LYP-D3/cc-pVTZ; see the SI).

b From Eyring analysis.

c Calculated (0 K, MN15/cc-pVTZ, see the SI for other functionals).

d Reported previously.^33,34^

e Calculated from Δ*G*_T_^‡^ = Δ*H* ^‡^ – TΔ*S* ^‡^.^11^

f Not available.

g Experimental value at 338 K.

h Experimental value.

**Figure 2:**
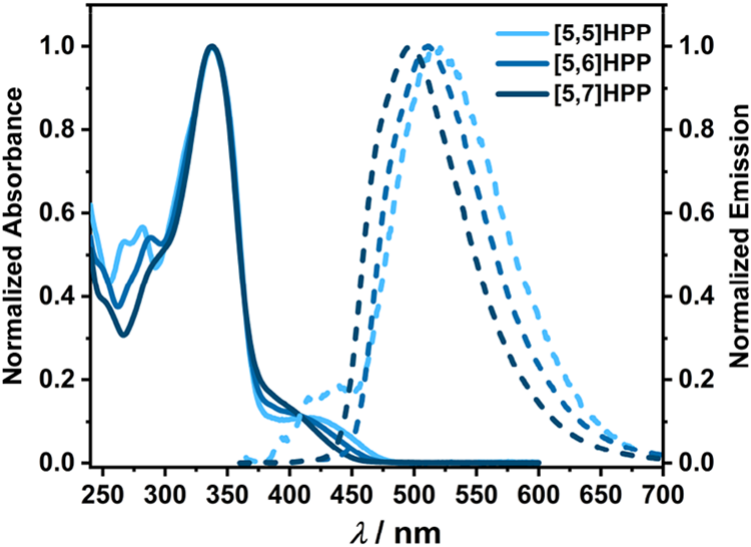
Absorption (solid lines) and emission (dotted lines) spectra of [5,*m*]­HPPs (*m* = 5–7) in dichloromethane (*c* ∼ 10^–6^ M).

We separated the enantiomers of all three nanohoops using recycling HPLC with a chiral stationary phase to obtain samples of individual enantiomers with >99% enantiomeric excess (ee). The configurations of the isolated enantiomers were assigned using electronic circular dichroism spectroscopy, quantum chemical calculations, and HPLC data (see Figures S29–S36, S53, S54, and S68–S71). We did not observe any racemization in the samples of **[5,6]­HPP** and **[5,7]­HPP** over several days at room temperature. However, we noticed partial conversion of the enantiomers of **[5,5]­HPP**. This indicated that the strain in the compounds affects their configurational stability. The racemization of [5,*m*]­HPPs was thus investigated by monitoring the evolution of sample *ee* as a function of time at elevated temperatures (65–100 °C). The process was stopped by rapid cooling of the sample solution at selected time intervals, and the concentration of enantiomers was quantified by analytical HPLC (details in SI). As expected, *ee* gradually decreased with time following the first-order rate law. The linear plots of ln­(ee_*t*_/ee_*t* = 0_) vs. *t* permitted extracting the corresponding enantiomerization rate constants (*k*_en_, Figures S38–S49 and Tables S13–S15). The activation free energies (Δ*G*_T_^‡^; *T* is temperature; Table 1) were then obtained from *k*_en_. In addition, we evaluated the Δ*G*_T_^‡^ at different temperatures (Figures 3 and S50–S52) to estimate if the process is driven by activation enthalpy (Δ*H* ^‡^) or entropy (Δ*S* ^‡^). Prolonged heating, however, decomposes the nanohoops, especially **[5,5]­HPP**. Additionally, the presence of oxygen facilitates decomposition, even at room temperature. Therefore, the experiments were performed with deoxygenated samples under argon. Thereby, we observed much faster racemization than decomposition.

**Figure 3:**
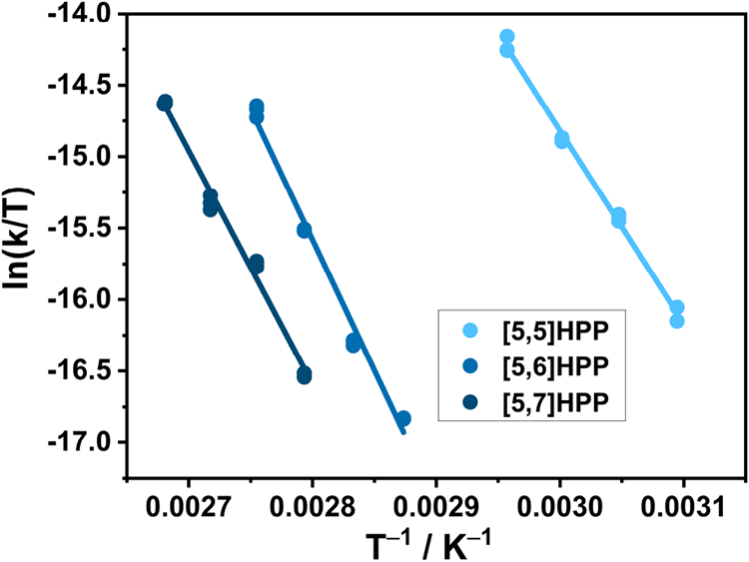
Eyring plots for [5,*m*]­HPPs.

The Δ*G*_T_^‡^ for all nanohoops is higher than that of the parent [5]­helicene (Δ*G*_298_^‡^ = 24.1 kcal mol^–1^) and demonstrates that helicene incorporation into a nanohoop increases its configurational stability. Clearly, the free energy barriers do not reach values of fjord-substituted [5]­helicenes, but they show a trend that correlates with the strain in the nanohoop. While the smallest, most strained **[5,5]­HPP** attains a modest increase in the configurational stability with Δ*G*_298_^‡^ = 25.7 kcal mol^–1^, the larger HPPs possess Δ*G*_298_^‡^ > 28.5 kcal mol^–1^, sufficient to maintain their enantiomeric purity for months at room temperature. The higher Δ*G*_T_^‡^ in the larger HPPs with fjord-free [5]­helicene appears counterintuitive. A large nanohoop with a smaller strain is expected to behave closer to [5]­helicene than a small, strained nanohoop. However, both the helicene and the curved *para*-phenylenes^36^ must change their geometry to reach the transition state structure. To gain additional insight into the enantiomerization pathway and the effect of the individual units in HPPs, we performed DFT calculations for all three [5,*m*]­HPPs, 2,13-diphenyl-[5]­helicene, and the parent [5]­helicene.

All [5,*m*]­HPPs adopt a variety of conformations. Therefore, we performed a thorough conformational search (see the SI), which confirmed that the lowest energy conformers of all HPPs display nonorientable π-electron systems (Figure S65), a feature observed previously in the crystal structures of **[6,7]­HPP** ^17^ and **[5,7]­HPP**.^32^ We tried to locate the transition states for each individual conformer. However, we found that the optimizations tend to relax to geometries that are close to *C*_s_ symmetry (Figure S67), the symmetry of the transition state structure of [5]­helicene.^10^ These possess the lowest energies and are therefore considered further. The calculated Δ*H* ^‡^ (Tables 1 and S25) shows the same trend as observed for experimental Δ*G*_T_^‡^, i.e., the larger the HPP, the higher the barrier. The calculations thus indicate that the enantiomerization process is driven by enthalpy, the same conclusion obtained from our experiments, and the activation entropy is small (Table S27). The low Δ*S* ^‡^ agrees well with the experimental data for [5]­helicene and its derivatives and validates our theoretical method.^8,11^ Although the experimental Δ*S* ^‡^ for **[5,5]­HPP** and **[5,7]­HPP** is relatively small, in good agreement with the calculations, the experimental values of Δ*H* ^‡^ and Δ*S* ^‡^ determined for **[5,6]­HPP** are too high. It appears that our Δ*H* ^‡^ and Δ*S* ^‡^ data may contain a systematic experimental error, especially those for **[5,6]­HPP**, which results in the enthalpy–entropy compensation. We note that estimating accurate enantiomerization Δ*S* ^‡^ using a limited range of temperatures can be challenging.^37,38^ Extending the temperature range in our case is, however, not feasible. At lower temperatures, the rate of the process is too low to be observable within a reasonable amount of time. At higher temperatures, we observe that a decomposition process starts competing with racemization, despite deoxygenating the solutions. Moreover, the additional source of error in our experiments can be the uncertainty in the experimental temperature, which is ±0.5 K. As a result, our Eyring analysis should be treated as semiquantitative at most, yet confirming that enantiomerization of [5,*m*]­HPPs is driven by Δ*H* ^‡^.

Importantly, we do not observe any correlation of the height of the barriers with structural parameters of [5]­helicene (Table S26), such as *d*_1–14_ or θ, as in the noncovalently stabilized fjord-substituted [5]­helicene derivatives.^8^ The barrier is thus likely determined by the strain in the transition states, which agrees with the calculated Δ*H* ^‡^. Therefore, we dissected the effect of strain in the structures of HPPs’ “ground” and transition states. Unlike in CPPs, the strain is not distributed equally in HPPs. Visualization of the strain energy distribution obtained by StrainViz (Figure S66) shows that the distant phenylenes are the most strained in HPPs, in agreement with our previous theoretical and experimental work on **[6,7]­HPP**.^17^ Calculations show that individual [5]­helicenes are strained by ∼ 1 kcal mol^–1^ in all HPPs (Table S24), a value calculated also for 2,13-diphenyl-[5]­helicene. Such destabilization does not reproduce the observed activation parameters. Comparison of the geometries and energies of [5]­helicene fragments (see SI) in the transition states portrays a clearer picture (Figure 4). It can be summarized as follows: (i) Their strain is larger in the transition states; (ii) the configurational stabilization of **[5,5]­HPP** can be explained nearly exclusively by the strain imposed on its [5]­helicene in the transition state (Table S24); (iii) the additional strain imposed by curved *para*-phenylenes must hinder the enantiomerization in the larger **[5,6]­HPP** and **[5,7]­HPP**. The CPP formally embeds the [5]­helicene, thus providing additional configurational stabilization, as the curved segment tends to adopt the *C*_s_ symmetry of the [5]­helicene transition state, which imparts additional strain on the most distant *para*-phenylenes. Since the larger macrocycles are less strained in their energy minimum, the increase in the strain is somewhat larger for those with longer *para*-phenylene segments. This suggests that the barrier for **[5,8]­HPP** should be even higher than that for **[5,7]­HPP**. We thus calculated the corresponding Δ*H* ^‡^ values to verify this prediction.

**Figure 4:**
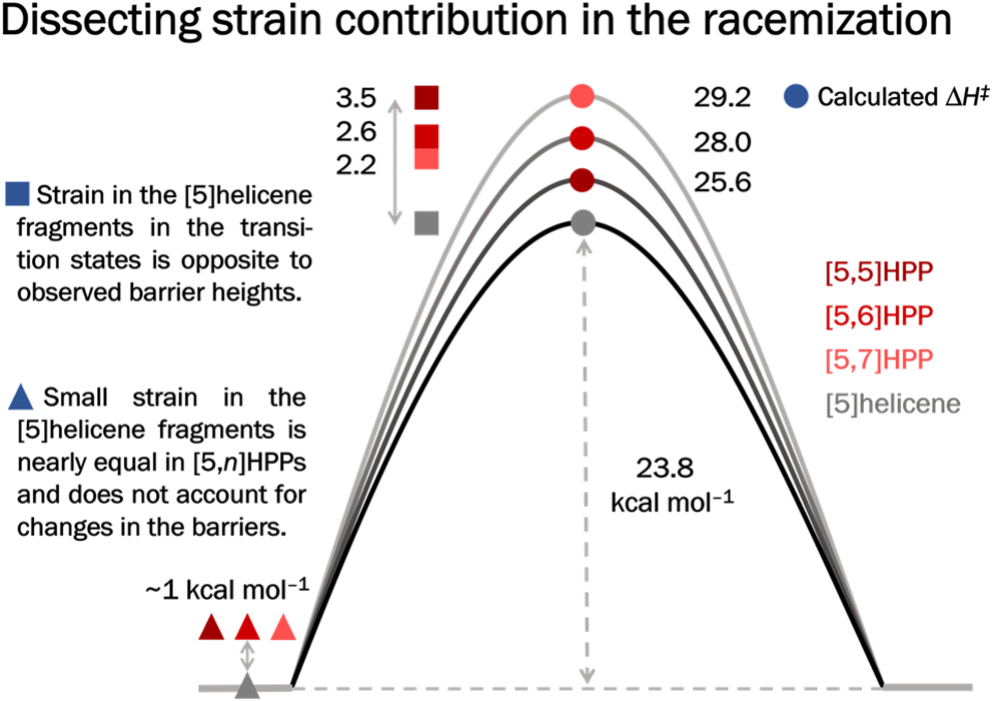
DFT analysis of the strain–barrier relationship (B3LYP-D3/cc-pVTZ energies in kcal mol^–1^; see SI).

Indeed, the calculated Δ*H* ^‡^ (Table S25) increased by an additional >1 kcal mol^–1^, which underpins our analysis. We note that the trend must saturate by enlarging the size of the nanohoops beyond a certain point because the strain energy in a large macrocycle will change only subtly by adding an extra phenylene. We speculate that the Δ*G* ^‡^ may start decreasing afterward, as we expect that enantiomerization of [5]­helicene in an infinitely large macrocycle resembles that of 2,13-diphenyl-[5]­helicene.

## Conclusions

In conclusion, we synthesized a series of chiral carbon nanohoops that embed [5]­helicene, separated their enantiomers, and investigated the effect of the nanohoop strain on their stereodynamic behavior. Thereby, we revealed a structure–property relationship that establishes design principles to control the stereodynamics of [5]­helicenes with a free fjord region, a concept that is straightforward to extend to other (hetero)­helicene derivatives.

## Experimental Section

### Materials and Methods

#### Chemicals and Reagents

All commercially available chemicals were purchased from Acros, Alfa Aesar, Apollo Scientific, Fluorochem, and Sigma-Aldrich and used without further purification. KOAc was dried in an oven at 130 °C overnight prior to use. Anhydrous solvents were purchased from Acros and stored over molecular sieves (4 Å).

Column chromatography was performed on silica gel P60 (40–63 μm) from Silicycle. Celite 545 (0.02–0.1 mm) from Supelco was used. TLC analysis was performed using Silica TLC plates (F254, Supelco Sigma-Aldrich) with visualization under ultraviolet light (254 and 365 nm). Automated flash chromatography was performed using a Biotage Isolera One.

Recycling gel permeation chromatography (GPC) and chiral high-performance liquid chromatography (HPLC) for [5,5]­HPP were performed on LaboAce 5060 from Japan Analytical Industry using JAIGEL-2HR Plus (20 × 600 mm, two columns in series) and Chiralpak IG (20 × 250 mm) as columns, respectively, and an appropriate solvent. Recycling GPC for [5,6]­HPP and [5,7]­HPP was performed with a Shimadzu Prominence System equipped with SDV preparative columns from Polymer Standards Service (two Shodex columns in series, 20 × 600 mm each, exclusion limit: 30,000 g mol–1) with chloroform as solvent. For preparative HPLC, a Shimadzu LC-20AP HPLC was used, equipped with a diode-array UV/vis detector (SPD-M20 A VP from Shimadzu, λ = 200–600 nm). Analytical HPLC analysis for all compounds was performed on a Shimadzu Prominence LC10-AT system equipped with a diode-array detector (DAD) and Chiralpak IG column (4.6 × 250 mm).

NMR experiments were performed at ambient temperature using Bruker AV II and III NMR spectrometers operating at 300, 400, 500, or 600 MHz proton frequencies. The instruments were equipped with a direct-observe 5 mm BBFO smart probe (400 and 500 MHz) and an indirect-detection 5 mm BBI probe (600 MHz). All probes were equipped with actively shielded z-gradients (10 A). The chemical shifts are reported in ppm relative to tetramethylsilane or referenced to the residual solvent peak, and the *J* values are given in Hz (±0.1 Hz). Standard Bruker pulse sequences were used, and the data were processed on Topspin 3.6 (Bruker) using 2-fold zero-filling in the indirect dimension. Structural assignments were made with additional information from the gCOSY, gHSQC, and gHMBC experiments.

High-resolution mass spectra (HRMS) for [5,5]­HPP (HRMS, FD+) were collected on an AccuTOF LC, JMS-T100LP Mass spectrometer (JEOL, Japan). HRMS for [5,6]­HPP and [5,7]­HPP were measured on a maX-isTM4G instrument from Bruker for HR-ESI-ToF MS.

UV–vis absorption was recorded for [5,5]­HPP on the spectrophotometer Shimadzu UV2700 equipped with a deuterium lamp (190–350 nm), a halogen lamp (330–900 nm), and a photomultiplier (Hamamatsu R928). For [5,6]­HPP and [5,7]­HPP, UV–vis absorption measurements were performed on a Jasco V-770 with a Jasco ETCR-762 Peltier cell holder thermostated at 25 °C in a 1.0 cm cuvette. Emission spectra for [5,5]­HPP were recorded with a Horiba Jobin Yvon Fluorolog-3 spectrofluorometer in an appropriate solvent in a 1.0 cm cuvette. Excitation and emission spectra for [5,6]­HPP and [5,7]­HPP were recorded using Jasco FP-8600 with Jasco ETC-815 Peltier cell holder thermostated at 25 °C in 1.0 cm cuvette.

Luminescence quantum yields of [5,5]­HPP were measured by using an integrating sphere setup. A 150 W xenon lamp coupled to a spectrometer (Solar, MSA-130) was used as an excitation source, providing a selection of excitation wavelengths. The excitation light and emission light were scattered diffusively in the integrating sphere. The respective emission and excitation spectra were subtracted to calculate the QY. The spectra were recorded by a CCD camera (Hamamatsu). Quantum yields for [5,6]­HPP and [5,7]­HPP were measured using a Jasco FP-8600 with ILFC-847 nitrogen purged integrating sphere in a 0.50 cm cuvette with direct and indirect excitation.

Circular dichroism was measured using a Jasco J-1500 with a Jasco PTC-517 Peltier thermostated cell holder at 25 °C in a 1.0 cm cuvette. Time-correlated single photon counting (TCSPC)-based emission lifetime measurements were performed on a LifeSpec II spectrometer (Edinburgh Instruments) employing a picosecond pulsed diode laser (ca. 60 ps pulse width).

### Preparation and Characterization of Compounds

9,12-Dibromo-[5]­helicene **1** was prepared according to a modified procedure by Moorthy et al.^27^ **2** and **3** were prepared according to a procedure by Jasti et al.^28,39^ **5** is commercially available (see SI).

#### *pro*-[5,5]­HPP

**1** (70 mg; 0.16 mmol; 1.00 equiv) and **2** (130 mg; 0.18 mmol; 1.10 equiv) were weighed in a Schlenk flask and dissolved in dioxane (120 mL) and H_2_O (12 mL). The resulting solution was deoxygenated by bubbling with Ar for 1 h. K_3_PO_4_ (270 mg; 1.3 mmol; 8.00 equiv) and SPhos Pd G3 (25 mg; 32 μmol; 0.2 equiv) were added, and the degassing continued for 30 min. The Schlenk flask was sealed and placed into an oil bath preheated to 80 °C. The reaction was stirred for 18 h at 80 °C. The reaction mixture was filtered through Celite, the flask and Celite were rinsed with dichloromethane and the volatiles were evaporated, and the crude product was purified using column chromatography (40% EtOAc in petroleum ether) followed by recycling GPC using chloroform. The product was isolated as a white solid (30 mg, 24%). ^**1**^ **H NMR** (300 MHz, CDCl_3_, 298 K, δ/ppm): 8.87 (d, *J* = 1.6 Hz, 2H), 8.03 (d, *J* = 8.3, 2H), 7.96 (d, *J* = 8.5 Hz, 2H), 7.89 (t, *J* = 4.3 Hz, 4H), 7.65 (dd, *J* = 8.3, 1.7 Hz, 2H), 7.34 (s, 8H), 7.12 (s, 4H), 6.31 (dd, *J* = 10.3, 2.4 Hz, 2H), 6.15–6.07 (m, 4H), 5.94 (dd, *J* = 10.1, 2.4 Hz, 2H), 3.44 (s, 6H), 3.33 (s, 6H). ^**13**^ **C NMR** (75 MHz, CDCl_3_, 298 K, δ/ppm): 142.0, 141.9, 141.4, 137.8, 134.5, 134.5, 133.3, 132.7, 132.6, 132.2, 131.8, 131.2, 128.7, 128.4, 127.9, 127.5, 127.3, 127.2, 126.8, 126.8, 126.6, 126.0, 126.0, 125.6, 75.5, 74.5, 52.2, 52.0. **HRMS (FD, +)**: *m*/*z* calcd. for C_56_H_44_O_4_: 780.3240, found: 780.3232.

#### [5,5]­HPP

Macrocycle ***pro*** **-[5,5]­HPP** (10 mg; 13 μmol; 1.00 equiv) was dissolved in THF (3 mL) and degassed for 30 min and H_2_SnCl_4_ (127 mM in THF; 1.0 mL; 0.13 mmol; 10 equiv) was added. The reaction mixture was stirred for 1 h at room temperature and quenched with 10% aqueous NaOH (1 mL). The resulting mixture was diluted with H_2_O (10 mL) and extracted with DCM (3 times 10 mL). The combined organic phases were washed with brine (10 mL) and dried with Na_2_SO_4_, and the volatiles were evaporated. The crude product was purified on recycling GPC using chloroform. The product was isolated as a yellow solid (7 mg; 80%). A reduced stability of this compound was observed in solution (slight decomposition overnight in CDCl_3_/ CD_2_Cl_2_). ^**1**^ **H NMR** (300 MHz, CD_2_Cl_2_, 298 K, δ/ppm): δ 8.86 (d, *J* = 1.7 Hz, 2H), 8.07 (d, *J* = 8.4 Hz, 2H), 7.98 (d, *J* = 8.5 Hz, 2H), 7.92–7.85 (m, 4H), 7.63 (td, *J* = 9.1, 2.0 Hz, 6H), 7.41 (dd, *J* = 9.3, 2.2 Hz, 4H), 7.33–7.20 (m, 12H). ^**13**^ **C NMR** (101 MHz, CD_2_Cl_2_, 298 K, δ/ppm): 139.9, 139.2, 139.0, 137.4, 137.0, 136.9, 133.2, 132.6, 132.2, 129.9, 129.5, 128.2, 128.1, 127.9, 127.8, 127.7, 127.5, 126.8, 126.6, 124.2. **HRMS (FD, +)**: *m*/*z* calcd. for C_52_H_32_: 656.2504, found: 656.2505.

#### 4a

Helicene **1** (100 mg; 0.23 mmol; 1.00 equiv) and **3** (207 mg; 0.46 mmol; 2.00 equiv) were added to a Schlenk flask, followed by dioxane (100 mL) and water (10 mL). The solution was degassed by bubbling with Ar for 1 h. K_3_PO_4_ (389 mg; 1.83 mmol; 8.00 equiv) and SPhos Pd G3 (18 mg; 22.9 μmol; 0.1 equiv) were added, and the degassing was further continued for 20 min. The Schlenk flask was sealed and stirred at room temperature for 24 h. The reaction mixture was filtered through Celite, the flask and Celite were rinsed with EtOAc and the volatiles were evaporated. The crude product was purified using column chromatography (10% EtOAc in cyclohexane). The product was isolated as a white solid (170 mg; 80%). Note: The reaction can be performed at elevated temperatures (>80 °C) in shorter reaction times (<3 h) but lower yields (<50%). ^1^H-spectrum of **4a** was measured after an overnight ^13^C-spectrum measurement and showed major decomposition leading to extra signals in ^13^C spectrum measured in CD_2_Cl_2_. Nevertheless, all major signals correspond to the formation of **4a**. The product was used for the next step without an analytically pure sample. ^**1**^ **H NMR** (400 MHz, CDCl_3_, 298 K, δ/ppm): 8.86 (d, *J* = 1.7, 2H), 8.07 (d, *J* = 8.4, 2H), 7.96 (d, *J* = 8.5, 2H), 7.90–7.85 (m, 4H), 7.80 (dd, *J* = 8.3, J = 1.8, 2H), 7.31 (d, *J* = 8.5, 4H), 7.25–7.20 (m, 8H), 7.13 (d, *J* = 8.7 Hz, 4H), 6.08–5.91 (m, 8H), 3.39 (s, 6H), 3.38 (s, 6H). ^**13**^ **C NMR** (126 MHz, CD_2_Cl_2_, 298 K, δ, ppm): 156.5, 139.7, 139.5, 137.4, 137.0, 133.4, 133.2, 133.1, 132.4, 131.5, 131.4, 130.2, 130.0, 129.4, 129.2, 128.5, 128.3, 127.8, 127.6, 127.5, 127.2, 126.9, 125.8, 112.0, 56.0, 50.9. **HRMS (ESI, +)**: *m*/*z* calcd. for C_62_H_48_Cl_2_NaO_4_ [M + Na]^+^: 949.2822, found: 949.2819; *m*/*z* calcd. for C_62_H_48_Cl_2_KO_4_ [M + K]^+^: 965.2561, found: 965.2552.

#### 4b

KOAc (152 mg; 1.55 mmol; 6.6 equiv) was weighed while still hot into a dry Schlenk flask, the flask was evacuated and placed into an oil bath at 130 °C for 1 h after which it was cooled down to room temperature under a flow of Ar. Afterward, **4a** (218 mg; 235 μmol; 1.00 equiv), (Bpin)_2_ (241 mg; 940 μmol; 4 equiv), XPhos (22.4 mg; 47 μmol; 0.2 equiv), and Pd_2_dba_3_ (11 mg; 11.7 μmol; 0.05 equiv) were added. The flask was evacuated and filled with Ar three times. In a separate dry flask, dioxane was degassed by bubbling it with Ar for 1 h. Degassed dioxane (10 mL) was added to dissolve all components, and the resulting solution was further degassed by bubbling with Ar for 15 min. The Schlenk flask was sealed with a glass stopper and placed into an oil bath preheated to 100 °C. The reaction was stirred for 16 h at 100 °C. The reaction mixture was cooled down to room temperature and filtered through Celite, the flask and Celite were rinsed with EtOAc and the volatiles were evaporated affording a thick orange oil. The product was precipitated with EtOH, filtered off, washed with EtOH, dried in vacuo, and used without further purification. A low stability of this compound was observed in solution (full decomposition in CDCl_3_ overnight).

#### *pro*-[5,6]­HPP

To **4b** (53 mg; 47.7 μmol; 1 equiv), Pd­(PPh_3_)_2_Cl_2_ (34 mg; 47.7 μmol; 1 equiv) and B­(OH)_3_ (15 mg; 239 μmol; 5 equiv) were added. The solids were dissolved in THF (50 mL) and stirred vigorously for 10 min. KF (55 mg; 954 μmol; 20 equiv) was dissolved in H_2_O (5 mL) and added. The reaction was stirred vigorously at room temperature open to air for 16 h. The mixture was filtered through Celite, the flask and Celite were rinsed with EtOAc and concentrated to give a yellow-orange solid. The crude product was dissolved in chloroform, passed through a short plug of silica and purified using recycling GPC. The product was isolated as an off-yellow solid (30 mg; 52% over 2 steps). ^13^C NMR spectrum of sufficient quality was not obtained due to decomposition of the material during measurement. The listed ^13^C NMR shifts were assigned by using 2D-NMR techniques. Note: Usage of catalytic amounts of Pd­(PPh_3_)_2_Cl_2_ results in significantly lower yields. ^**1**^ **H NMR** (500 MHz, CDCl_3_, 298 K, δ/ppm): 8.40 (d, *J* = 1.7 Hz, 2H), 7.94 (d, *J* = 8.4 Hz, 2H), 7.90 (d, *J* = 8.6 Hz, 2H), 7.81 (d, *J* = 8.6 Hz, 2H), 7.80 (s, 2H), 7.51 (dd, *J* = 8.3, *J* = 1.8 Hz, 2H), 7.32 (s, 8H), 7.12 (d, *J* = 8.7 Hz, 4H), 7.07 (d, *J* = 8.5 Hz, 4H), 6.35 (dd, *J* = 10.3, 2.4 Hz, 2H), 6.24 (dt, *J* = 10.3, 1.9 Hz, 4H), 6.12 (dd, *J* = 10.2, 2.4 Hz, 2H), 3.46 (s, 6H), 3.30 (s, 6H). ^**13**^ **C NMR** (126 MHz, CDCl_3_, 298 K, δ/ppm): 141.6, 141.3, 141.2, 140.6, 138.8, 134.5, 134.0, 133.5, 132.6, 132.4, 132.2, 131.7, 128.1, 127.5, 127.4, 127.3, 126.9, 126.9, 126.8, 126.7, 125.8, 75.8, 75.0, 52.5, 51.9. **HRMS (ESI, +):** *m*/*z* calcd. for C_62_H_48_AgO_4_ [M + Ag]^+^: 963.2598, found: 963.2584.

#### [5,6]­HPP

Macrocycle ***pro*** **-[5,6]­HPP** (30 mg; 0.35 mmol; 1.00 equiv) was dissolved in THF (5 mL), and H_2_SnCl_4_ (127 mM in THF; 0.6 mL; 0.77 mmol; 2.2 equiv) was added. The reaction mixture was stirred for 10 min at room temperature and quenched with 10% aqueous NaOH (1 mL). The resulting mixture was diluted with H_2_O (10 mL) and extracted with DCM (3 times 10 mL). The combined organic phases were washed with brine (10 mL) and dried with Na_2_SO_4_, and volatiles were evaporated. The crude product was purified by column chromatography (50% toluene in *n*-heptane). The product was isolated as a yellow solid (25 mg; 97%). Note: Prolonged reaction times resulted in significantly lower yields. ^**1**^ **H NMR** (500 MHz, CD_2_Cl_2_, 298 K, δ/ppm): 8.82 (d, *J* = 1.7, 2H), 8.01 (d, *J* = 8.4, 2H), 7.91 (d, *J* = 8.5, 2H), 7.85–7.79 (m, 4H), 7.59 (dd, *J* = 8.4, *J* = 1.8, 2H), 7.54 (dd, *J* = 9.0, *J* = 2.2, 2H), 7.48 (dd, *J* = 9.0, *J* = 2.1, 2H), 7.46–7.35 (m, 12H), 7.29 (d, *J* = 8.6 Hz, 4H), 7.24 (d, *J* = 8.5 Hz, 4H). ^**1**^ **H NMR** (600 MHz, toluene-d_8_, 298 K, δ/ppm): 9.06 (s, 2H), 7.87 (d, *J* = 8.3, 2H), 7.73 (d, *J* = 8.4, 2H), 7.65 (d, *J* = 8.4, 2H), 7.63 (d, *J* = 8.4, 2H), 7.61 (s, 2H), 7.49–7.45 (m, 2H), 7.44–7.37 (m, 4H), 7.34 (d, *J* = 8.3, 4H), 7.20 (d, *J* = 8.9, 2H), 7.12–7.04 (m, 4H), 7.02 (d, *J* = 8.3, 4H), 6.89 (d, *J* = 8.9, 4H). ^**13**^ **C NMR** (151 MHz, toluene-d_8_, 298 K, δ/ppm): 140.6, 139.2, 138.7, 138.6, 138.5, 138.0, 137.7, 137.7, 137.2, 133.7, 133.0, 132.8, 129.8, 129.8, 128.8, 128.7, 128.3, 128.1, 128.0, 127.9, 127.7, 127.1, 126.6, 126.2, 125.9. **HRMS (ESI, +)**: *m*/*z* calcd. for C_58_H_36_Ag [M + Ag]^+^: 839.1862, found: 839.1857.

#### [5,7]­HPP

**4a** (100 mg; 0.108 mmol; 1.00 equiv) and **5** (37 mg; 0.113 mmol; 1.05 equiv) were weighed in a Schlenk flask and dissolved in dioxane (100 mL) and H_2_O (10 mL). The resulting solution was degassed by bubbling with Ar for 1 h. K_3_PO_4_ (183 mg; 0.862 mmol; 8.00 equiv) and SPhos Pd G3 (8.4 mg; 10.8 μmol; 0.1 equiv) were added, and the degassing continued for 30 min. The Schlenk flask was sealed and placed into an oil bath preheated to 80 °C. The reaction was stirred for 18 h at 80 °C. The reaction mixture was filtered through Celite, the flask and Celite were rinsed with dichloromethane, and the volatiles were evaporated, and the crude product was dried in a high vacuum. The crude was dissolved in CHCl_3_, passed through a short silica plug and injected into recycling GPC (CHCl_3_). The main peak was collected and concentrated, and the presence of the intermediate macrocycle was confirmed by NMR. This product was used without further purification to avoid decomposition. The residue was dissolved in THF (5 mL), and H_2_SnCl_4_ (127 mM in THF; 1.9 mL; 9 equiv) was added. The reaction mixture was stirred for 30 min at room temperature and quenched with 10% aqueous NaOH (2.5 mL). The resulting mixture was diluted with H_2_O (50 mL) and extracted with DCM (4 times 25 mL). The combined organic phases were washed with brine (25 mL) and dried with Na_2_SO_4_, and volatiles were evaporated. The crude product was purified by column chromatography (50% toluene in *n*-heptane) followed by recycling GPC (CHCl_3_). The product was isolated as a yellow solid (28 mg; 25%). ^**1**^ **H NMR** for ***pro*** **-[5,7]­HPP:** (300 MHz, CDCl_3_, 298 K, δ/ppm): 8.24 (d, *J* = 1.8 Hz, 2H), 7.89 (dd, *J* = 11.6, 8.5 Hz, 4H), 7.77 (d, 4H), 7.64 (s, 4H), 7.51–7.38 (m, 10H), 7.11–6.94 (m, 8H), 6.35 (dd, *J* = 10.3, 2.4 Hz, 2H), 6.21–6.05 (m, 4H), 5.84 (dd, *J* = 10.2, 2.4 Hz, 2H), 3.46 (s, 6H), 3.26 (s, 6H). The spectral data of the synthesized [5,7]­HPP match the data reported by Jiang et al.^32^ ^**1**^ **H NMR** for **[5,7]­HPP** (500 MHz, CDCl_3_, 298 K, δ/ppm): 8.97 (s, 2H), 8.07 (d, *J* = 8.4, 2H), 7.97 (d, *J* = 8.5, 2H), 7.91–7.85 (m, 4H), 7.70 (dd, *J* = 8.4, *J* = 1.8, 2H), 7.56 (dd, *J* = 9.0, *J* = 2.1, 2H), 7.52 (d, *J* = 8.1, 4H), 7.49–7.46 (m, 4H), 7.46–7.42 (m, 10H), 7.41–7.34 (m, 8H). **HRMS (MALDI, DCTB matrix, +):** *m*/*z* calcd. for C_64_H_40_ [M]^+^: 808.3125, found: 808.3123.

## Supplementary Material



## Data Availability

The data underlying this study are available in the published article and its Supporting Information.

## Notes

Experimental methods, compound characterization, kinetic experiments, and spectroscopic and computational data. (PDF)
