# Supplementary material for: How do Nanohoops Exercise Their Strain in [5]Helicene Racemization?
Source: J Org Chem. 2025 Nov 10;90(46):16607–14. doi: 10.1021/acs.joc.5c02280 (PMC12645475; doi:10.1021/acs.joc.5c02280)
Supplement: Supplementary file 1 [file jo5c02280_si_001.pdf]

Supporting Information

How do Nanohoops Exercise their Strain in [5]helicene Racemization?

Kovida Kovida,[a] Juraj Malinčík,[a] Thijs de Groot,[a] Tomáš Šolomek*[a]

a Van 't Hoff Institute for Molecular Sciences
University of Amsterdam
Science Park 904, 1098 XH Amsterdam, The Netherlands
E-mail: t.solomek@uva.nl

Table of Contents

Experimental Part S3

NMR and MS spectra S3

Preparative HPLC S21

Stability of [5,n]HPPs S24

Racemization Kinetics S25

Optical and Chiroptical Properties S39

DFT Calculations S45

Strain Energy and Activation Enthalpy Calculations S47

TD-DFT S52

CD Spectra S52

Excited-state Lifetime Measurements S55

Cartesian coordinates S58

References S70

Experimental Part

NMR and MS spectra

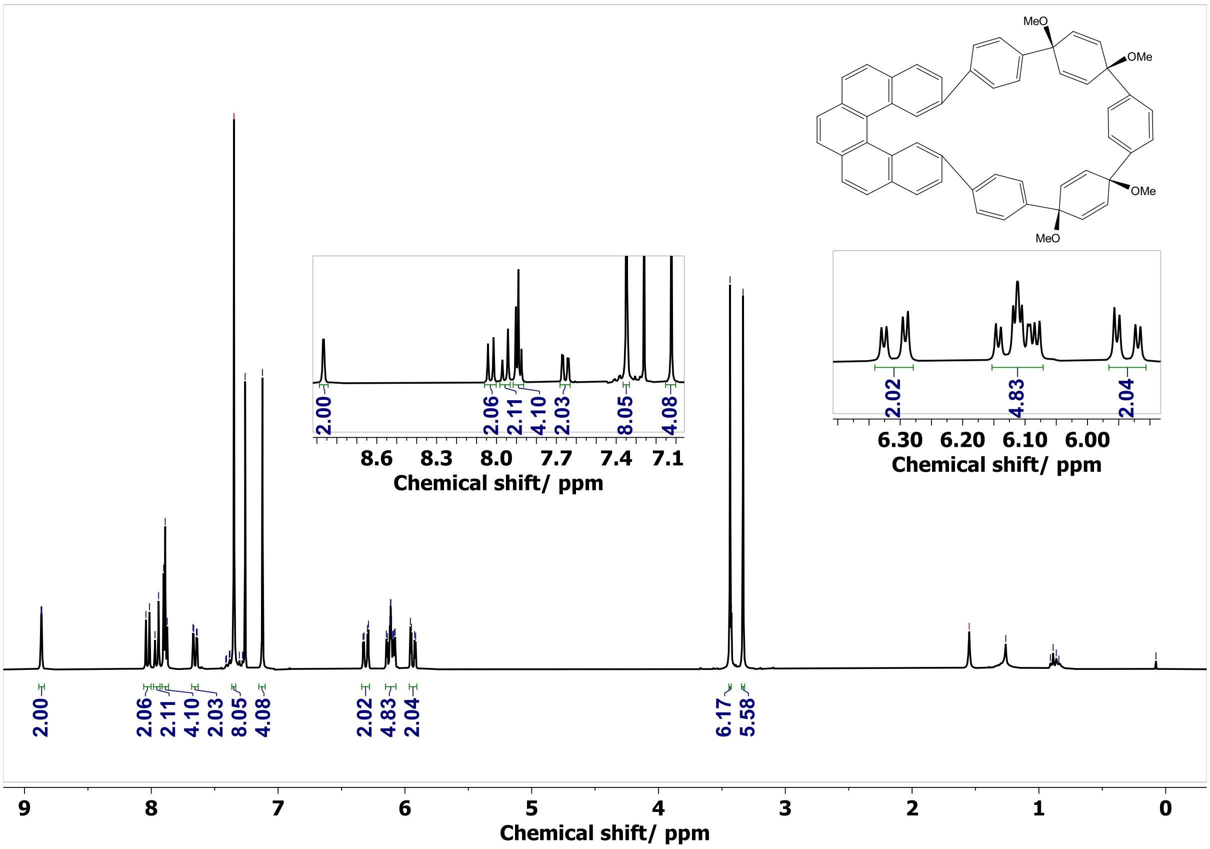

Figure S1. ^{1}H-NMR (300 MHz, $CDCl_3$, 298 K) spectrum of ***pro*-[5,5]HPP**.

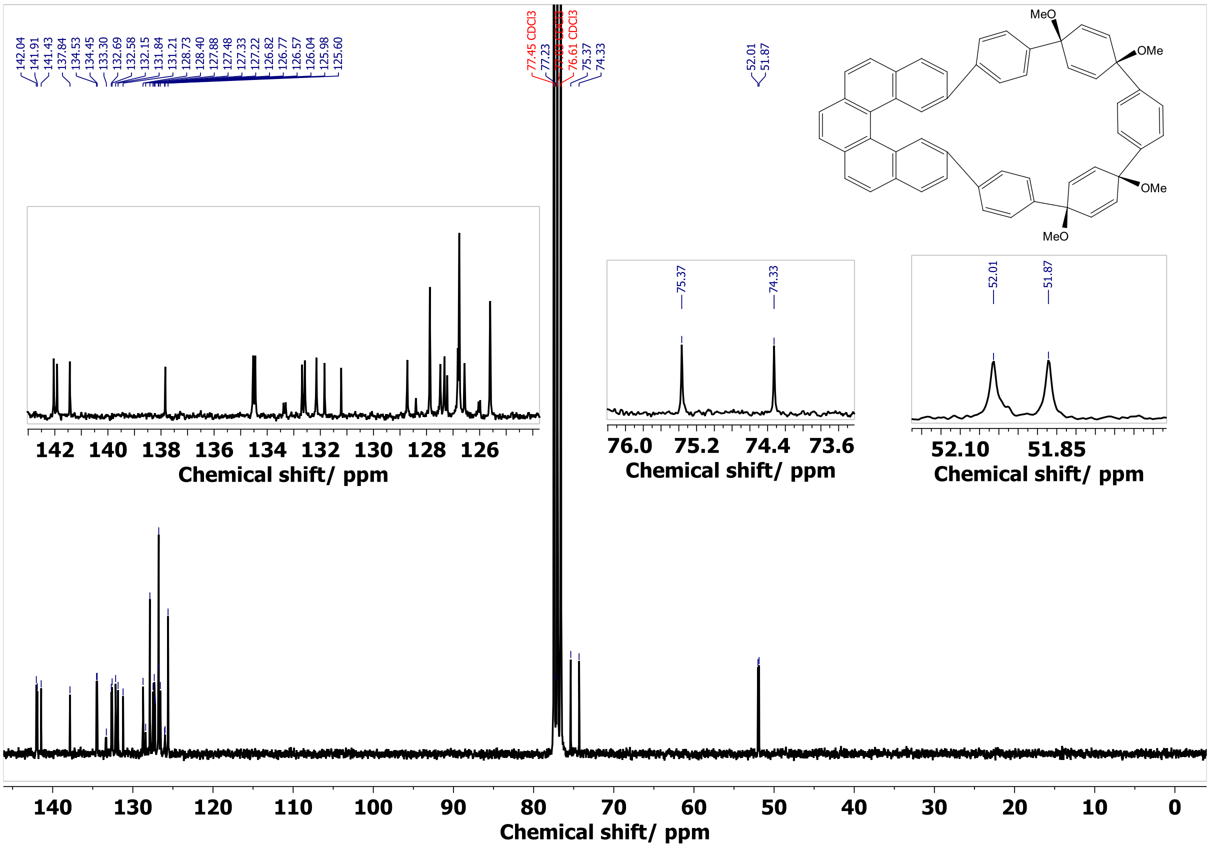

Figure S2. $^{13}C\{^{1}H\}$-NMR (126 MHz, $CDCl_3$, 298 K) spectrum of ***pro*-[5,5]HPP**.

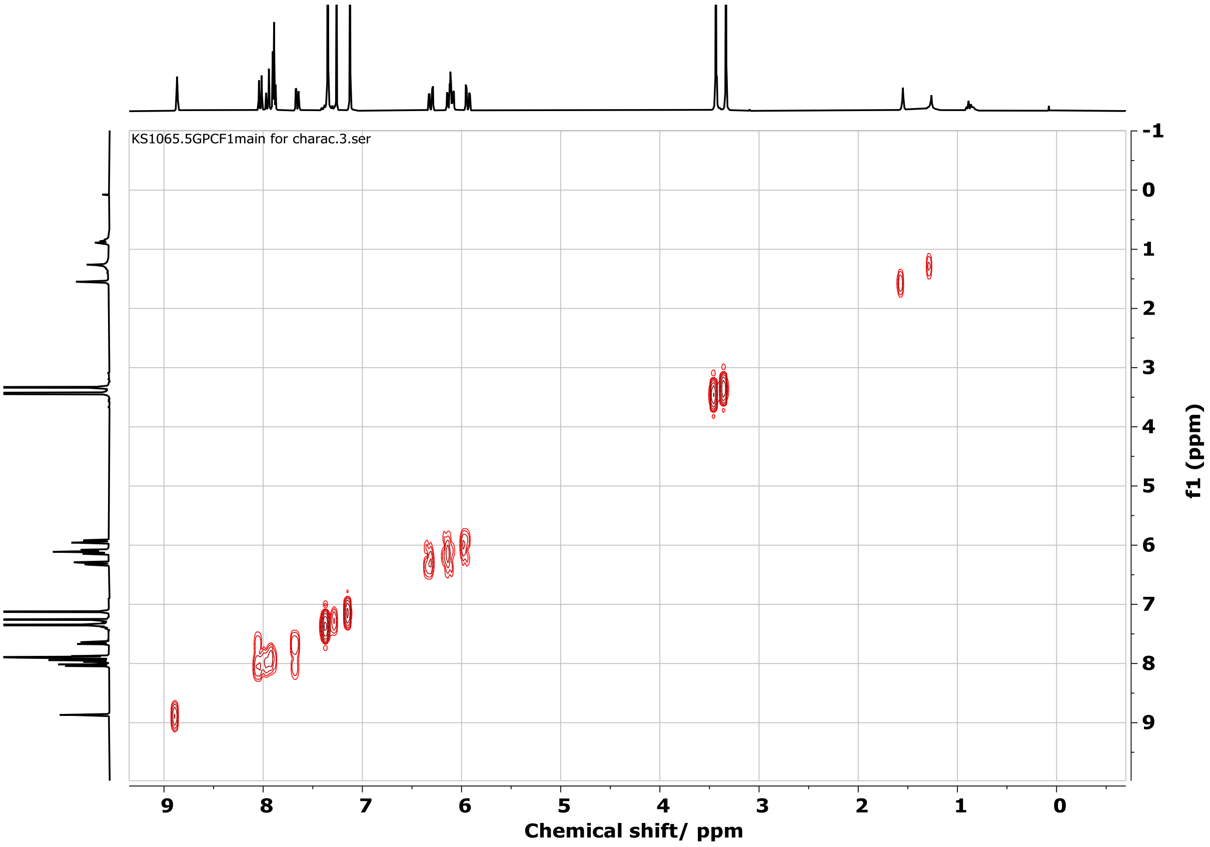

Figure S3. ^{1}H–^{1}H COSY ($CDCl_3$, 298 K) spectrum of ***pro*-[5,5]HPP**.

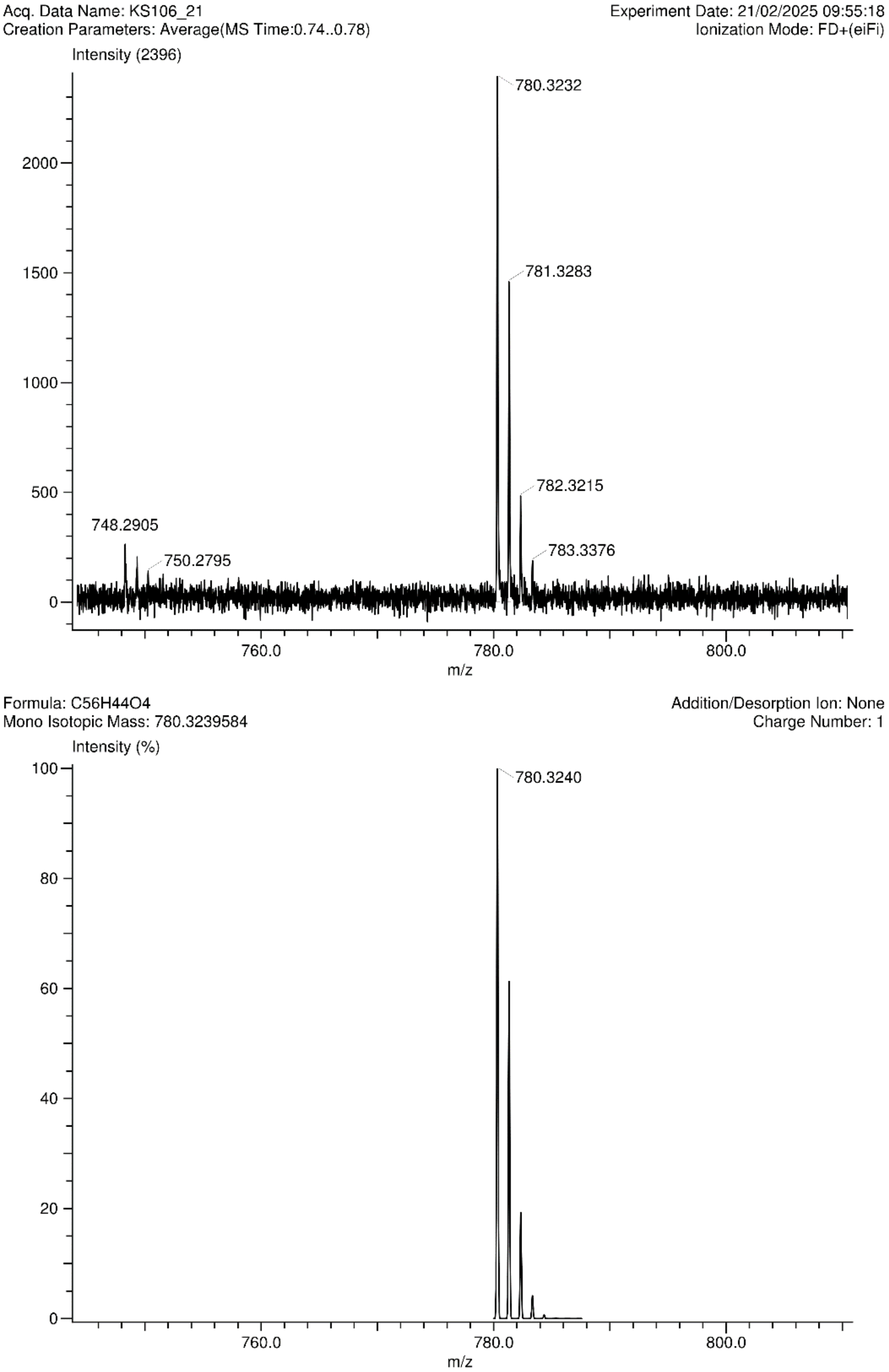

Figure S4. HR-MS (FD+) spectrum of ***pro*-[5,5]HPP**.

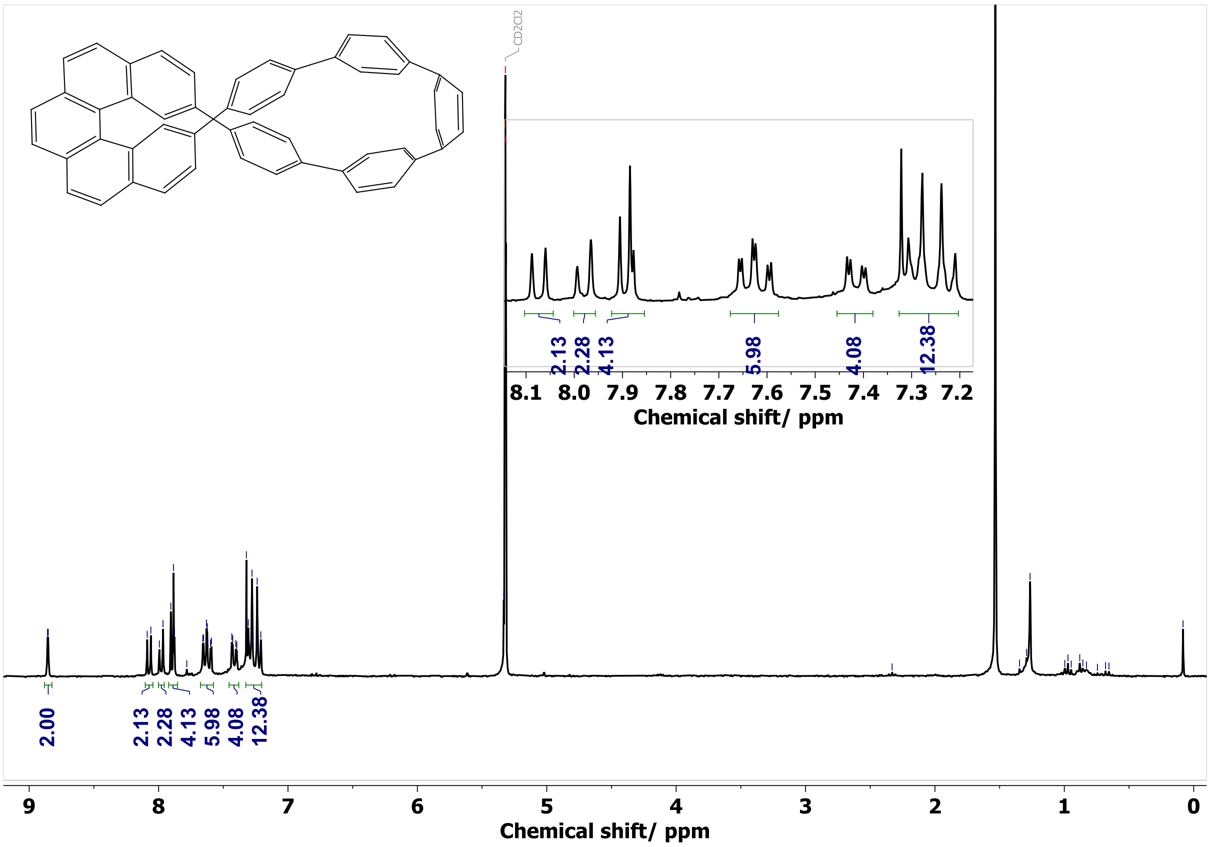

Figure S5. ^{1}H-NMR (300 MHz, CD_2Cl_2, 298 K) spectrum of **[5,5]HPP**.

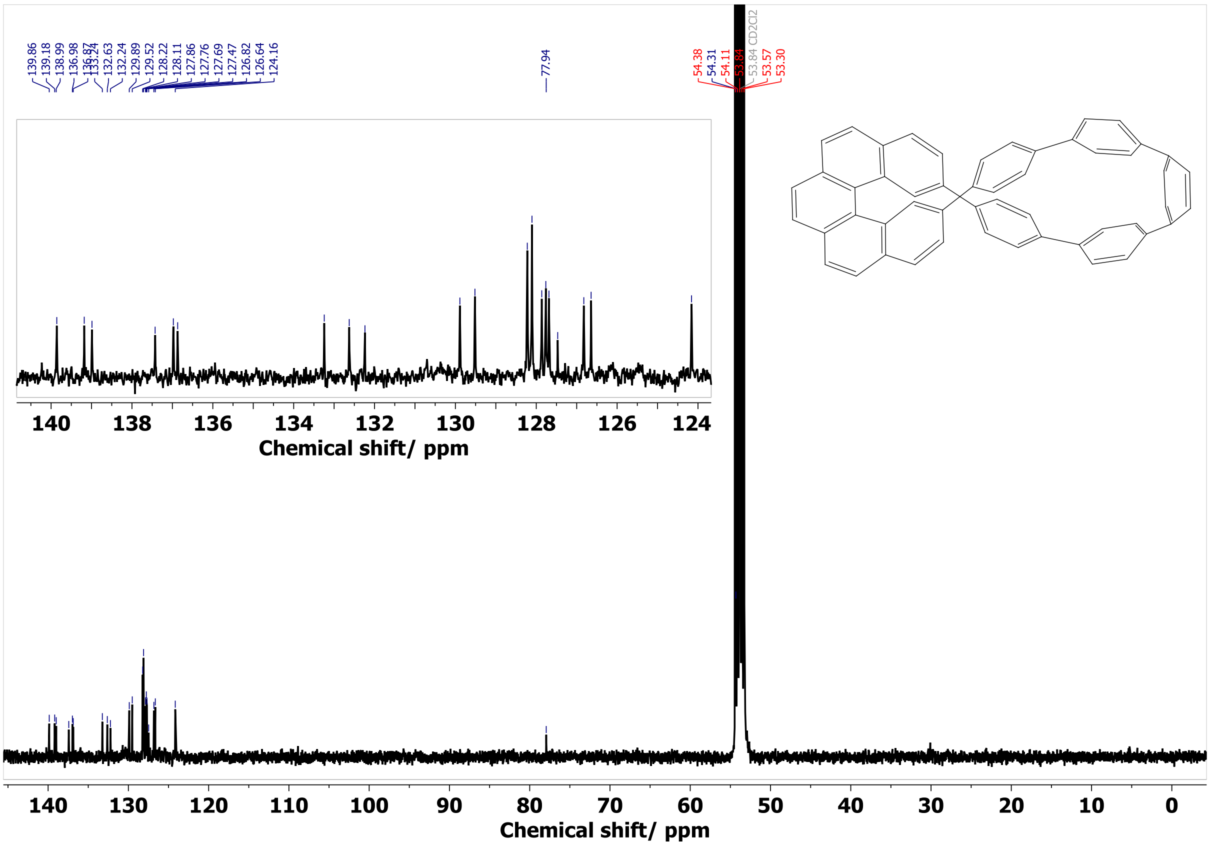

Figure S6. $^{13}C\{^{1}H\}$-NMR (126 MHz, CD_2Cl_2, 298 K) spectrum of **[5,5]HPP**.

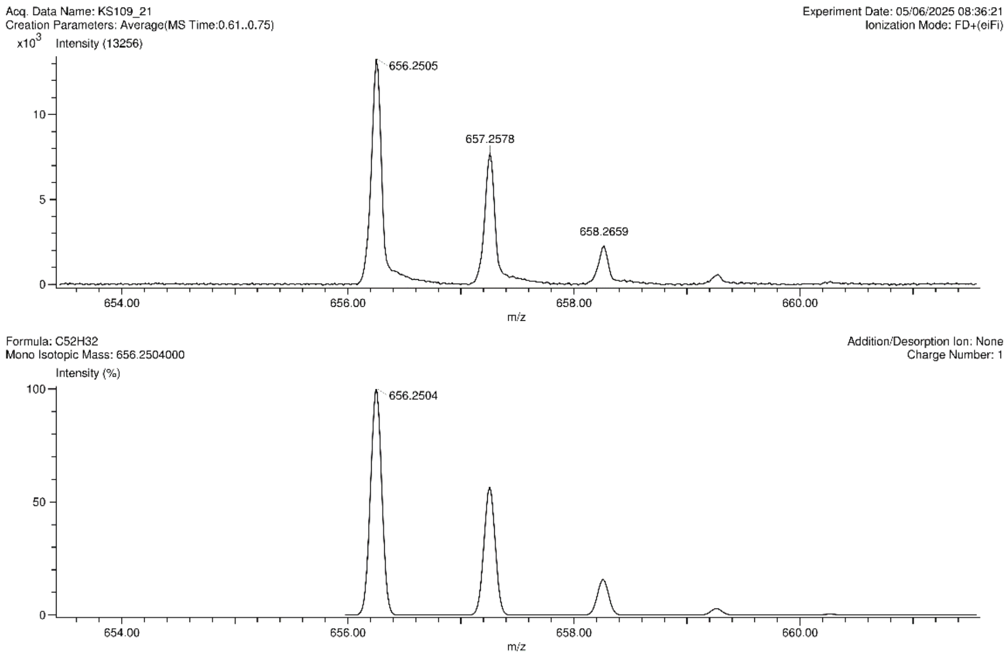

Figure S7. HR-MS (FD+) spectrum of **[5,5]HPP**.

^{1}H NMR (400 MHz, $CDCl_3$) δ 8.86 (d, J = 1.7 Hz, 2H), 8.07 (d, J = 8.4 Hz, 2H), 7.96 (d, J = 8.5 Hz, 2H), 7.90 – 7.85 (m, 4H), 7.80 (dd, J = 8.3, 1.8 Hz, 2H), 7.31 (d, J = 8.5 Hz, 4H), 7.25 – 7.20 (m, 8H), 7.13 (d, J = 8.7 Hz, 4H), 6.08 – 5.91 (m, 8H), 3.39 (s, 6H), 3.38 (s, 6H).

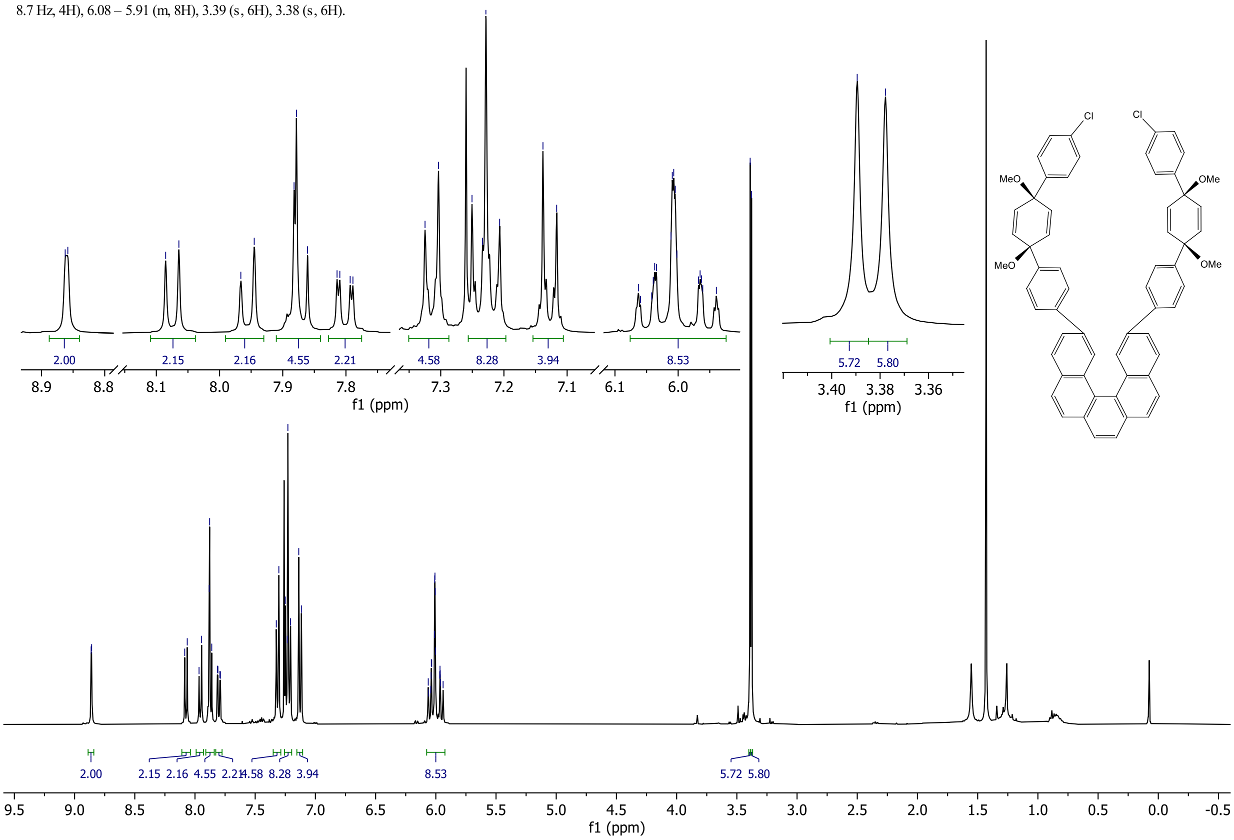

Figure S8. ^{1}H-NMR (400 MHz, $CDCl_3$, 298 K) spectrum of **4a**.

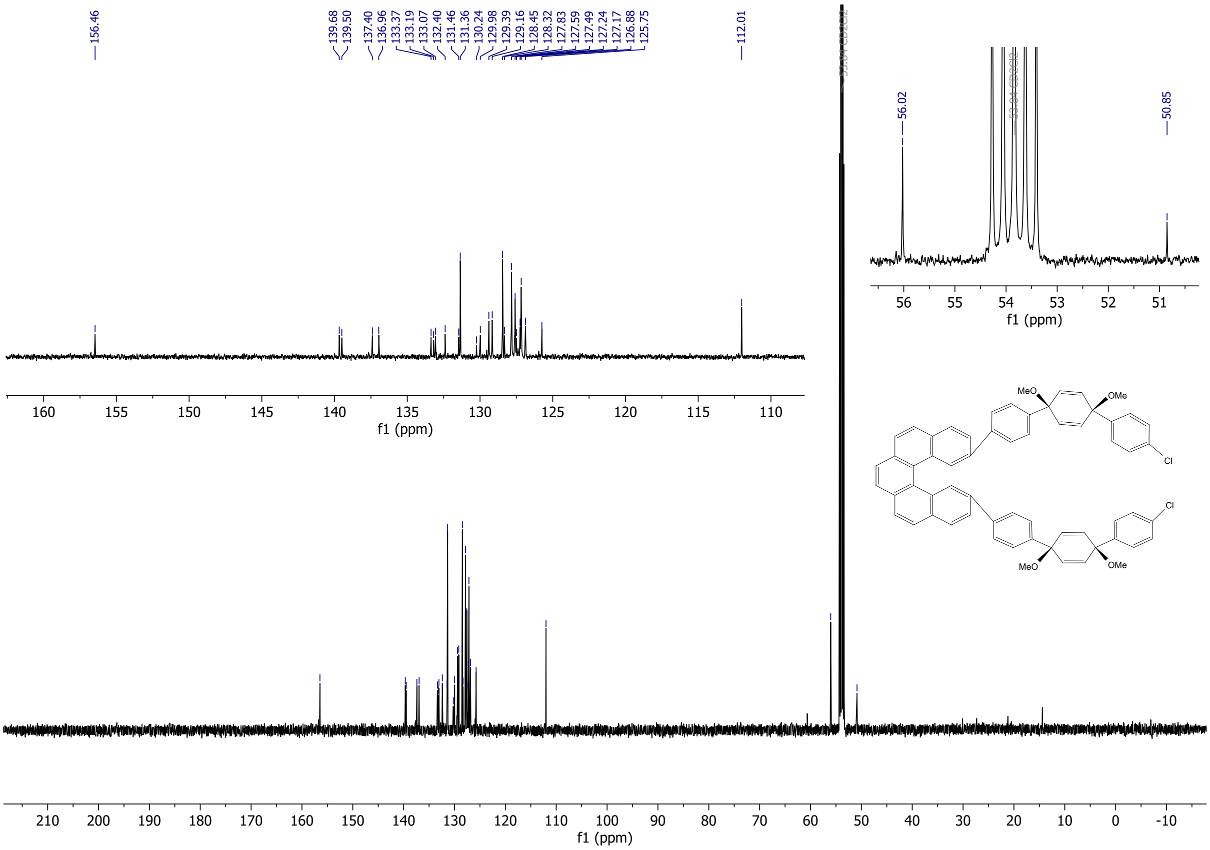

Figure S9. $^{13}C\{^{1}H\}$-NMR (126 MHz, CD_2Cl_2, 298 K) spectrum of **4a**.

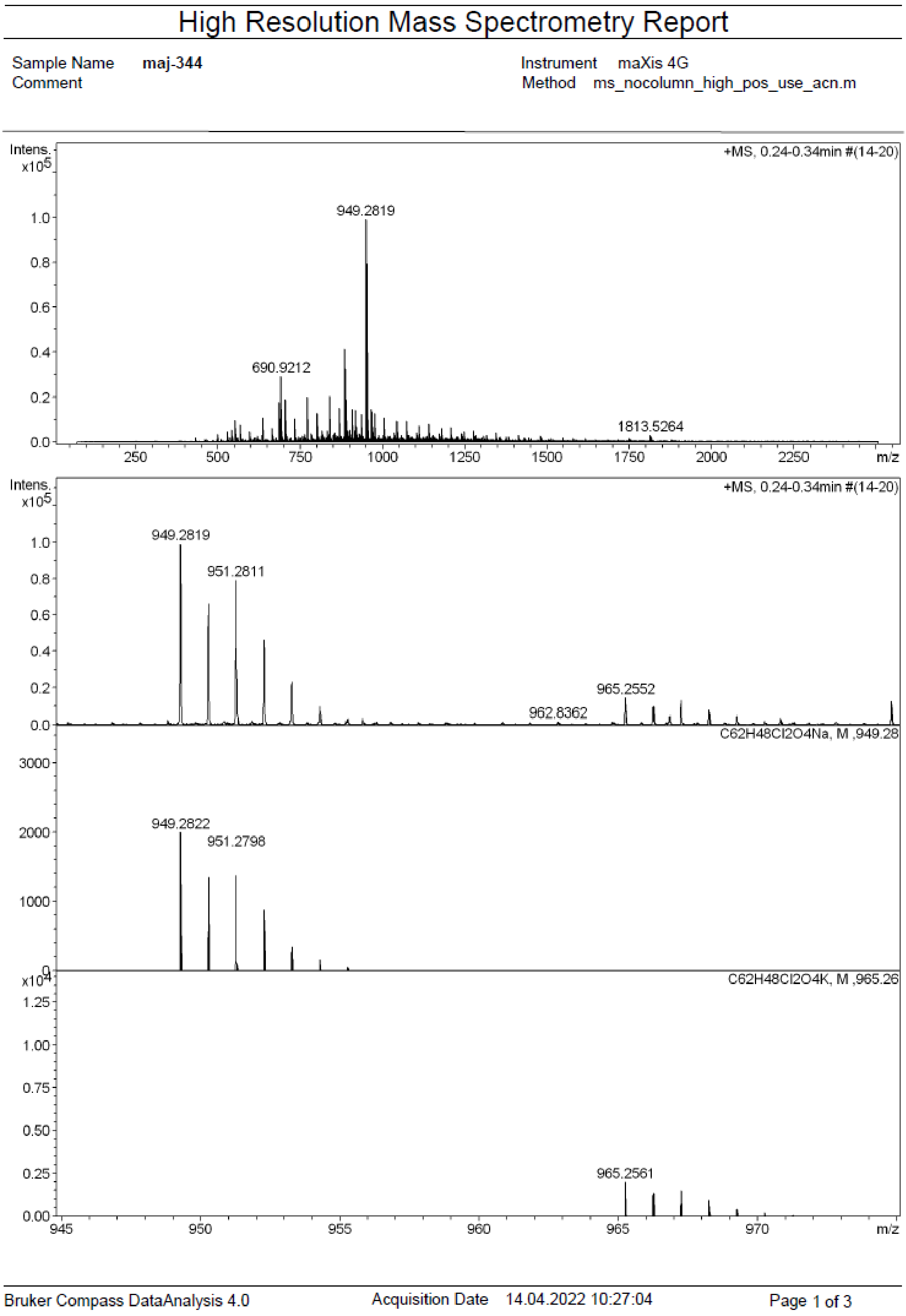

Figure S10. HR-MS (ESI, +) spectrum of **4a** $[M+Na]^+$ and $[M+K]^+$.

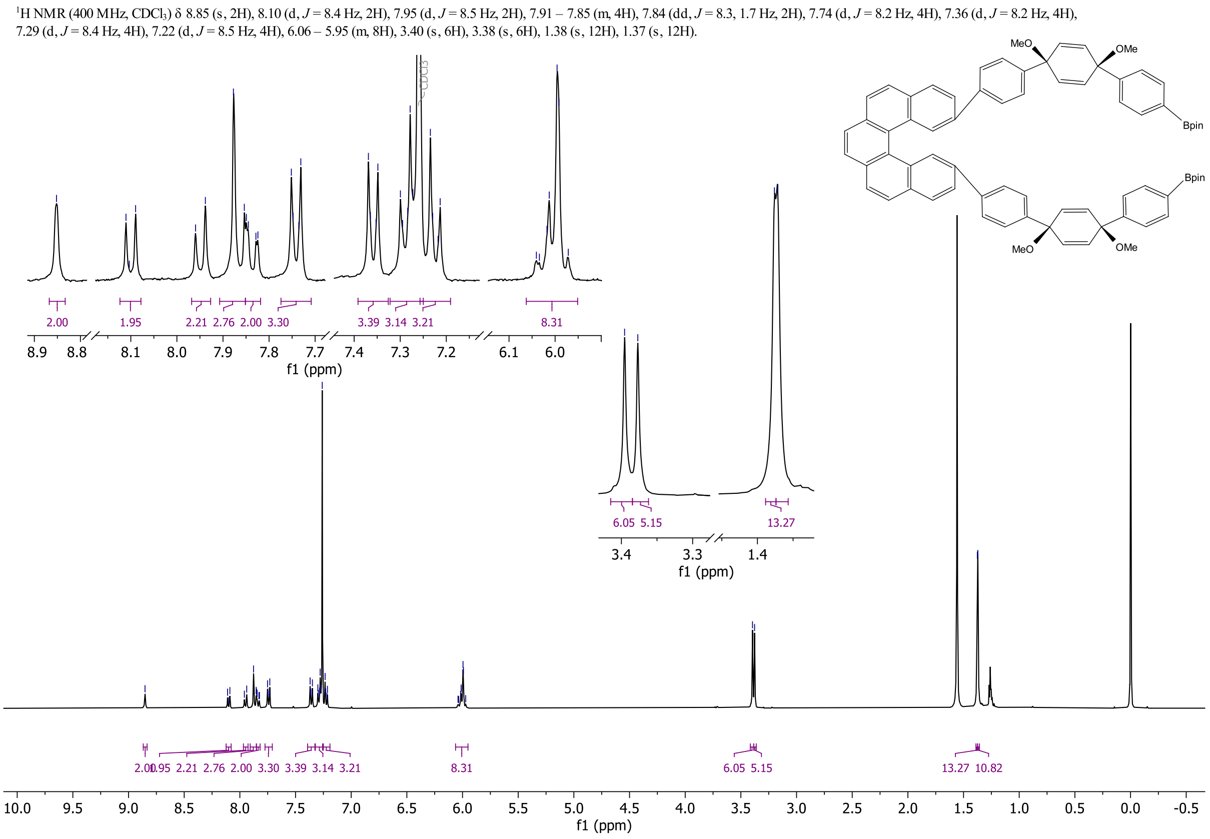

Figure S11. ^{1}H-NMR (500 MHz, $CDCl_3$, 298 K) spectrum of precipitated **4b**.

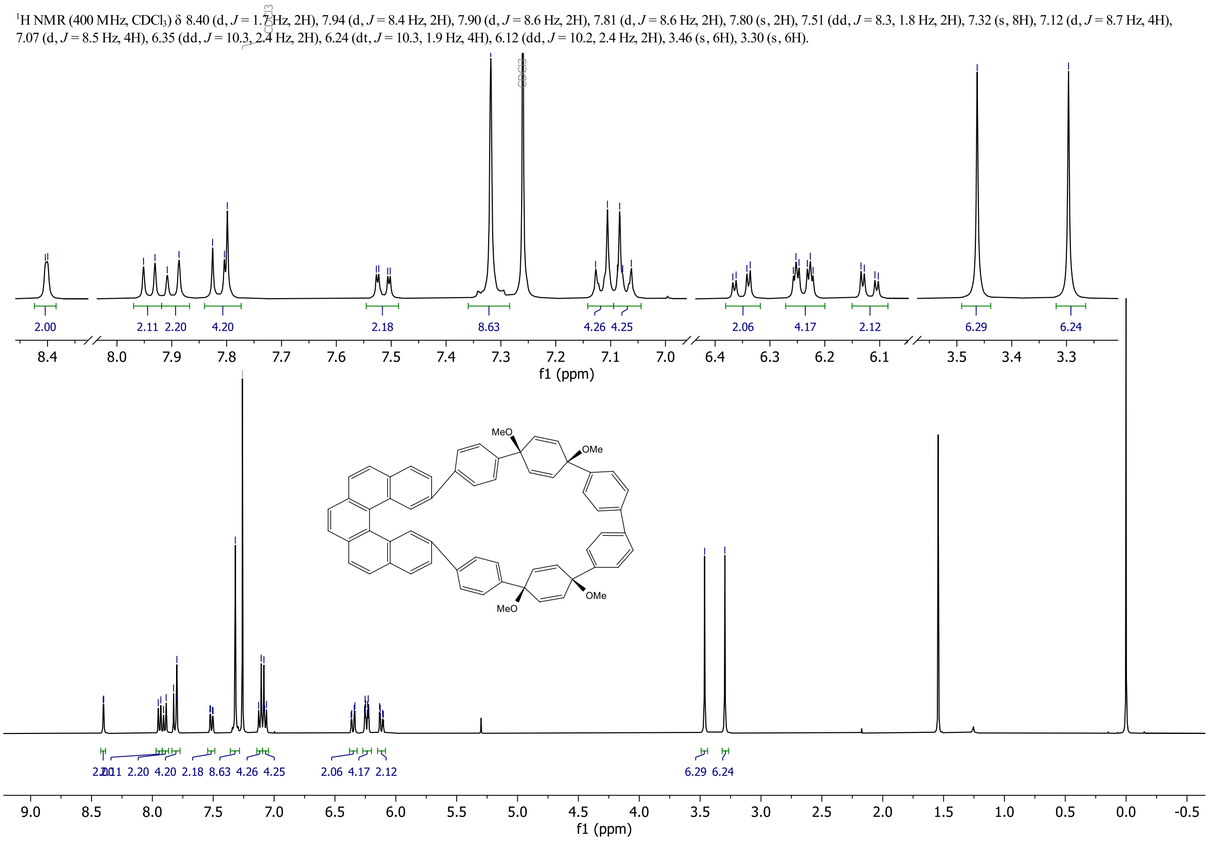

Figure S12. ^{1}H-NMR (500 MHz, $CDCl_3$, 298 K) spectrum of ***pro*-[5,6]HPP**.

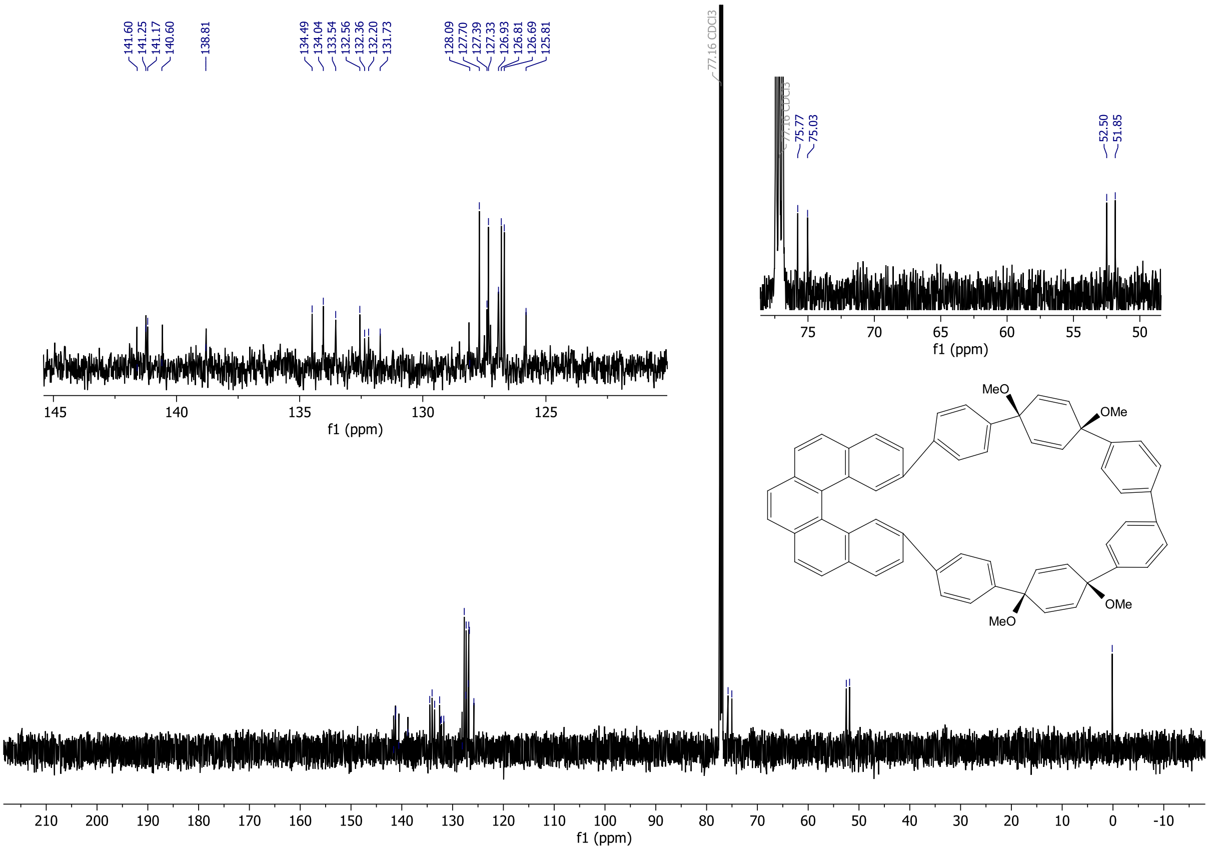

Figure S13. $^{13}C\{^1H\}$-NMR (126 MHz, $CDCl_3$, 298 K) spectrum of ***pro*-[5,6]HPP**. Compound ***pro*-[5,6]HPP** decomposed during the measurement (see 2D NMR for additional characterization).

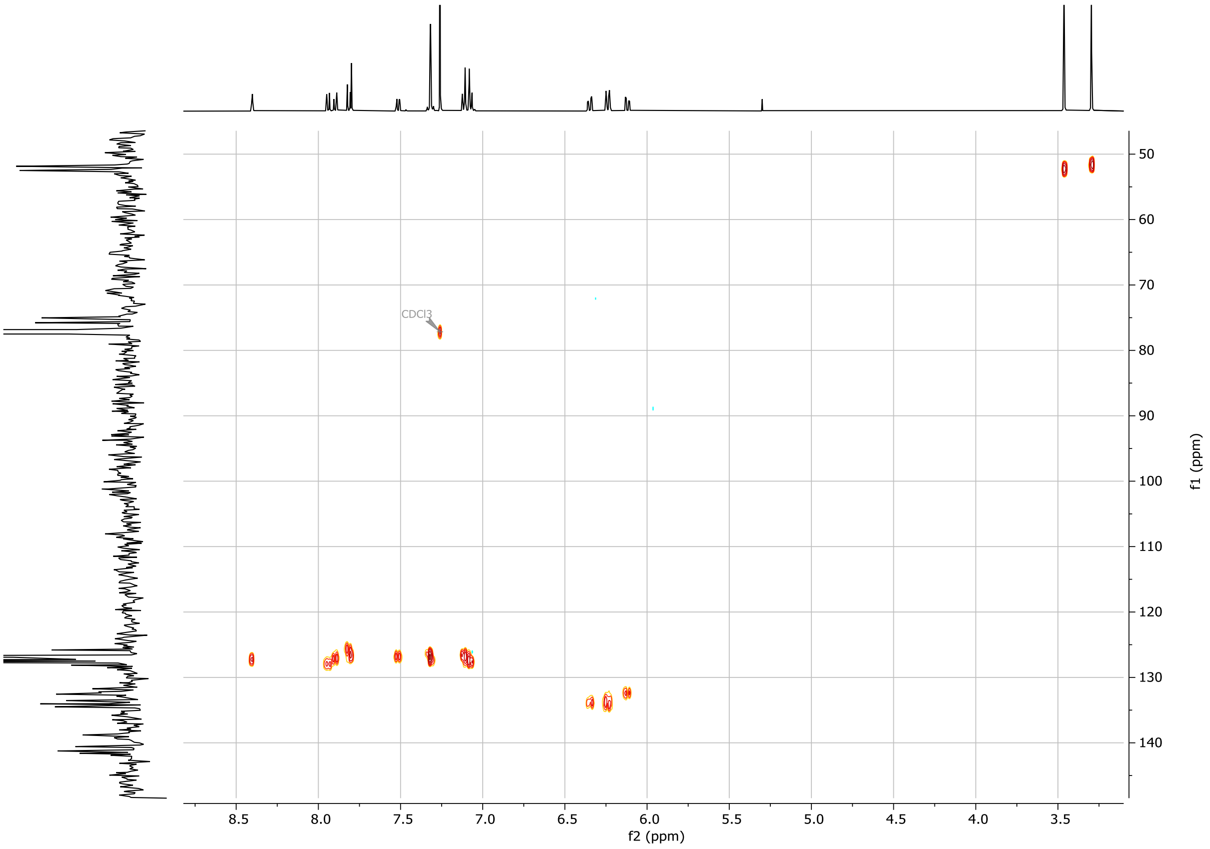

Figure S14. ^1H–^{13}C HSQC spectrum of compound ***pro*-[5,6]HPP** (11.7 T, $CDCl_3$, 298 K).

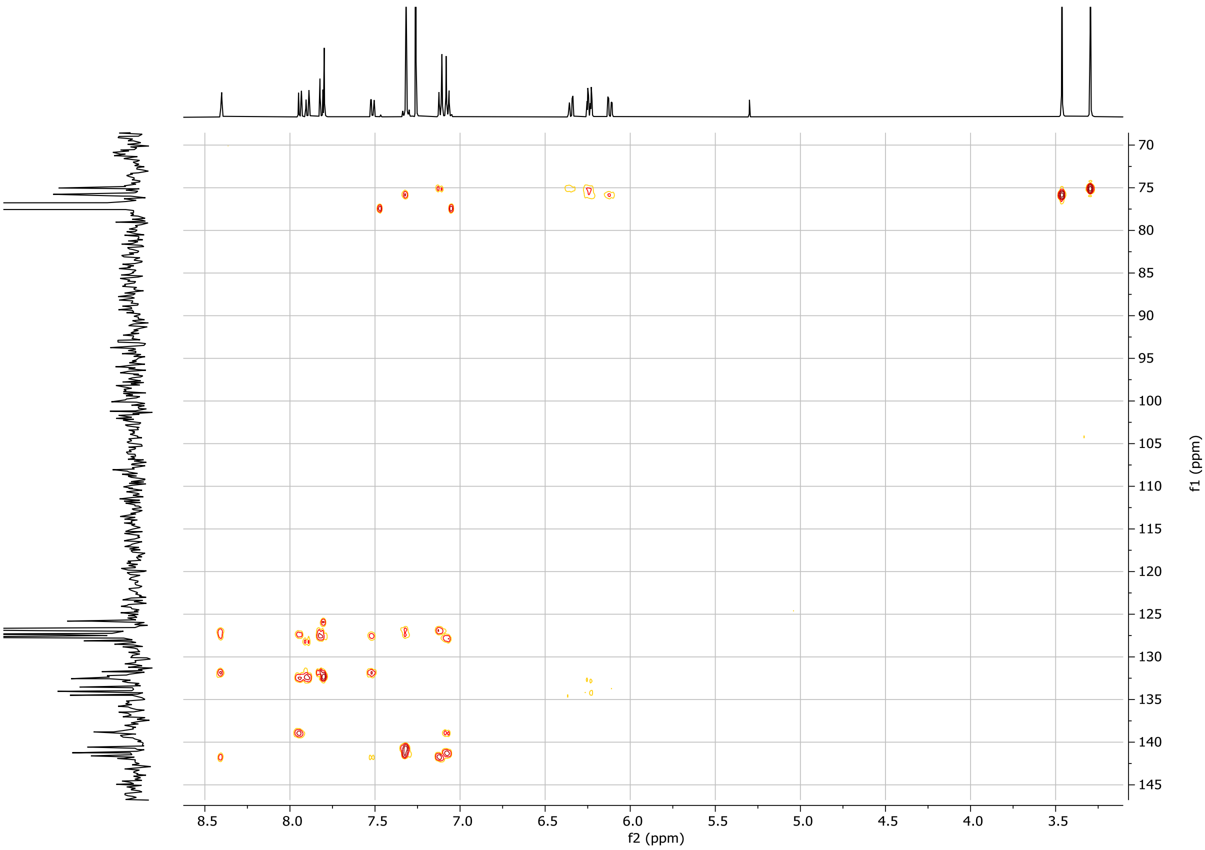

Figure S15. ^{1}H–^{13}C HMBC spectrum of compound ***pro*-[5,6]HPP** (11.7 T, $CDCl_3$, 298 K).

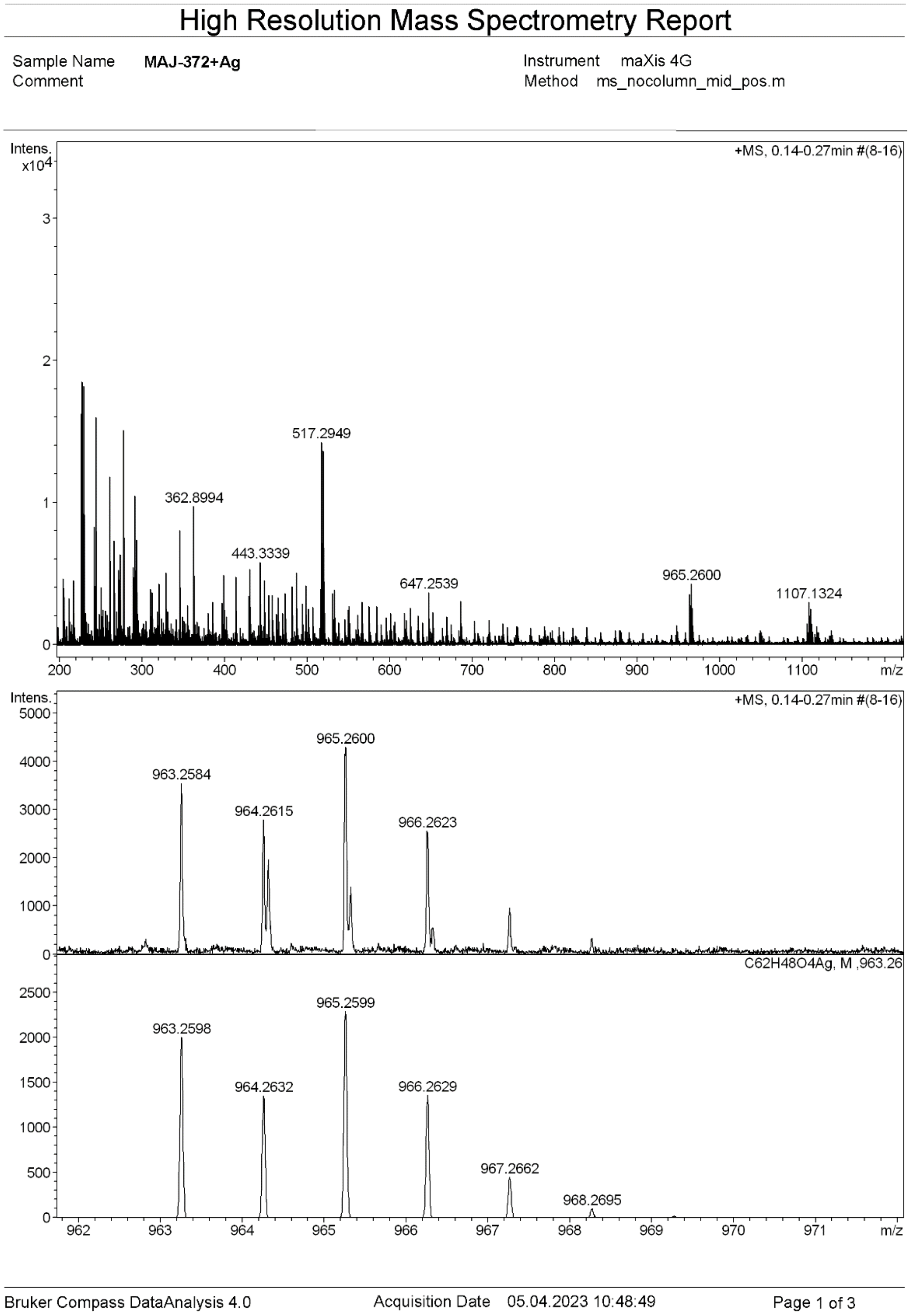

Figure S16. HR-MS (ESI, +) spectrum of ***pro*-[5,6]HPP** $[M+Ag]^+$.

^{1}H NMR (400 MHz, CD_2Cl_2) δ 8.82 (d, J = 1.7 Hz, 2H), 8.01 (d, J = 8.4 Hz, 2H), 7.91 (d, J = 8.5 Hz, 2H), 7.85 – 7.79 (m, 4H), 7.59 (dd, J = 8.4, 1.8 Hz, 2H), 7.54 (dd, J = 9.0, 2.2 Hz, 2H), 7.48 (dd, J = 9.0, 2.1 Hz, 2H), 7.46 – 7.35 (m, 12H), 7.29 (d, J = 8.6 Hz, 4H), 7.24 (d, J = 8.5 Hz, 4H).

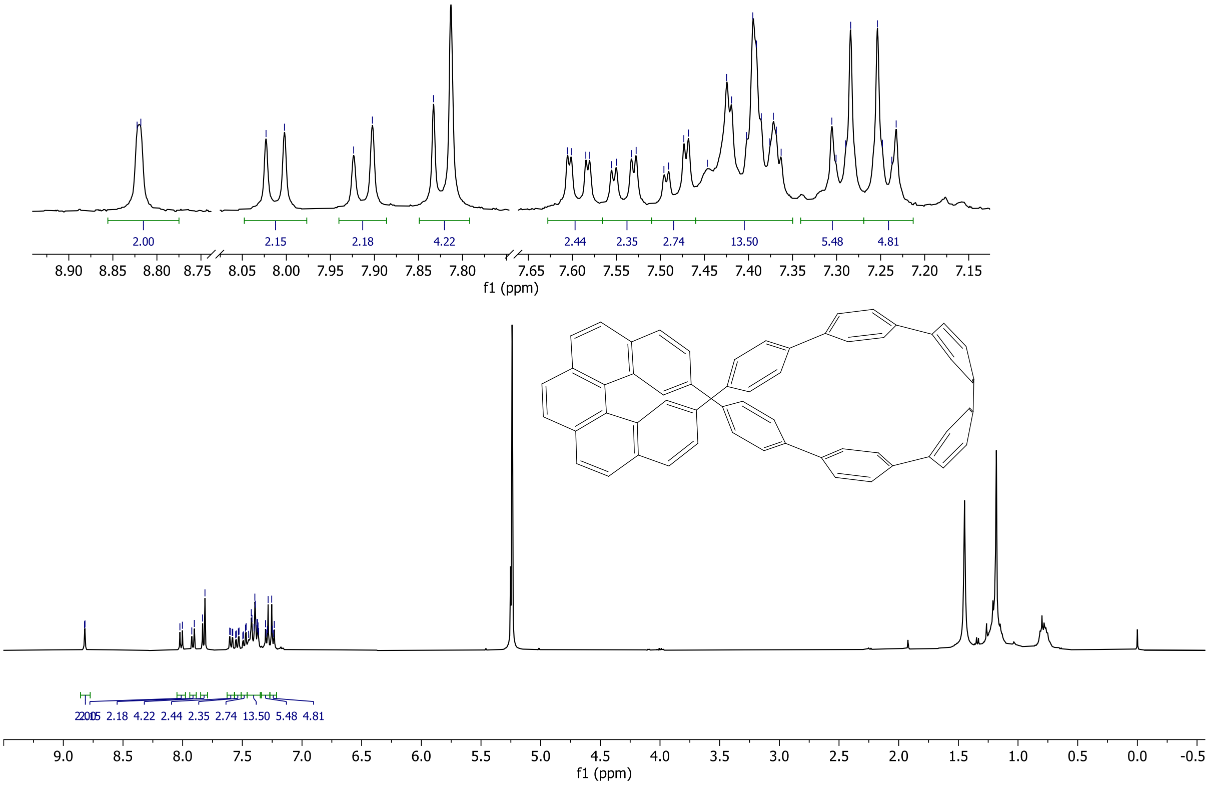

Figure S17. ^{1}H-NMR (400 MHz, CD_2Cl_2, 298 K) spectrum of **[5,6]HPP**.

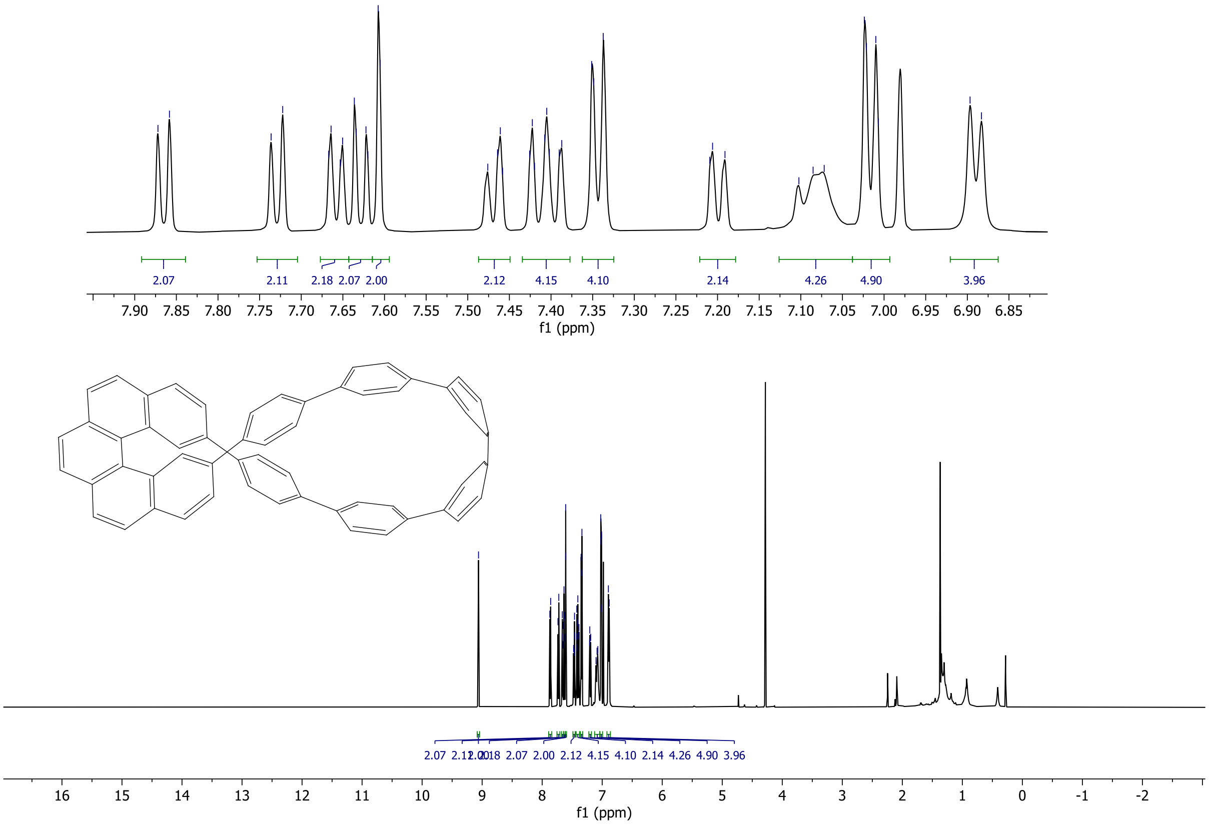

Figure S18. ^{1}H-NMR (600 MHz, toluene-d_8, 298 K) spectrum of **[5,6]HPP**.

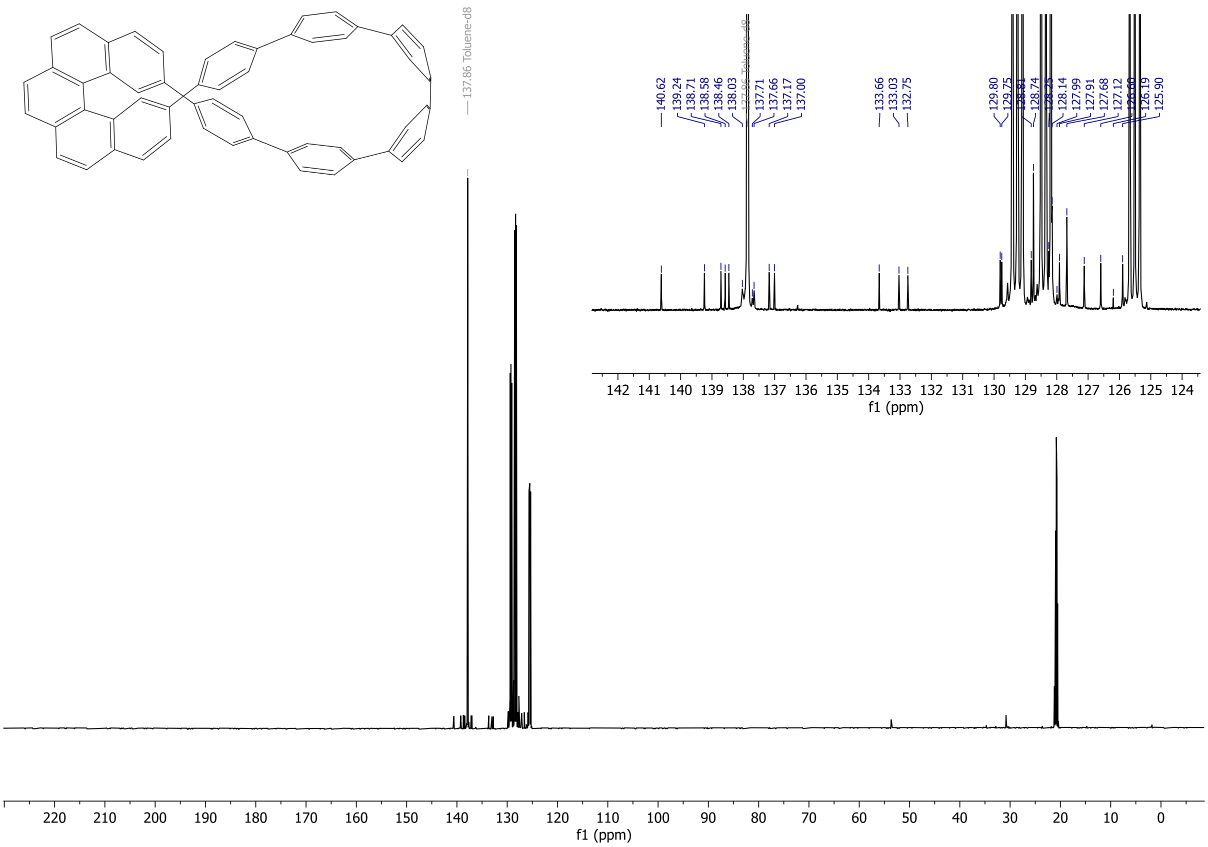

Figure S19. $^{13}C\{^{1}H\}$-NMR (151 MHz, toluene-d_8, 298 K) spectrum of **[5,6]HPP**.

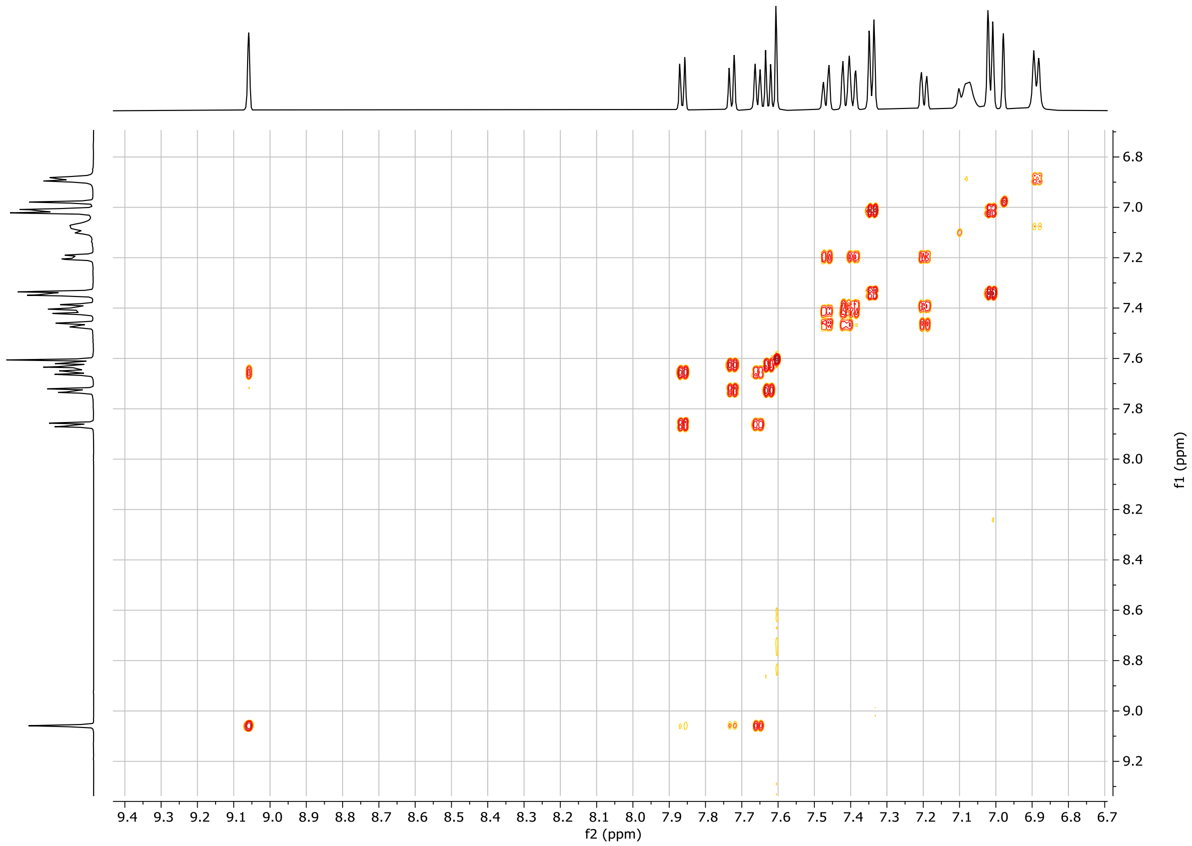

Figure S20. ^{1}H–^{1}H COSY spectrum of **[5,6]HPP** (14.1 T, toluene-d_8, 298 K).

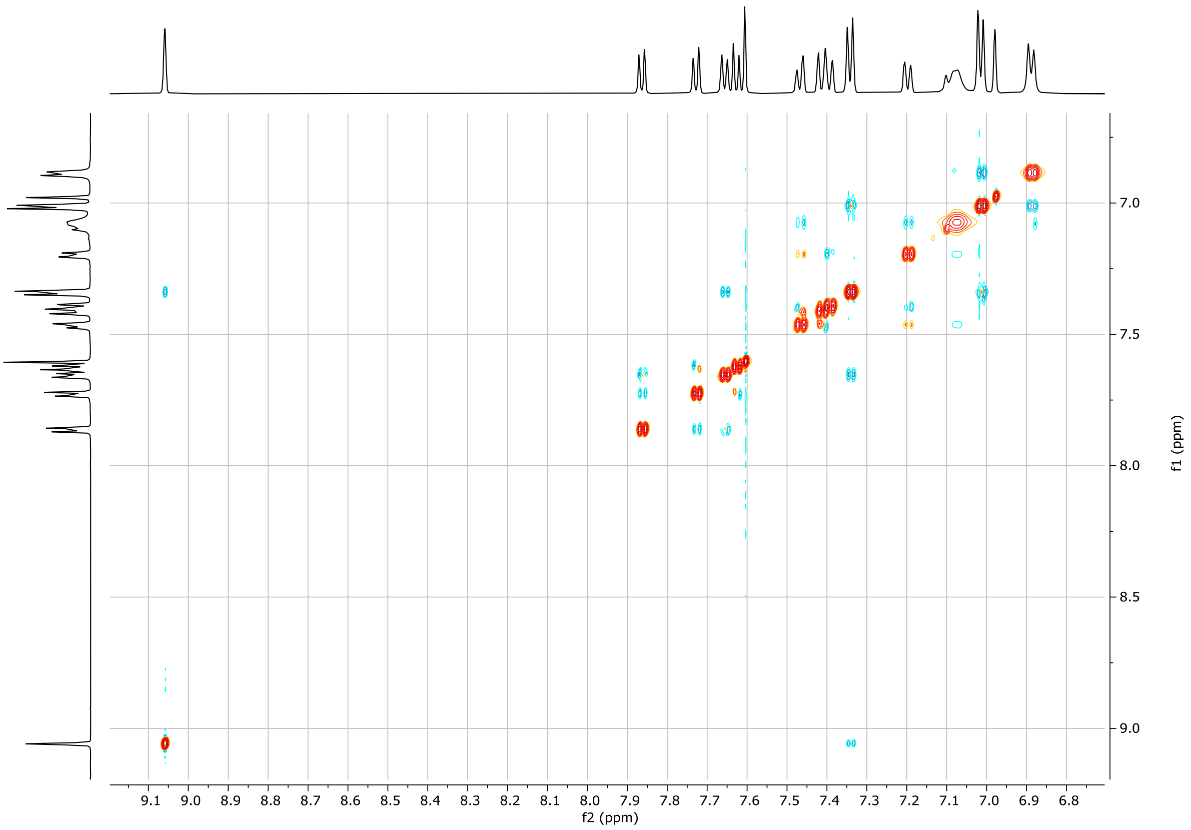

Figure S21. ^{1}H–^{1}H NOESY spectrum of **[5,6]HPP** (14.1 T, toluene-d_8, 298 K).

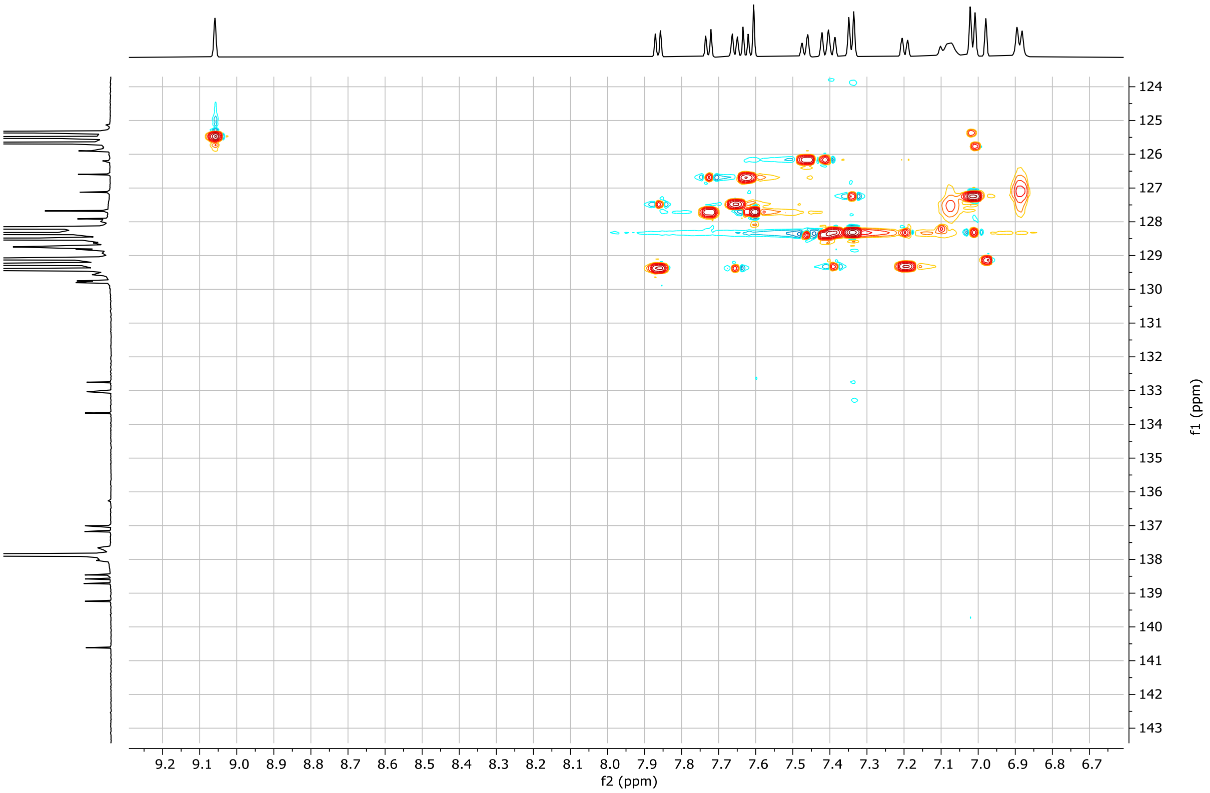

Figure S22. ^{1}H–^{13}C HSQC spectrum of **[5,6]HPP** (14.1 T, toluene-d_8, 298 K).

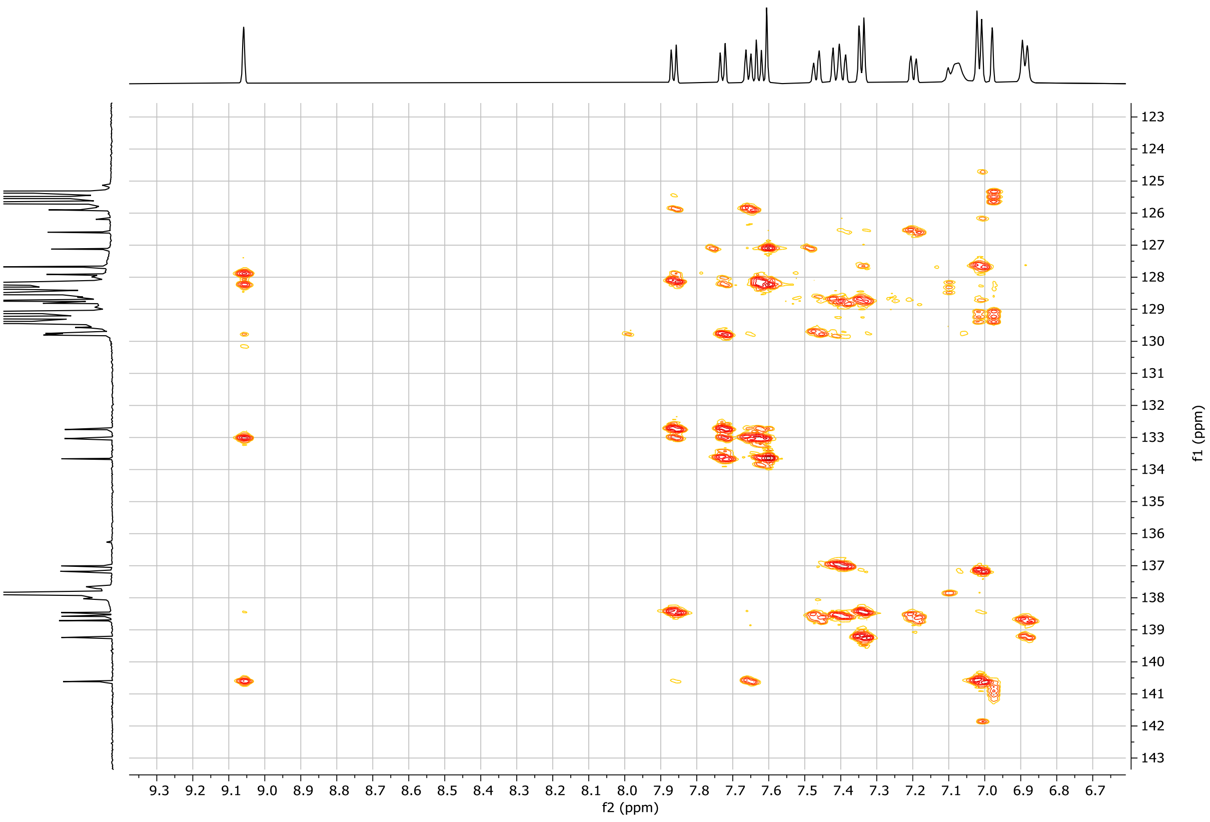

Figure S23. ^{1}H–^{13}C HMBC spectrum of **[5,6]HPP** (14.1 T, toluene-d_8, 298 K).

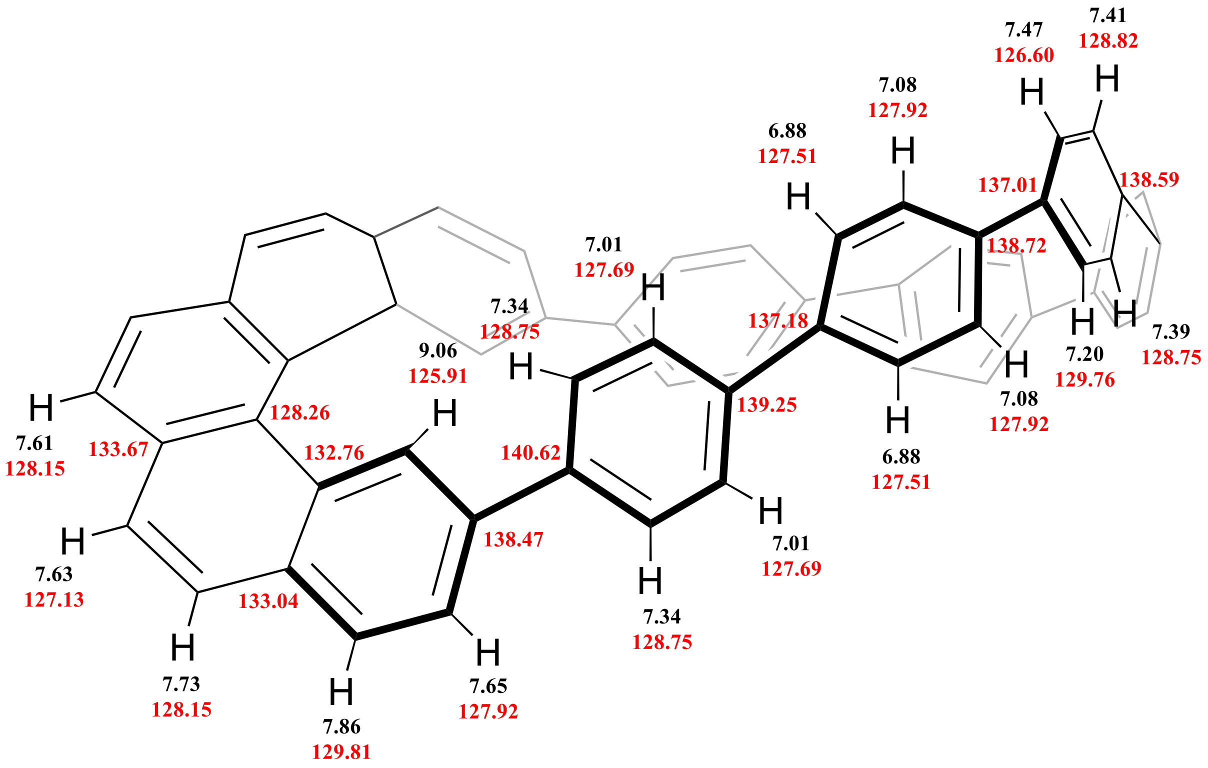

Figure S24. Complete assignment of **[5,6]HPP** ^{1}H and ^{13}C-NMR shifts.

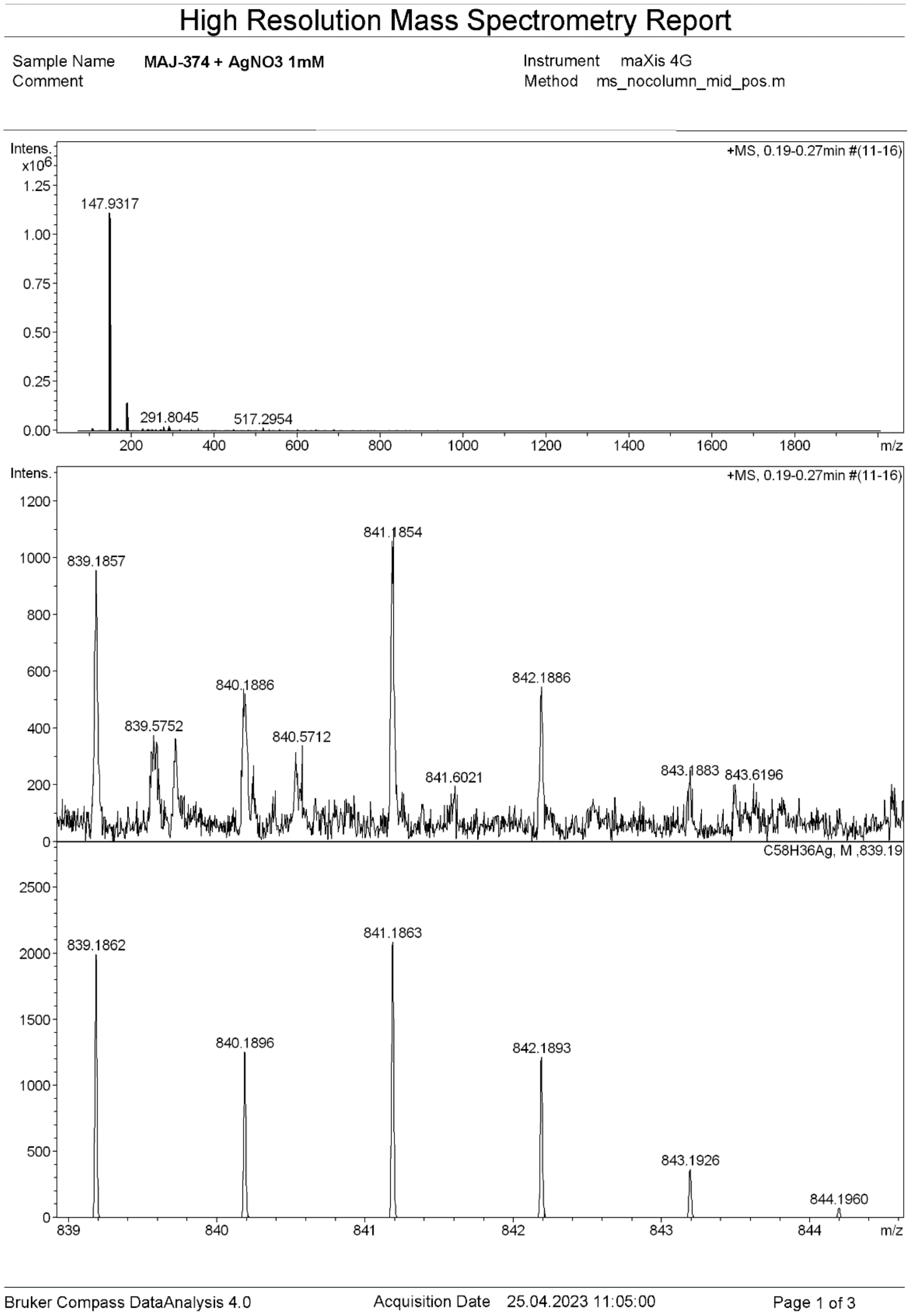

Figure S25. HR-MS (ESI, +) spectrum of **[5,6]HPP** $[M+Ag]^{+}$.

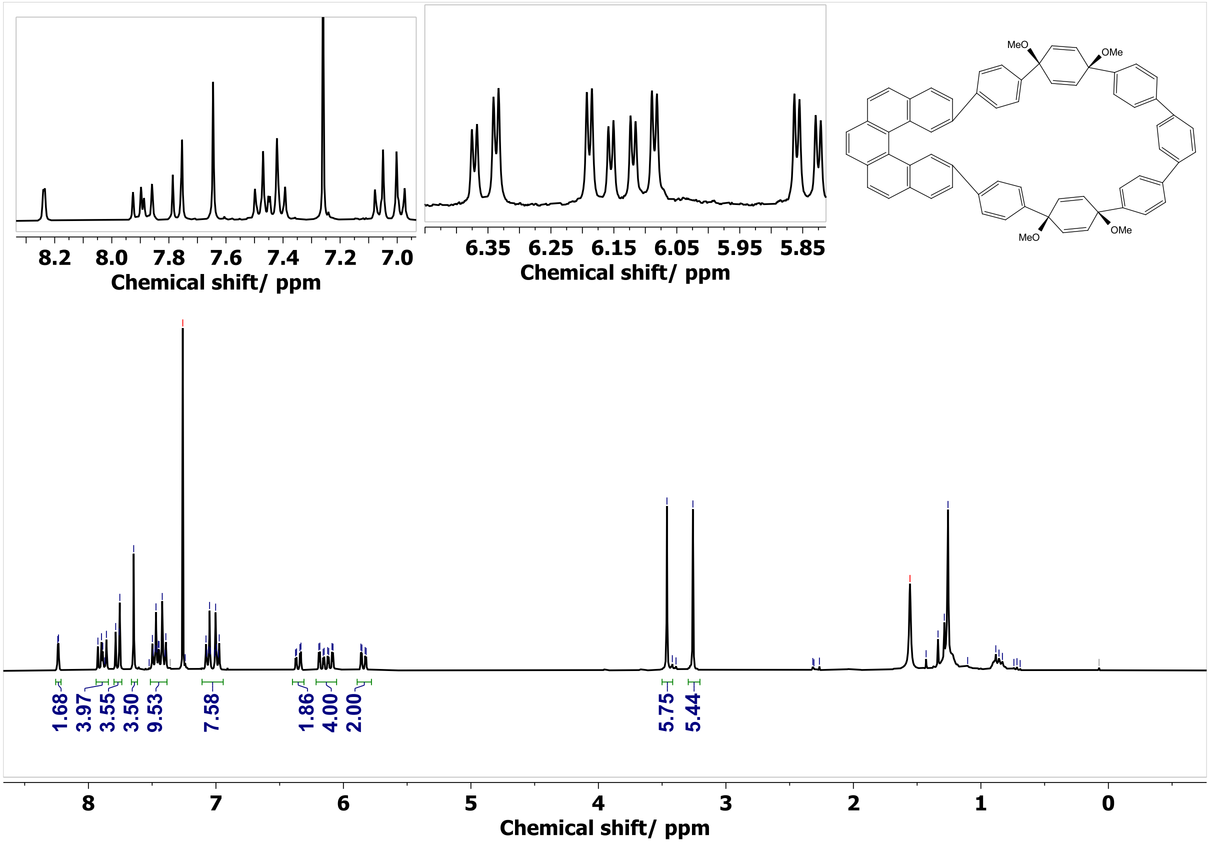

Figure S26. ^{1}H-NMR (300 MHz, $CDCl_3$, 298 K) spectrum of ***pro*-[5,7]HPP**.

^{1}H NMR (500 MHz, $CDCl_3$) δ 8.97 (s, 2H), 8.07 (d, J = 8.4 Hz, 2H), 7.97 (d, J = 8.5 Hz, 2H), 7.91 – 7.85 (m, 4H), 7.70 (dd, J = 8.4, 1.8 Hz, 2H), 7.56 (dd, J = 9.0, 2.1 Hz, 2H), 7.52 (d, J = 8.1 Hz, 4H), 7.49 – 7.46 (m, 4H), 7.46 – 7.42 (m, 10H), 7.41 – 7.34 (m, 8H).

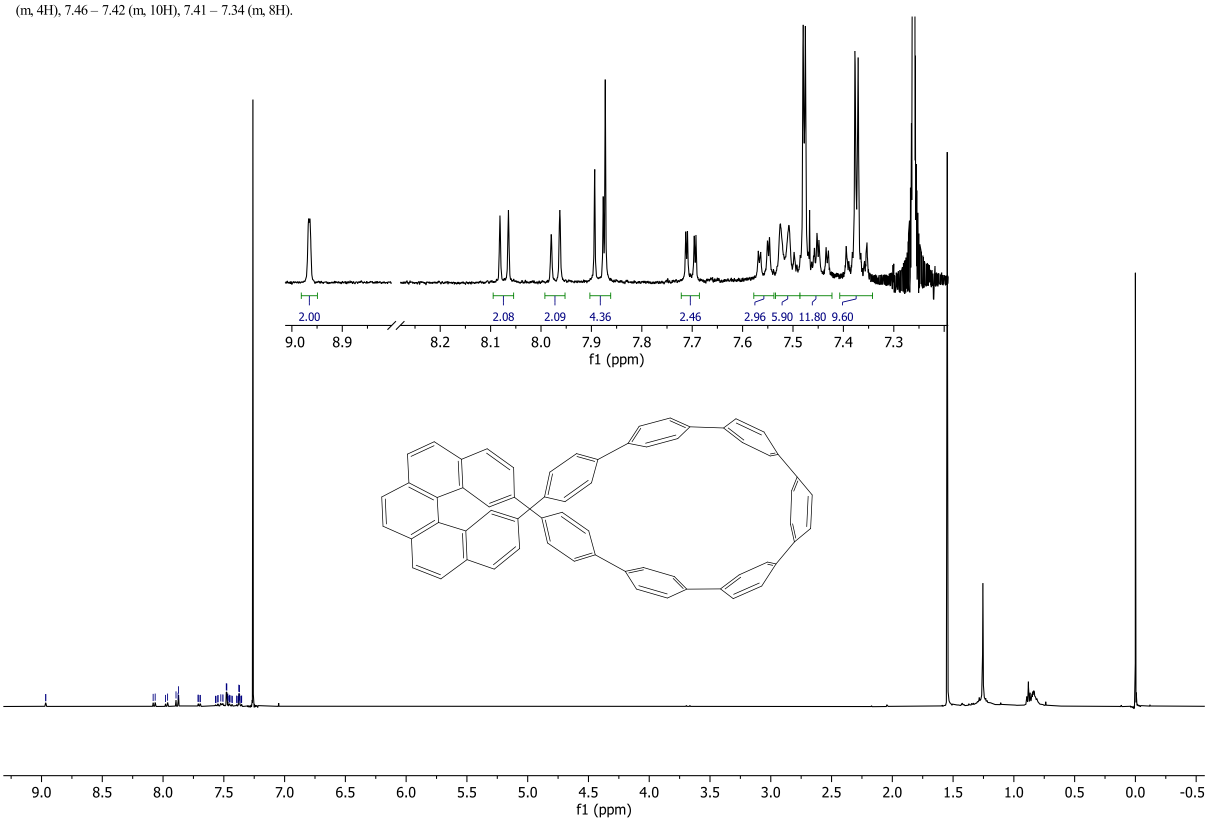

Figure S27. ^{1}H-NMR (500 MHz, $CDCl_3$, 298 K) spectrum of **[5,7]HPP**.

BSOL001928 Juraj Malincík/ - MAJ-357 - DCM - DCTB 1:10

ETH Eidgenössische Technische Hochschule Zürich
Swiss Federal Institute of Technology Zurich

Acquisition Parameter

Method:	MALDI_MS_POS_300-3000_2M_16AvScans			Acquisition Date:	14.12.2022 10:38:20
File Name:	D:\Data\ETH Data\BSOL0019xx\BSOL001928_0_C15_000001.d			Operator:	Louis Bertschi
Source	Dual (MALDI/ESI)	Polarity	Positive	Nebulizer Gas	1.0 bar
Broadband Low Mass	303.1 m/z			Drying Gas Flow Rate	4.0 L/min
Broadband High Mass	3000.0 m/z	Laser Power	13.6 lp	Capillary	3000.0 V
No. of Cell Fills	1			Drying Gas Temperature	200.0 °C
Apodization	Full-Sine	Time of Flight to Detector	0.001 sec		

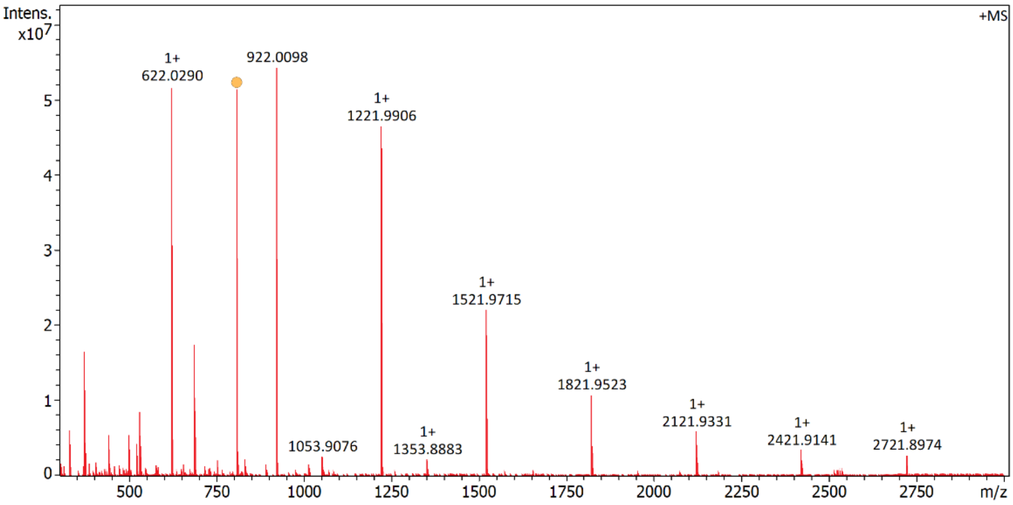

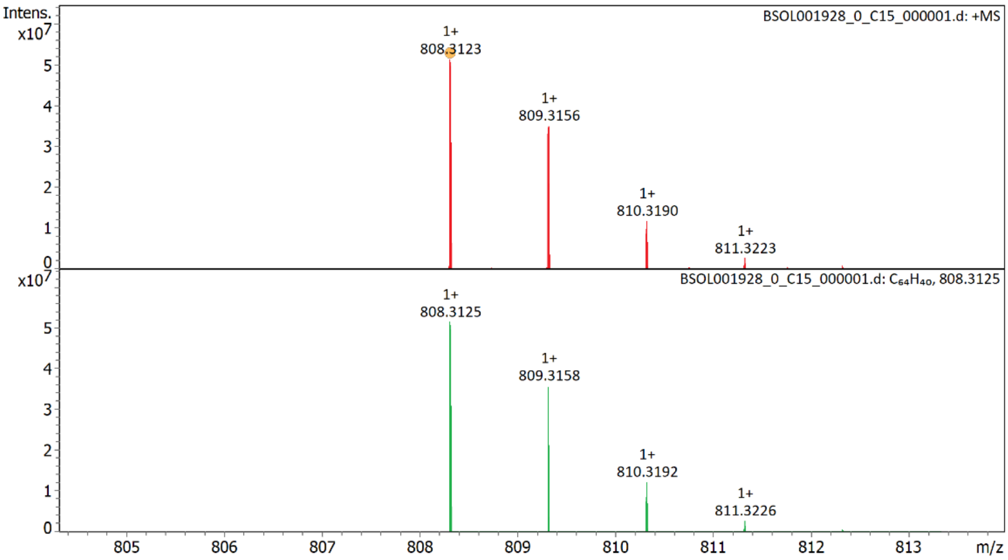

Figure S28. HR-MS (MALDI, DCTB matrix, +) spectrum of **[5,7]HPP** $[M]^+$.

Preparative HPLC

5 mg of a racemic **[5,5]HPP** was dissolved in 5 mL of DCM/heptane = 1/1 mixture, filtered with a syringe filter and injected into the HPLC equipped with Chiralpak IG (20×250) mm column by Daicel Chemical Industries Ltd. DCM/heptane = 1/1 was used as eluent at 10 mL min^{-1} flow rate.

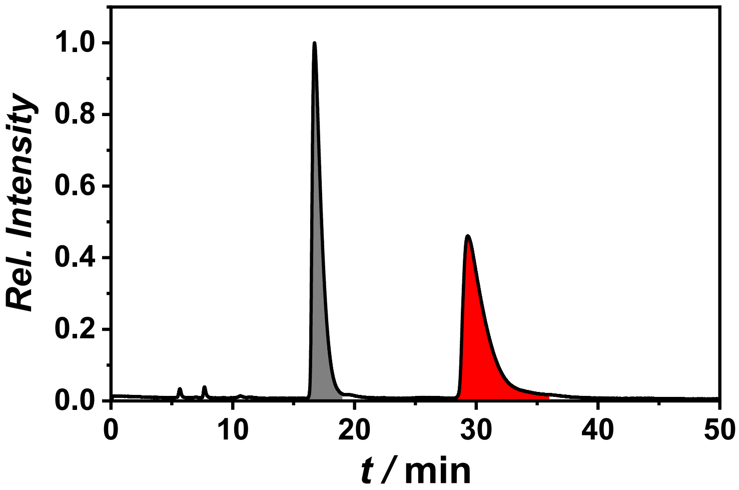

Figure S29. Preparative HPLC chromatogram of **[5,5]HPP** with (*P*)-enantiomers (grey) eluting first followed by (*M*)-enantiomers (red).

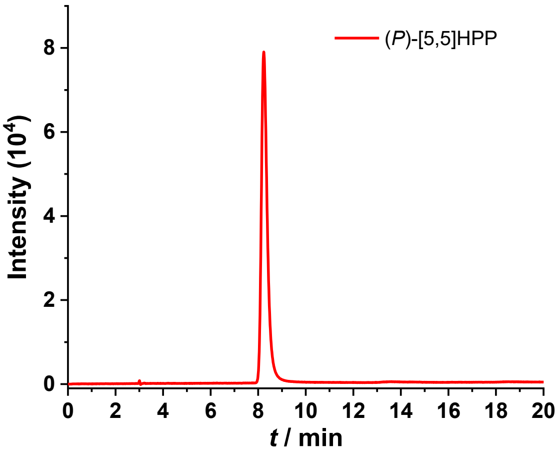

Figure S30. HPLC chromatogram of **(*P*)-[5,5]HPP** in 1:1 DCM/heptane mixture (flow rate 1 ml/min) used for racemization experiments.

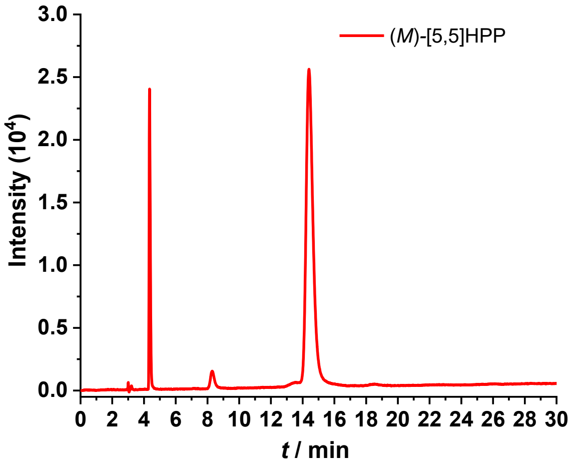

Figure S31. HPLC chromatogram of ***(M)*-[5,5]HPP** in 1:1 DCM/heptane mixture (flow rate 1 ml/min). Due to presence of small amounts of (*P*) enantiomer, this enantiomer was not used for experiments.

10 mg of a racemic **[5,6]HPP** was dissolved in 5 mL of DCM and diluted with 5 mL of heptane. The resulting solution was filtered with a syringe filter and injected into the HPLC equipped with Chiralpak IG, 5 µm, 30×250 mm column by Daicel Chemical Industries Ltd. DCM/heptane = 1/1 was used as eluent at 20 mL min^{-1} flow rate. The chromatogram shown in Figure S33 was recorded one month after the initial chiral HPLC resolution, and the enantiomeric separation remained stable over this period.

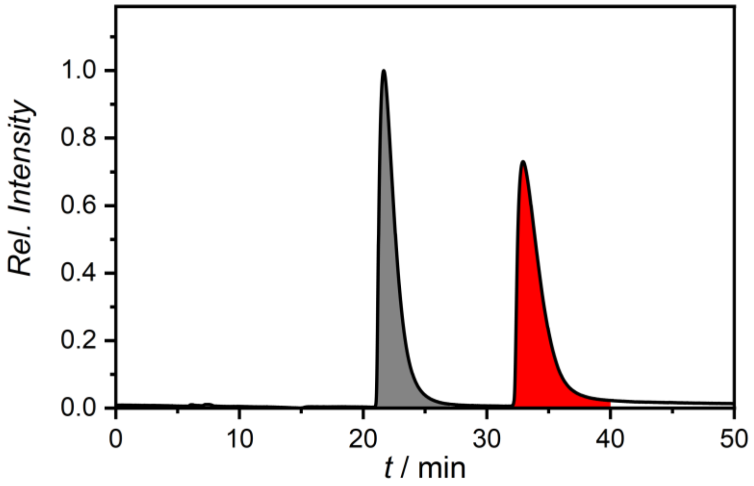

Figure S32. Preparative HPLC chromatogram of **[5,6]HPP** with (*P*)-enantiomers (grey) eluting first followed by (*M*)-enantiomers (red).

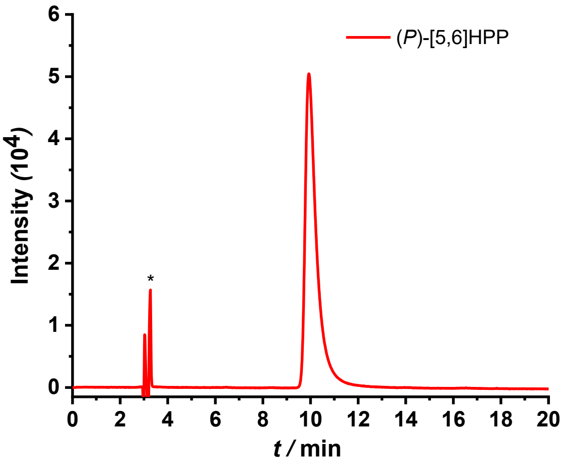

Figure S33. HPLC chromatogram of **(*P*)-[5,6]HPP** in 1:1 DCM/heptane mixture (flow rate 1 ml/min) used for racemization experiments. * = injection peak. Enantiopure **(*M*)-[5,6]HPP** was not obtained, therefore **(*P*)-[5,6]HPP** was used for the racemization kinetics.

4.7 mg of a racemic **[5,7]HPP** was dissolved in 5 mL of DCM/heptane = 1/1 mixture, filtered with a syringe filter and injected into the HPLC equipped with Chiralpak IG, 5 µm, 30×250 mm column by Daicel Chemical Industries Ltd. DCM/heptane = 1/1 was used as eluent at 20 mL min^{-1} flow rate. The chromatograms shown in Figure S35 and S36 were recorded one month after the initial chiral HPLC resolution, and the enantiomeric separation remained stable over this period.

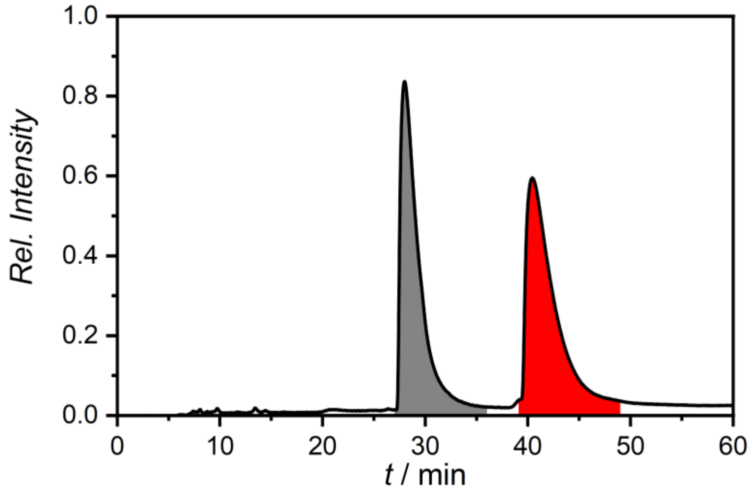

Figure S34. Preparative HPLC chromatogram of **[5,7]HPP** with (*P*)-enantiomers (grey) eluting first followed by (*M*)-enantiomers (red).

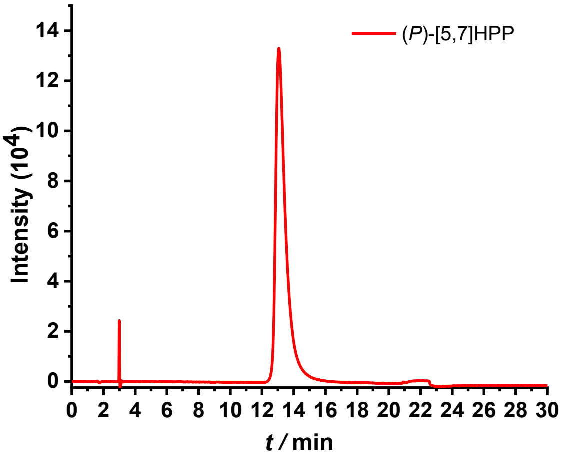

Figure S35. HPLC chromatogram of ***(P)*-[5,7]HPP** in 1:1 DCM/heptane mixture (flow rate 1 ml/min).

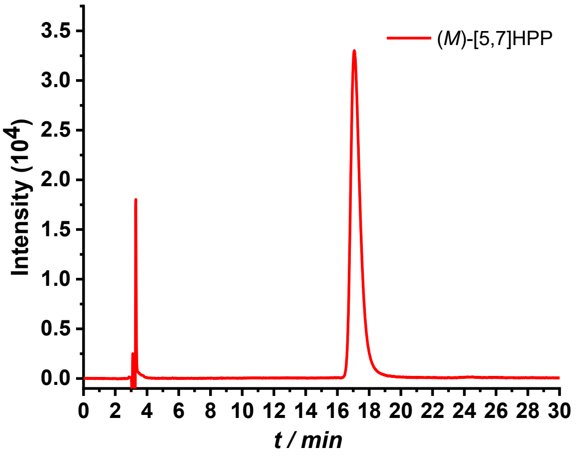

Figure S36. HPLC chromatogram of ***(M)*-[5,7]HPP** in 1:1 DCM/heptane mixture (flow rate 1 ml/min) used for racemization experiments.

Stability of [5,n]HPPs

A sample of [5,5]HPP was prepared by dissolving the nanohoop in deoxygenated toluene. The HPLC chromatogram of this sample was measured and the samples was left to stand overnight in dark at room temperature. After 24 h, the sample was measured again (Figure S37). No decomposition of the sample was observed. During our attempts to grow the single crystals of [5,6]HPP and [5,7]HPP, their deoxygenated solutions were kept at room temperature in the dark for up to a month and no significant decomposition was observed.

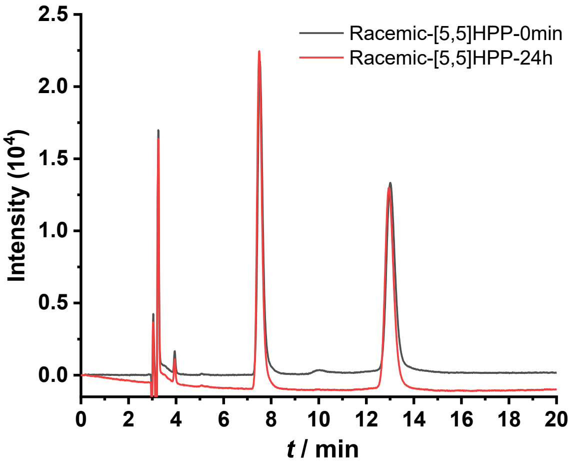

Figure S37. HPLC chromatogram of ***rac*-[5,5]HPP** in deoxygenated toluene monitored for 24 h.

Racemization Kinetics

Extensive racemization kinetics experiments were performed for the [5,n]HPP series. At each studied temperature, three independent sample stock solutions were prepared in three vials by dissolving 1 mg of enantiopure nanohoop each in 1 ml toluene and the samples were degassed for 30 min. All three sample vials were then placed on a heated plate at the temperature of interest (the temperature deviation for the experimental determination of racemization was no more than ±0.5 °C) and samples were collected over time from each vial at each time point, cooled down to room temperature and injected into the analytical HPLC equipped with a chiralpak IG column (4.6×250) using DCM-n-heptane (1:1) as the eluent and 1 ml min^{-1} flow rate. Kinetics experiments were performed at temperatures ranges of 50–65, 75–90, and 85–100 °C for **[5,5]HPP**, **[5,6]HPP** and **[5,7]HPP**, respectively. Enantiomeric excess (ee) was calculated using the ratios of areas under the peaks in the HPLC chromatograms. The enantiomeric excess was plotted against time (*t*) to calculate the rate constants of racemization (k_{rac}) which gave access to the rate constants of enantiomerization (k_{en}).[1] Further, the activation barriers of racemization ($\Delta G^{\neq}$) were calculated for all the nanohoops at different temperatures. Using the Eyring equation, other thermodynamic parameters such as the activation enthalpy ($\Delta H^{\neq}$) and entropy ($\Delta S^{\neq}$) were also extracted. The rate constants k_{rac}, k_{en}, half-life, $\Delta G^{\neq}$, $\Delta H^{\neq}$ and $\Delta S^{\neq}$ were determined using the following equations:

$$ee(t) = ee_0 \cdot e^{-k_{rac}t} \quad (1)$$

$$k_{en} = \frac{k_{rac}}{2} \quad (2)$$

$$\tau = \frac{\ln 2}{k_{en}} \quad (3)$$

$$\ln\frac{k_{en}}{T} = -\frac{\Delta H^{\neq}}{R}\frac{1}{T} + \ln\frac{k_B}{h} + \frac{\Delta S^{\neq}}{R} \quad (4)$$

$$\Delta G^{\neq} = \Delta H^{\neq} - T\Delta S^{\neq} \quad (5)$$

*In tables S2, S3, S6, S8, S9 and S11, the missing data points are due to instrumental error.

Table S1. First order decay data (ee vs time) for **[5,5]HPP** at 50ºC at different time scale.

Time (min)	ee (50ºC) batch 1	ee (50ºC) batch 2
0	87.49	87.49
60	68.60	68.72
120	54.26	54.10
180	43.07	42.54
240	33.55	32.61
300	29.08	25.27

Table S2. First order decay data (ee vs time) for **[5,5]HPP** at 55ºC at different time scale.

Time (min)	ee (55ºC) batch 1	ee (55ºC) batch 2	ee (55ºC) batch 3
0	90.87	90.87	90.87
60	59.62	60.72	-*
120	38.13	38.17	36.57
180	24.27	24.00	24.08
240	14.97	15.01	14.56
300	8.82	9.00	9.02
360	5.60	4.92	5.82

Table S3. First order decay data (ee vs time) for **[5,5]HPP** at 60ºC at different time scale.

Time (min)	ee (60ºC) batch 1	ee (60ºC) batch 2	ee (60ºC) batch 3
0	93.98	93.98	93.98
30	61.63	58.84	60.54
60	40.82	38.39	39.67
90	26.97	25.78	26.00
120	18.34	16.46	17.14
150	12.38	-	11.68

Table S4. First order decay data (ee vs time) for **[5,5]HPP** at 65ºC at different time scale.

Time (min)	ee (65ºC) batch 1	ee (65ºC) batch 2	ee (65ºC) batch 3
0	95.65	95.65	95.65
30	42.30	40.48	41.27
60	20.78	18.77	18.99
90	8.73	8.43	8.70
120	3.88	4.32	4.04
150	1.14	1.80	1.84

Table S5. First order decay data (ee vs time) for **[5,6]HPP** at 75ºC at different time scale.

Time (min)	ee (75ºC) batch 1	ee (75ºC) batch 2	ee (75ºC) batch 3
0	>99	>99	>99
60	86.17	86.58	85.14
120	76.63	75.94	75.09
180	67.90	68.04	66.26
240	60.21	59.22	58.83
300	52.95	52.98	52.52
360	47.21	47.53	46.33
480	37.10	36.68	37.06

Table S6. First order decay data (ee vs time) for **[5,6]HPP** at 80°C.

Time (min)	ee (80°C) batch 1	ee (80°C) batch 2	ee (80°C) batch 3
0	>99	>99	>99
60	78.86	77.18	77.78
120	63.06	65.22	63.91
180	52.17	52.84	52.15
240	-	42.77	42.64
300	34.80	34.87	34.69
360	28.18	28.51	26.65
480	21.42	22.71	21.67

Table S7. First order decay data (ee vs time) for **[5,6]HPP** at 85°C.

Time (min)	ee (85°C) batch 1	ee (85°C) batch 2	ee (85°C) batch 3
0	>99	>99	>99
30	76.67	75.71	73.92
60	62.21	60.95	60.57
90	48.77	48.09	47.43
120	38.18	37.43	37.39
150	30.21	29.64	29.97
180	23.92	23.78	23.76
240	14.83	15.16	14.92

Table S8. First order decay data (ee vs time) for **[5,6]HPP** at 90°C.

Time (min)	ee (90°C) batch 1	ee (90°C) batch 2	ee (90°C) batch 3
0	>99	>99	>99
60	36.75	33.53	34.50
90	21.94	20.97	21.23
120	12.26	11.20	11.79
135	-	-	8.70
150	7.28	5.48	5.86

Table S9. First order decay data (ee vs time) for **[5,7]HPP** at 85°C.

Time (min)	ee (85°C) batch 1	ee (85°C) batch 2	ee (85°C) batch 3
0	>99	>99	>99
60	82.72	81.40	81.75
120	-	69.81	69.45
180	59.60	59.14	-
240	51.14	50.38	50.34
300	43.03	42.47	43.42
360	37.05	35.61	35.28
420	29.75	28.56	28.54

Table S10. First order decay data (ee vs time) for **[5,7]HPP** at 90°C.

Time (min)	ee (90°C) batch 1	ee (90°C) batch 2	ee (90°C) batch 3
0	>99	>99	>99
60	62.72	61.56	63.71
120	39.90	40.78	40.91
180	26.37	27.55	27.81
240	18.06	19.07	18.90
300	13.10	13.50	15.02
360	10.80	9.71	10.74

Table S11. First order decay data (ee vs time) for **[5,7]HPP** at 95°C.

Time (min)	ee (95°C) batch 1	ee (95°C) batch 2	ee (95°C) batch 3
0	>99	>99	>99
30	70.43	-	69.46
60	51.13	54.85	50.95
90	37.53	37.82	37.01
150	20.43	20.58	20.08
180	15.66	15.27	16.05
240	-	10.23	11.16

Table S12. First order decay data (ee vs time) for **[5,7]HPP** at 100°C.

Time (min)	ee (100°C) batch 1	ee (100°C) batch 2	ee (100°C) batch 3
0	>99	>99	>99
15	66.42	66.76	66.44
30	49.68	50.40	50.25
45	36.70	36.65	33.22
60	27.25	27.13	28.02
75	19.98	20.31	17.92
90	14.71	15.10	14.63
120	9.36	9.19	8.93

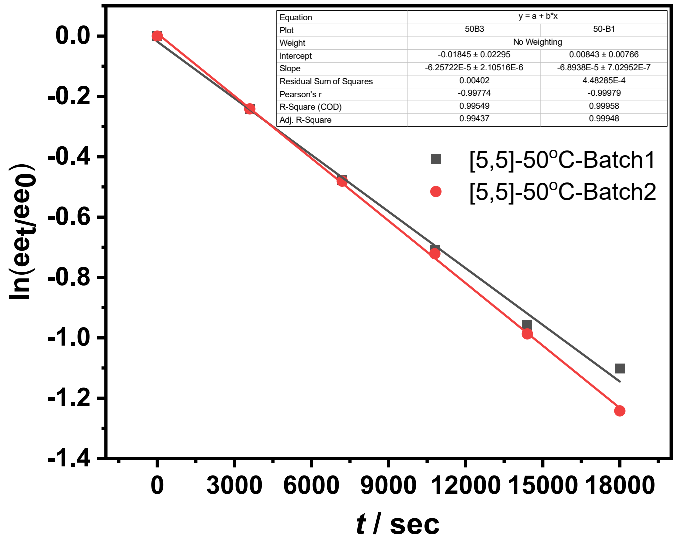

Figure S38. First order decay plots from three independent measurements for **[5,5]HPP** at 50°C.

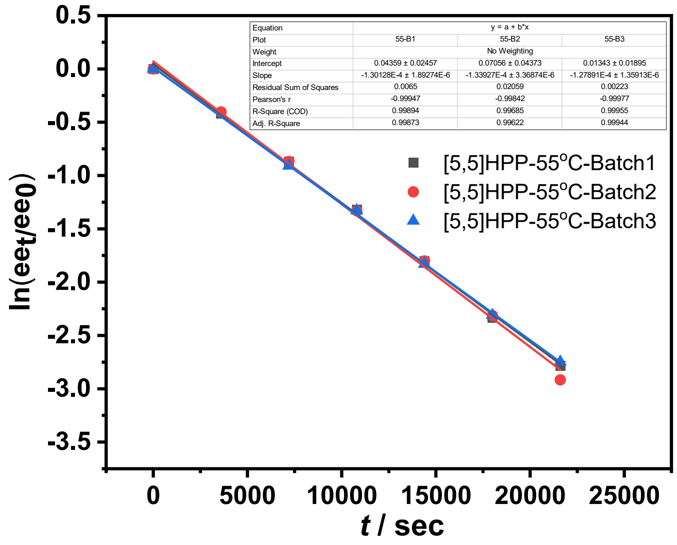

Figure S39. First order decay plots from three independent measurements for **[5,5]HPP** at 55°C.

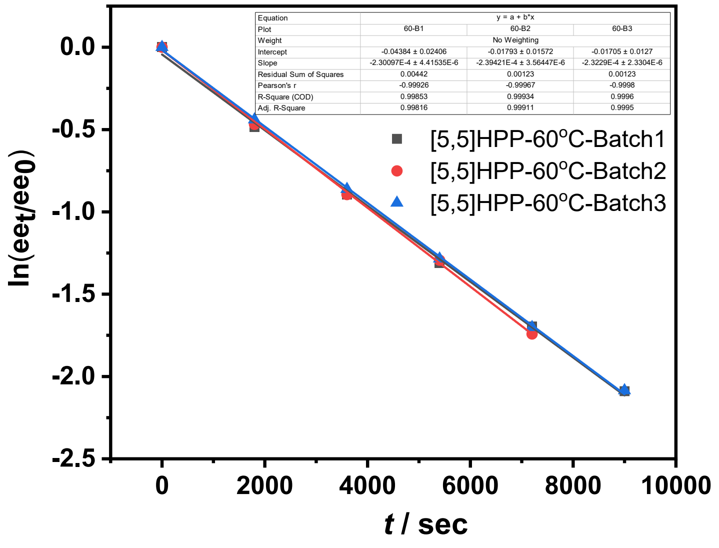

Figure S40. First order decay plots from three independent measurements for **[5,5]HPP** at 60°C.

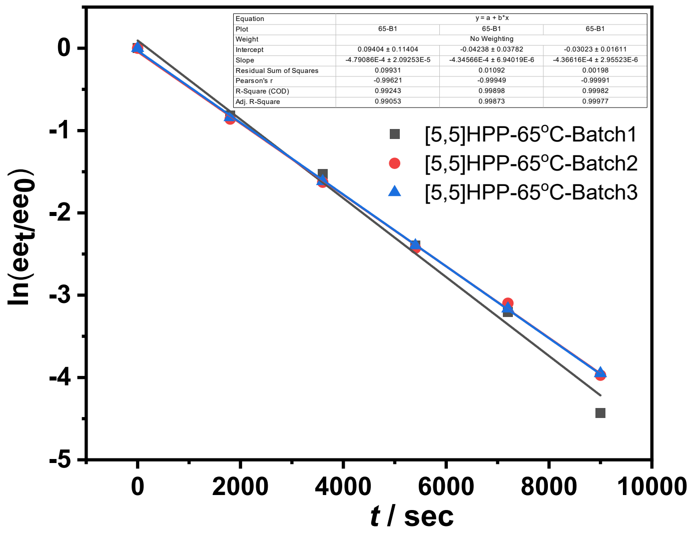

Figure S41. First order decay plots from three independent measurements for **[5,5]HPP** at 65°C.

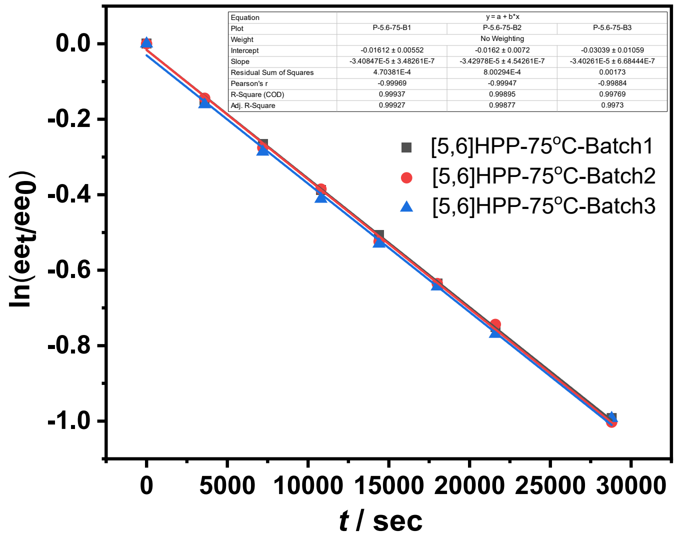

Figure S42. First order decay plot from three independent measurements for **[5,6]HPP** at 75°C.

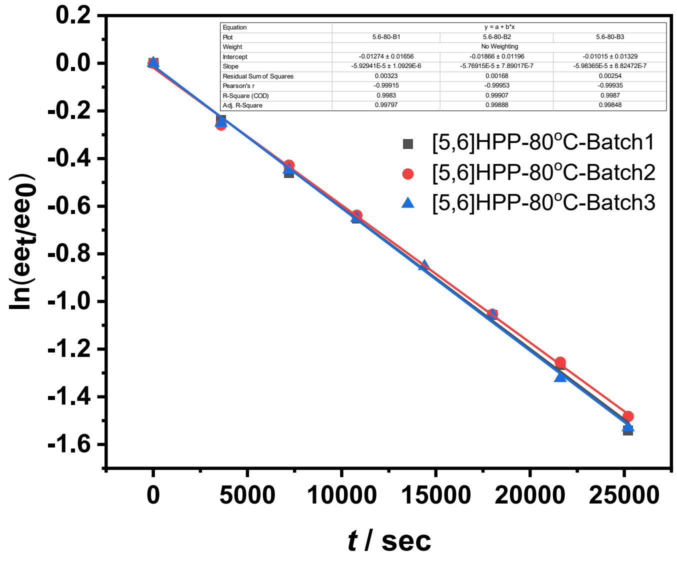

Figure S43. First order decay plots from three independent measurements for **[5,6]HPP** at 80°C.

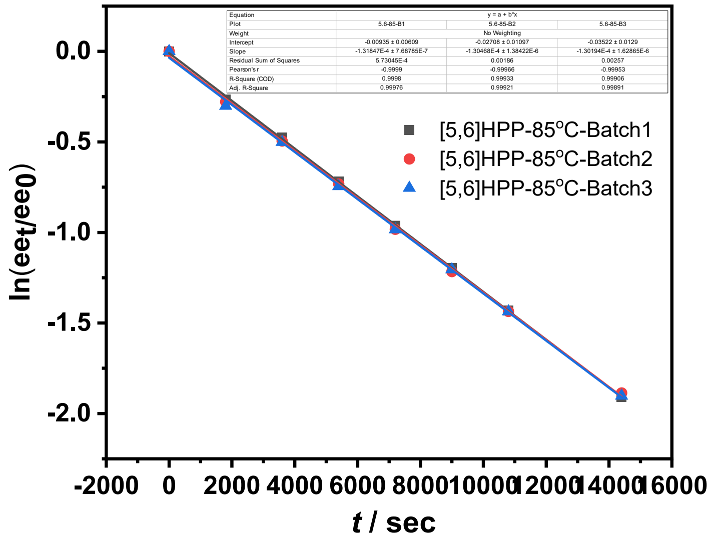

Figure S44. First order decay plots from three independent measurements for **[5,6]HPP** at 85°C.

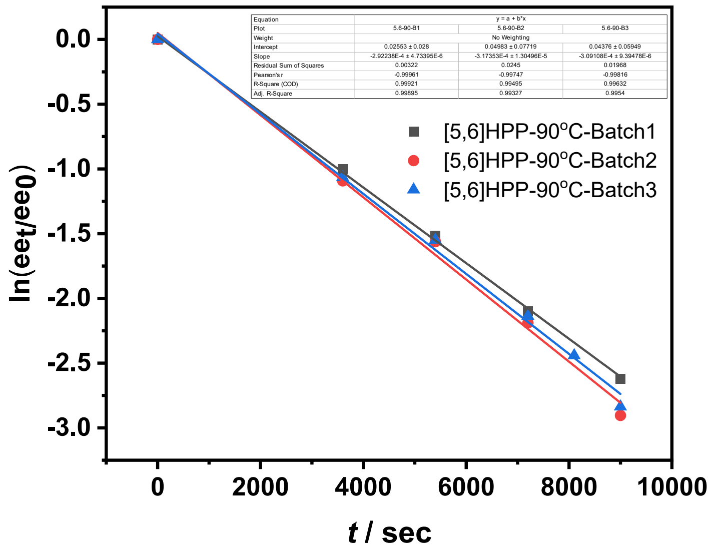

Figure S45. First order decay plots from three independent measurements for **[5,6]HPP** at 90°C.

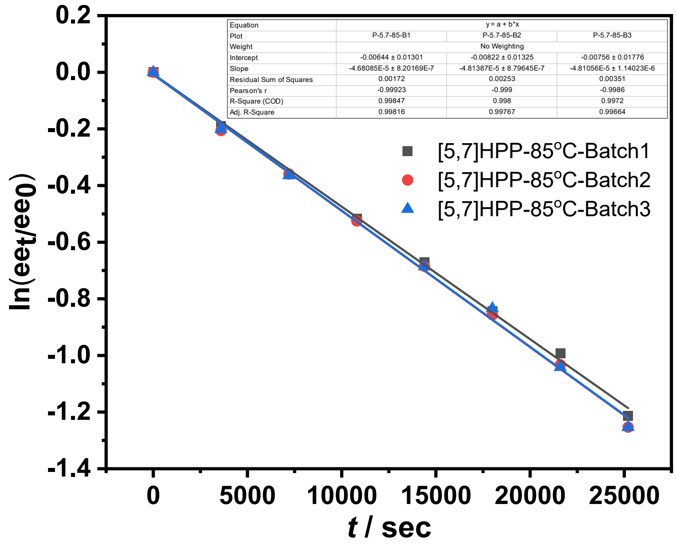

Figure S46. First order decay plots from three independent measurements for **[5,7]HPP** at 85°C.

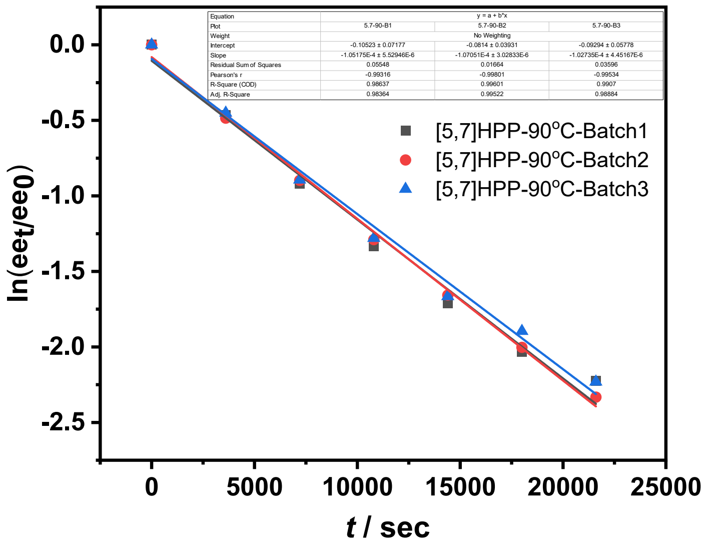

Figure S47. First order decay plots from three independent measurements for **[5,7]HPP** at 90°C.

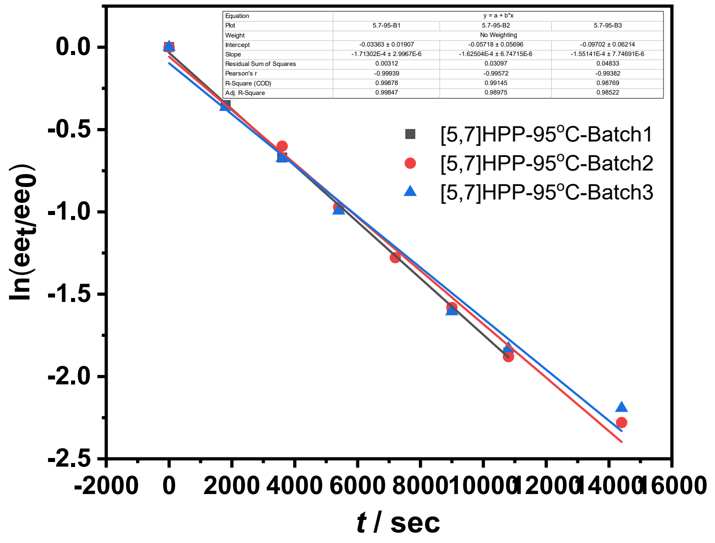

Figure S48. First order decay plots from three independent measurements for **[5,7]HPP** at 95ºC.

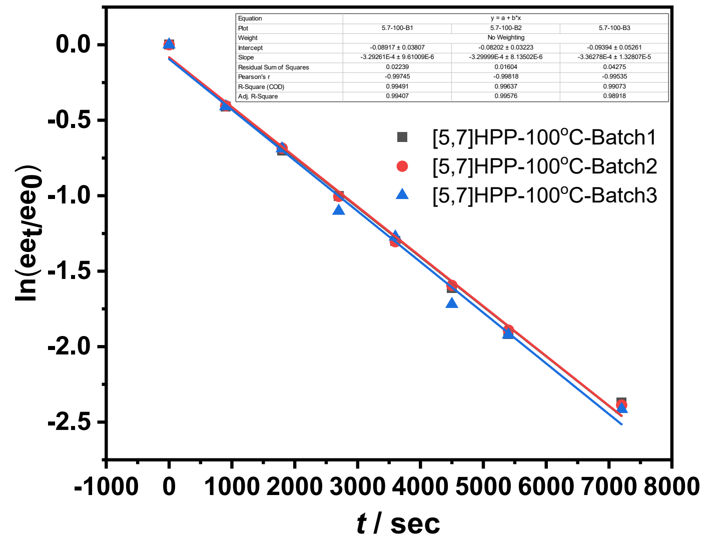

Figure S49. First order decay plots from three independent measurements for **[5,7]HPP** at 100ºC.

Table S13. Rate constants and Racemization kinetics data for **[5,5]HPP**.

	T (ºC)	T (kelvin)	k_{rac} (s^{-1})	k_{en} (s^{-1})	half-life (min)
Batch 1	50	323	6.26E-05	3.13E-05	369
Batch 2	50	323	6.89E-05	3.45E-05	335
Batch 1	55	328	1.31E-04	6.57E-05	175
Batch 2	55	328	1.34E-04	6.70E-05	172
Batch 3	55	328	1.28E-04	6.39E-05	180
Batch 1	60	333	2.27E-04	1.13E-04	102
Batch 2	60	333	2.33E-04	1.16E-04	99
Batch 3	60	333	2.27E-04	1.14E-04	101
Batch 1	65	338	4.79E-04	2.40E-04	48

Batch 2	65	338	4.35E-04	2.17E-04	53
Batch 3	65	338	4.37E-04	2.18E-04	52

Table S14. Rate constants and Racemization kinetics data for **[5,6]HPP**.

	T (ºC)	T (kelvin)	k_{rac} (s^{-1})	k_{en} (s^{-1})	half-life (min)
Batch 1	75	348	3.41E-5	1.70E-5	677
Batch 2	75	348	3.43E-5	1.71E-5	673
Batch 3	75	348	3.40E-5	1.70E-5	679
Batch 1	80	353	5.93E-5	2.96E-5	389
Batch 2	80	353	5.77E-5	2.88E-5	400
Batch 3	80	353	5.98E-5	2.99E-5	386
Batch 1	85	358	1.32E-4	6.59E-5	175
Batch 2	85	358	1.30E-4	6.52E-5	177
Batch 3	85	358	1.30E-4	6.50E-5	177
Batch 1	90	363	2.92E-4	1.46E-4	79
Batch 2	90	363	3.17E-4	1.58E-4	72
Batch 3	90	363	3.09E-4	1.54E-4	74

Table S15. Rate constants and Racemization kinetics data for **[5,7]HPP**.

	T (ºC)	T (kelvin)	k_{rac} (s^{-1})	k_{en} (s^{-1})	half-life (min)
Batch 1	85	358	4.68E-5	2.34E-5	493
Batch 2	85	358	4.81E-5	2.40E-5	479
Batch 3	85	358	4.81E-5	2.40E-5	480
Batch 1	90	363	1.05E-4	5.26E-5	219
Batch 2	90	363	1.07E-4	5.35E-5	215
Batch 3	90	363	1.03E-4	5.14E-5	224
Batch 1	95	368	1.71E-4	8.56E-5	134
Batch 2	95	368	1.62E-4	8.12E-5	142
Batch 3	95	368	1.55E-4	7.76E-5	148
Batch 1	100	373	3.29E-4	1.65E-4	70
Batch 2	100	373	3.30E-4	1.65E-4	70
Batch 3	100	373	3.36E-4	1.68E-4	68

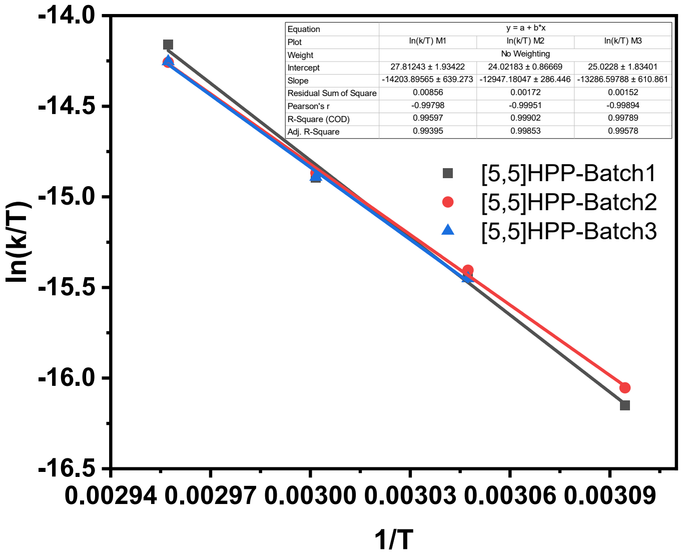

Figure S50. Eyring plot for [5,5]HPP.

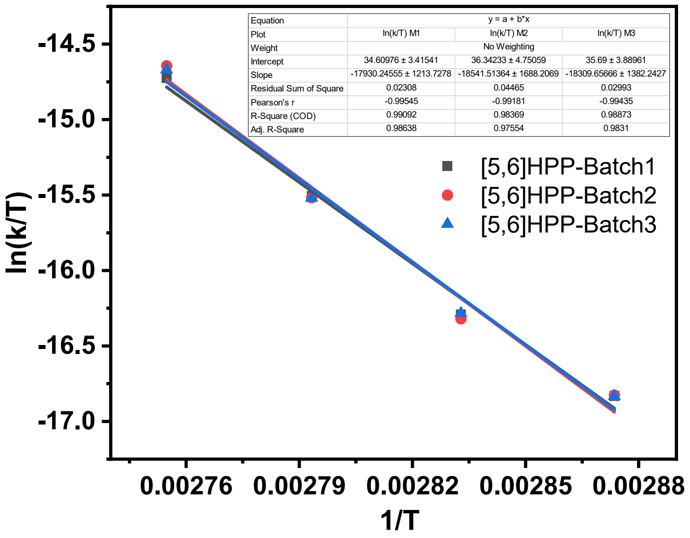

Figure S51. Eyring plot for [5,6]HPP.

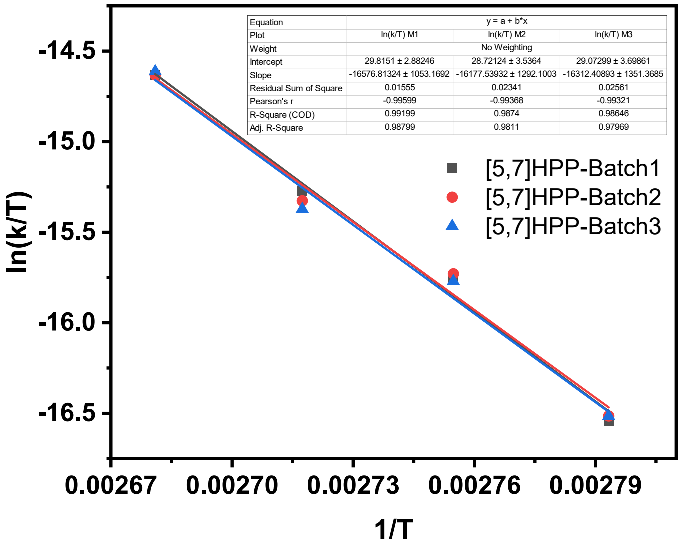

Figure S52. Eyring plot for [5,7]HPP.

Table S16: Thermodynamic data **[5,5]HPP** (enthalpy $\Delta H^{\ddagger}_{en}$ and entropy $\Delta S^{\ddagger}_{en}$).

	Slope	Intercept	$\Delta H^{\ddagger}$ (kcal/mol)	$\Delta S^{\ddagger}$ (cal/molK)	$\Delta H^{\ddagger}_{avg}$ (kcal/mol)	$\Delta S^{\ddagger}_{avg}$ (cal/molK)
Batch 1	-14203.90	27.81	28.2	8.0	26.8 ± 2.62	3.7 ± 7.92
Batch 2	-12947.18	24.02	25.7	0.5		
Batch 3	-13286.60	25.02	26.4	2.5		

Table S17: Activation barriers, $\Delta G^{\ddagger}_{en}$ for **[5,5]HPP**.

T (kelvin)	Batch 1	Batch 2	Batch 3	$\Delta G^{\ddagger}$ (kcal/mol)
323	25.6	25.6	25.6	25.6 ± 0.06
328	25.6	25.5	25.6	25.6 ± 0.03
333	25.5	25.5	25.6	25.5 ± 0.02
338	25.5	25.5	25.5	25.5 ± 0.06

Table S18: Thermodynamic data **[5,6]HPP** (enthalpy $\Delta H^{\ddagger}_{en}$ and entropy $\Delta S^{\ddagger}_{en}$).

	Slope	Intercept	$\Delta H^{\ddagger}$ (kcal/mol)	$\Delta S^{\ddagger}$ (cal/molK)	$\Delta H^{\ddagger}_{avg}$ (kcal/mol)	$\Delta S^{\ddagger}_{avg}$ (cal/molK)
Batch 1	-17930.24	34.61	35.6	21.6	36.3 ± 1.24	23.4 ± 3.48
Batch 2	-18541.51	36.34	36.8	25.0		
Batch 3	-18309.65	35.70	36.4	23.7		

Table S19: Activation barriers, $\Delta G^{\ddagger}_{en}$ for **[5,6]HPP**.

T (kelvin)	Batch 1	Batch 2	Batch 3	$\Delta G^{\ddagger}$ (kcal/mol)
348.15	28.1	28.1	28.1	28.1 ± 0.03
353.15	28.0	28.0	28.0	28.0 ± 0.01
358.15	27.9	27.9	27.9	27.9 ± 0.0
363.15	27.8	27.8	27.7	27.8 ± 0.02

Table S20: Thermodynamic data **[5,7]HPP** (enthalpy $\Delta H^{\ddagger}_{en}$ and entropy $\Delta S^{\ddagger}_{en}$).

	Slope	Intercept	ΔH‡ (kcal/mol)	ΔS‡ (cal/molK)	ΔH‡ (kcal/mol)	ΔS‡ (cal/molK)
Batch 1	-16576.81	29.81	32.9	12.0	32.5 ± 0.82	10.8 ± 2.25
Batch 2	-16177.54	28.72	32.1	9.9		
Batch 3	-16312.40	29.07	32.4	10.6		

Table S21: Activation barriers, $\Delta G^{\ddagger}_{en}$ for **[5,7]HPP**.

T (kelvin)	Batch 1	Batch 2	Batch 3	ΔG‡ (kcal/mol)
358	28.6	28.6	28.6	28.6 ± 0.02
363	28.6	28.6	28.6	28.6 ± 0.01
368	28.5	28.5	28.5	28.5 ± 0.02
373	28.5	28.5	28.5	28.5 ± 0.02

Optical and Chiroptical Properties

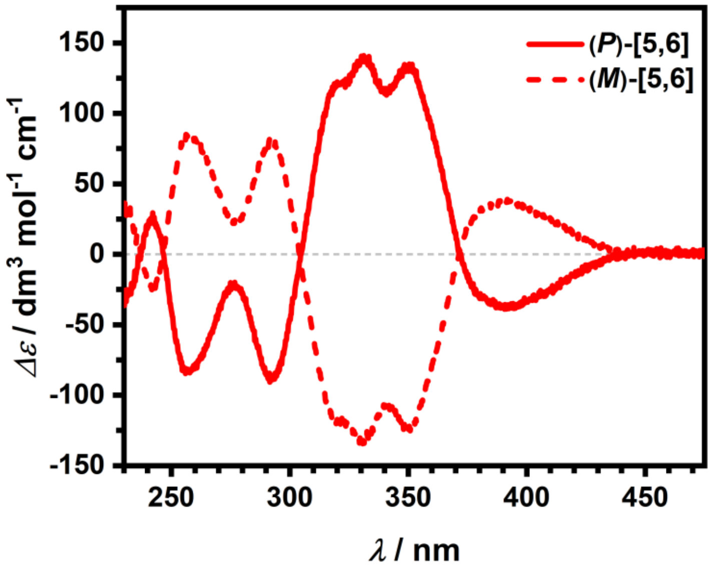

Figure S53. Circular dichroism spectra of **[5,6]HPP**.

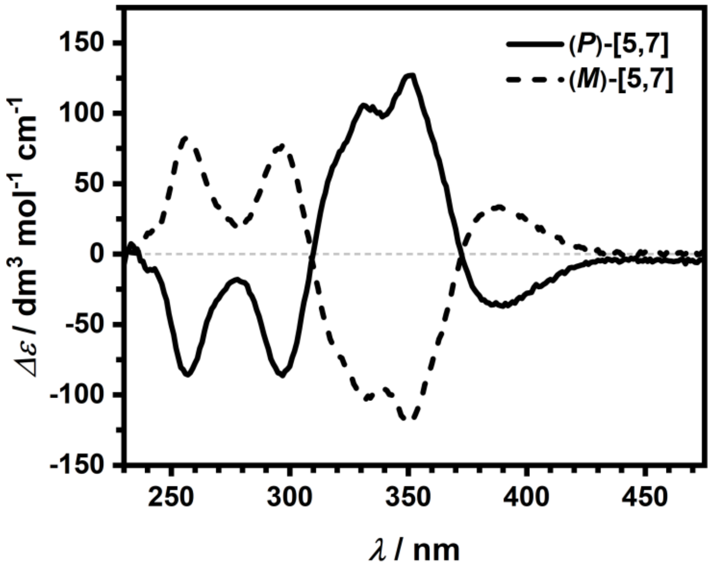

Figure S54. Circular dichroism spectra of **[5,7]HPP**.

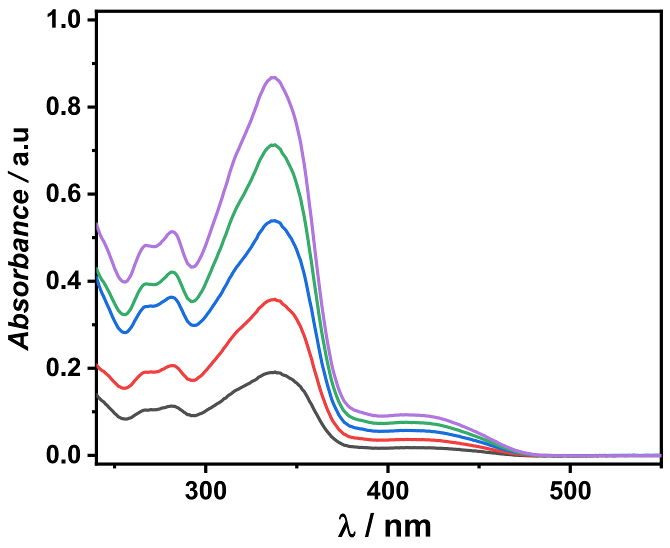

Figure S55. Absorption dilution series of **[5,5]HPP**.

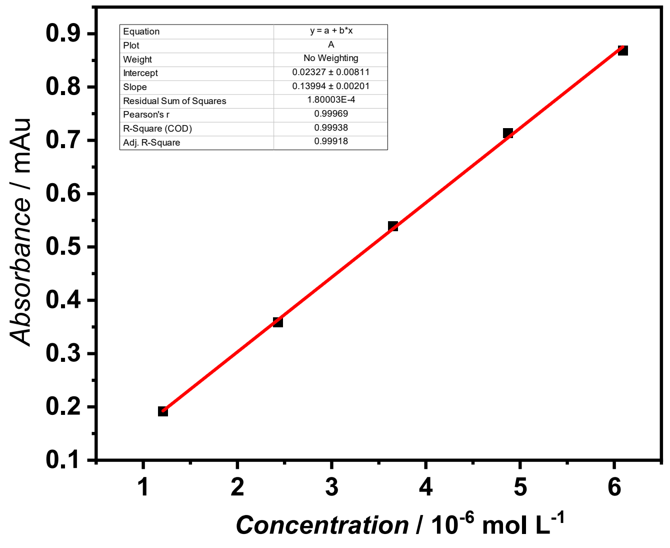

Figure S56. Graph of absorbance (λ = 338 nm) as a function of concentration for **[5,5]HPP**.

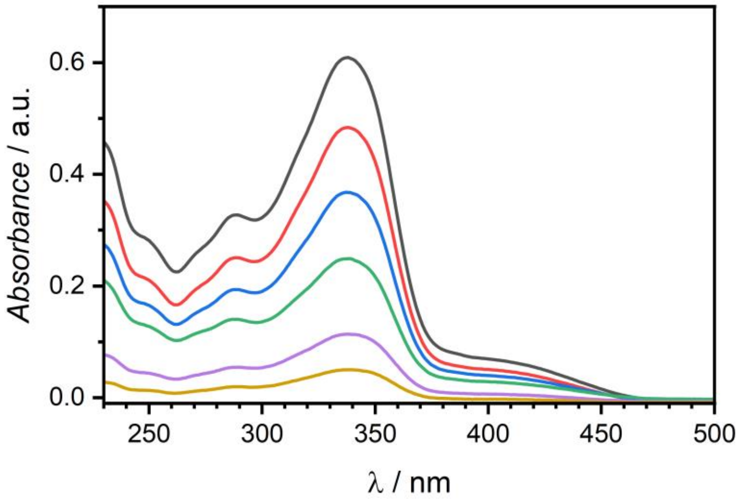

Figure S57. Absorption dilution series of **[5,6]HPP**.

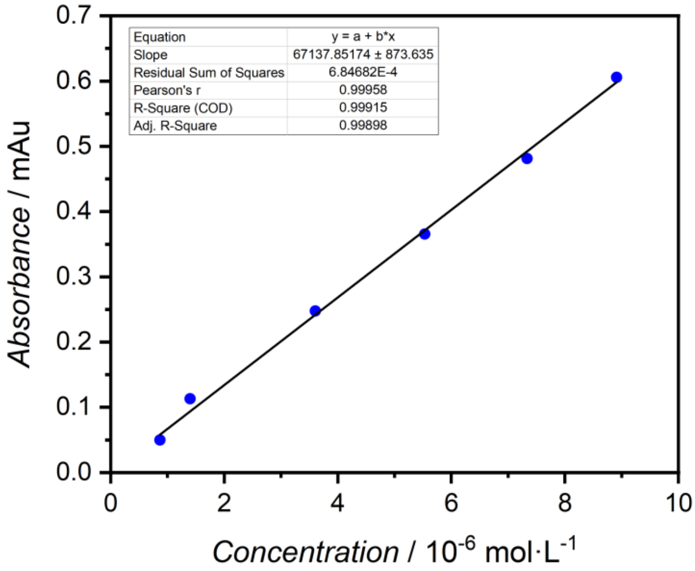

Figure S58. Graph of absorbance (λ = 340 nm) as a function of concentration for **[5,6]HPP**.

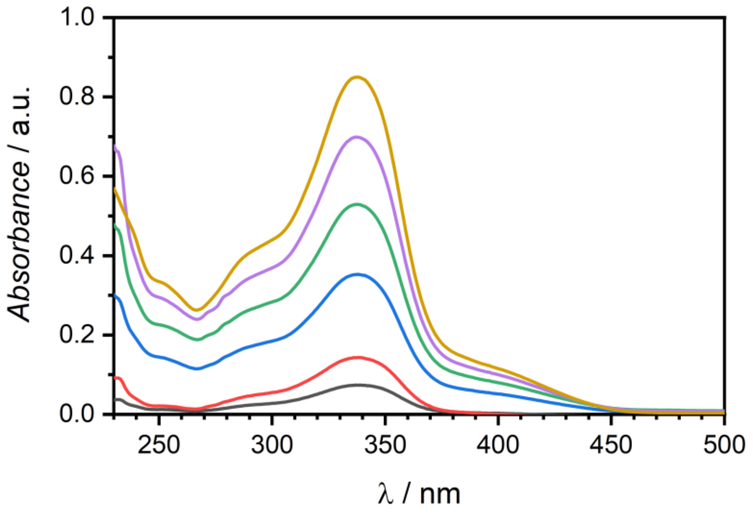

Figure S59. Absorption dilution series of **[5,7]HPP**.

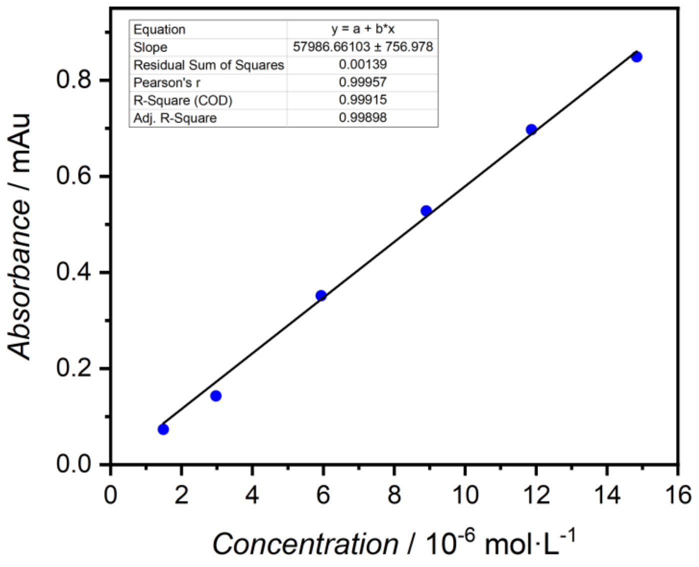

Figure S60. Graph of absorbance (λ = 340 nm) as a function of concentration for **[5,7]HPP**.

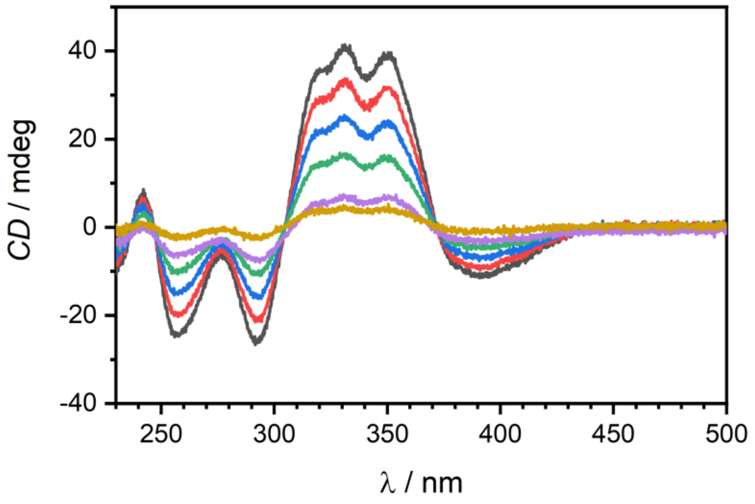

Figure S61. Circular dichroism dilution series of **(*P*)-[5,6]HPP**.

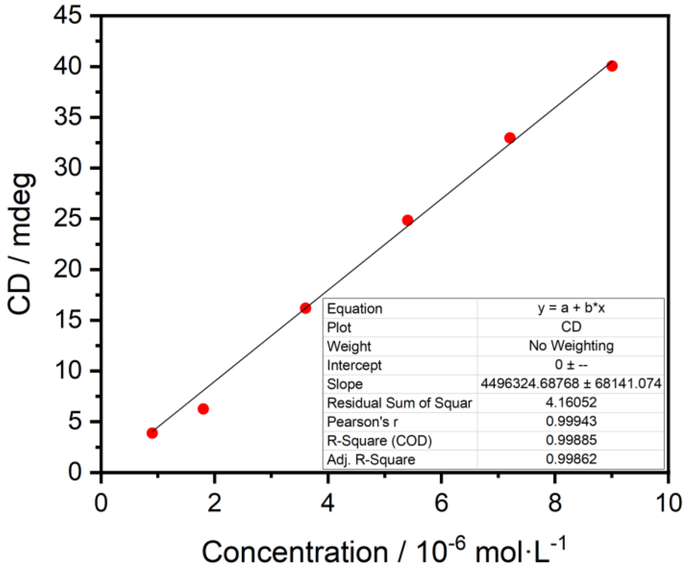

Figure S62. Graph of circular dichroism (λ = 330 nm) as a function of concentration for **(*P*)-[5,6]HPP**.

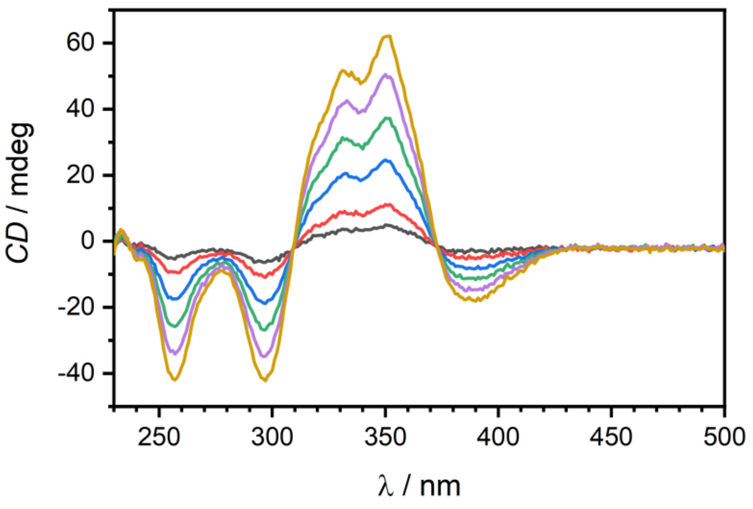

Figure S63. Circular dichroism dilution series of **(*P*)-[5,7]HPP**.

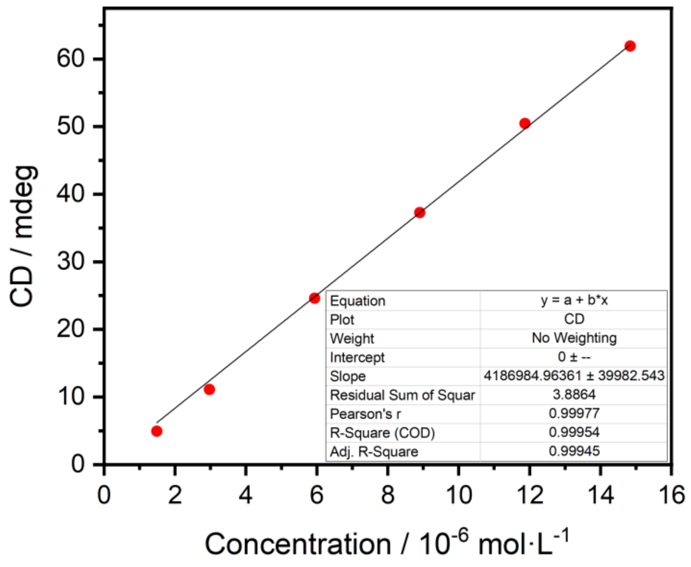

Figure S64. Graph of circular dichroism (λ = 350 nm) as a function of concentration for **(*P*)-[5,7]HPP**.

DFT Calculations

All calculations were performed with Gaussian 09[2] (release D.01) or Gaussian 16[3] (release C.02) software. Geometries of molecules were optimized at D3-B3LYP/6-31g(d) level of theory. The character of stationary points on potential energy surface was determined by subsequent frequency calculations. For transition states, IRC calculations were performed, accompanied by the full geometry optimization at the end of the IRC path, to find the conformers that are connected via the optimized transition states. The default UltraFine integration grid was used in all calculations.

A conformational search for **[5,6]HPP** and **[5,7]HPP** was performed using Avogadro software[4] at MMFF94 level of theory. A set of low energy conformers was identified and then optimized using D3-B3LYP functional with 6-31g(d) basis set. We found 7 and 11 unique conformers for **[5,6]HPP** and **[5,7]HPP**, respectively. Only two distinct conformers could be found for **[5,5]HPP**. The energies of these conformers were then obtained by single point calculations with cc-pVTZ basis set. The lowest energy conformers were then taken as the "ground state" from which the enantiomerization process takes place. The geometries of these three conformers are given in Figure S65.

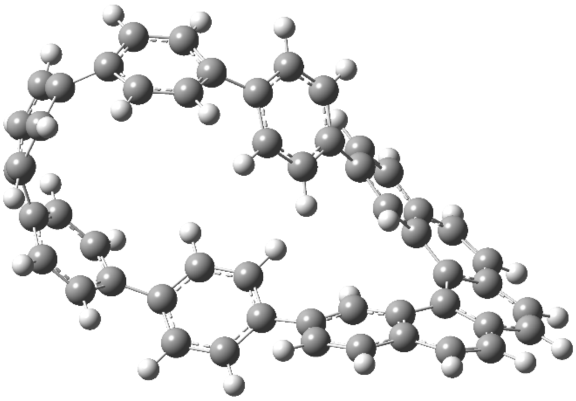

[5,5]HPP

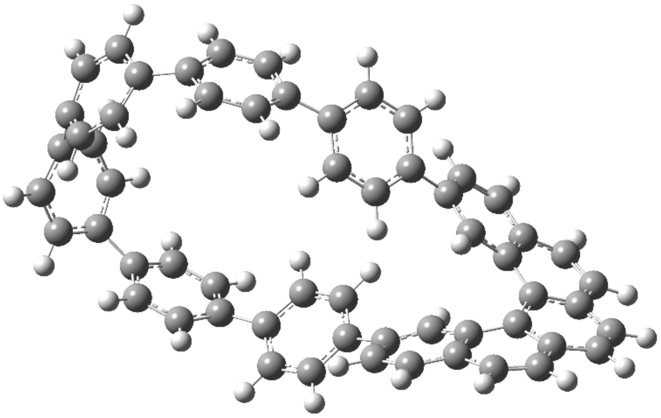

[5,6]HPP

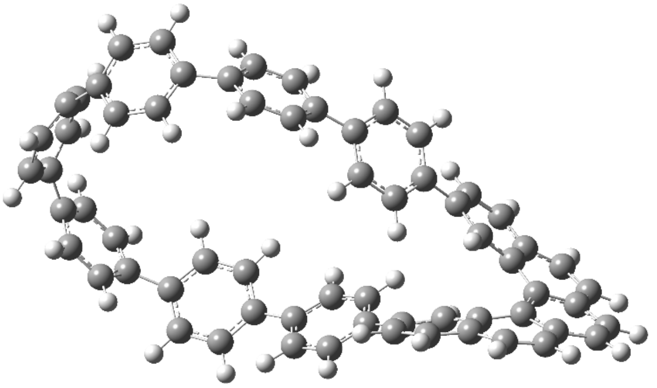

[5,7]HPP

Figure S65. The lowest energy conformation identified for [5,*m*]HPPs (m = 5−7) at the B3LYP-D3/cc-pVTZ level of theory on B3LYP-D3/6-31G(d) geometries.

Strain Energy and Activation Enthalpy Calculations

Strain energies were estimated via homodesmotic reactions (Scheme S1) on the lowest energy conformers of [5,*m*]HPPs (*m* = 5−7). Single point energies calculated on D3-B3LYP/cc-pVTZ level of theory were corrected by unscaled zero-point vibrational energy (ZPVE) correction from D3-B3LYP/6-31g(d). We also used StrainViz software developed by Jasti group[5] to visualize local strain of [5,*m*]HPPs (*m* = 6,7) and estimate total strain energy, which was nearly identical to the strain energy obtained via homodesmotic reactions. In addition, we calculated the strain energies of the [5]helicene fragments in the structure of the [5,*m*]HPPs (*m* = 5−7) lowest energy minima ("ground state") and in the corresponding transition states of enantiomerization. The fragments were obtained by trimming all parts of the molecule except the [5]helicene unit to which two hydrogen atoms were attached at 2,13-positions to fill the valency. Subsequently, all coordinates were frozen except of the two new hydrogen atoms to preserve the geometries of the fragment that it had in the corresponding structure. After the full optimization of the six Cartesian coordinates of the two hydrogen atoms on the B3LYP-D3/6-31G(d) level of theory, the strain energy was then calculated as the energy difference of the fragment with respect to the fully relaxed [5]helicene energy minimum or the C_s transition state of its enantiomerization. The used energies were obtained by single point energy calculations at the B3LYP-D3/cc-pVTZ level of theory.

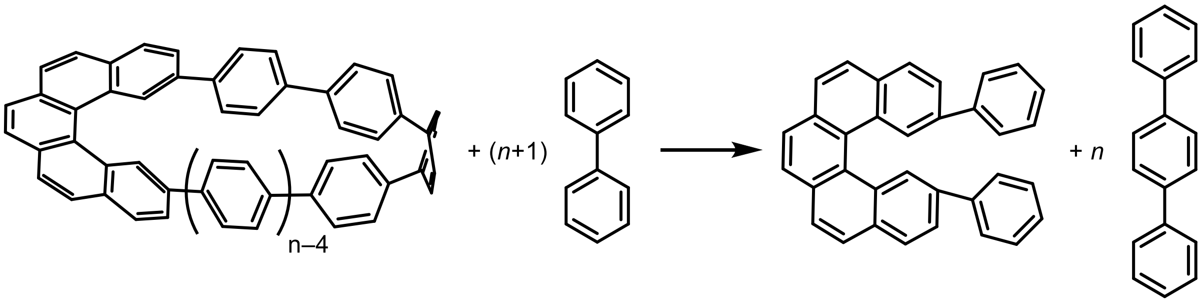

Scheme S1.

Table S12. Thermochemistry for the homodesmotic reactions shown in Scheme S1. The B3LYP-D3/cc-pVTZ electronic energies and their corrections by adding the zero-point vibrational energies (ZPVEs) are listed.

Molecule	ZPVE / Hartree	E_{el} / Hartree	E_{corr} / Hartree
9,12-diphenyl[5]helicene	0.451325	–1309.411202500	–1308.959877500
biphenyl	0.182137	–463.481180258	–463.299043258
terphenyl	0.263352	–694.624576988	–694.361224988
**[5,5]HPP**	0.672603	–2001.54576908	–2000.87316608
**[5,6]HPP**	0.754060	–2232.69951195	–2231.94546295
**[5,7]HPP**	0.835454	–2463.851801940	–2463.016347940

Table S2. Calculated strain energies of [5,*m*]HPPs ($m = 5-7$).

Molecule	Strain Energy[a] / kcal mol^{-1}	Strain Energy[b] / kcal mol^{-1}
**[5,5]HPP**	61.9	n.a.[c]
**[5,6]HPP**	55.5	55.1
**[5,7]HPP**	50.0	50.4

[a]Strain energy obtained via homodesmotic reaction (Scheme S1) and B3LYP method (see Table S22). [b]Strain energy obtained via StrainViz. [c]Not available.

Table S3. Calculated strain energies of [5]helicene fragments in the energy minima and transition states of 2,13-diphenyl-[5]helicene and [5,*m*]HPPs ($m = 5-7$).

Molecule	Geometry	Strain Energy[a] / kcal mol^{-1}
2,13-diphenyl-[5]helicene	energy minimum	0.9
	transition state	0.6
**[5,5]HPP**	energy minimum	1.1
	transition state	3.5
**[5,6]HPP**	energy minimum	0.9
	transition state	2.6
**[5,7]HPP**	energy minimum	0.9
	transition state	2.2

[a]Strain energy obtained as the energy difference of the fully relaxed [5]helicene in its lowest energy minimum or the enantiomerization transition state and the energy of the [5]helicene fragment from the corresponding energy minima or transition states of [5,*m*]HPPs ($m = 5-7$). Using B3LYP method (see Table S22)

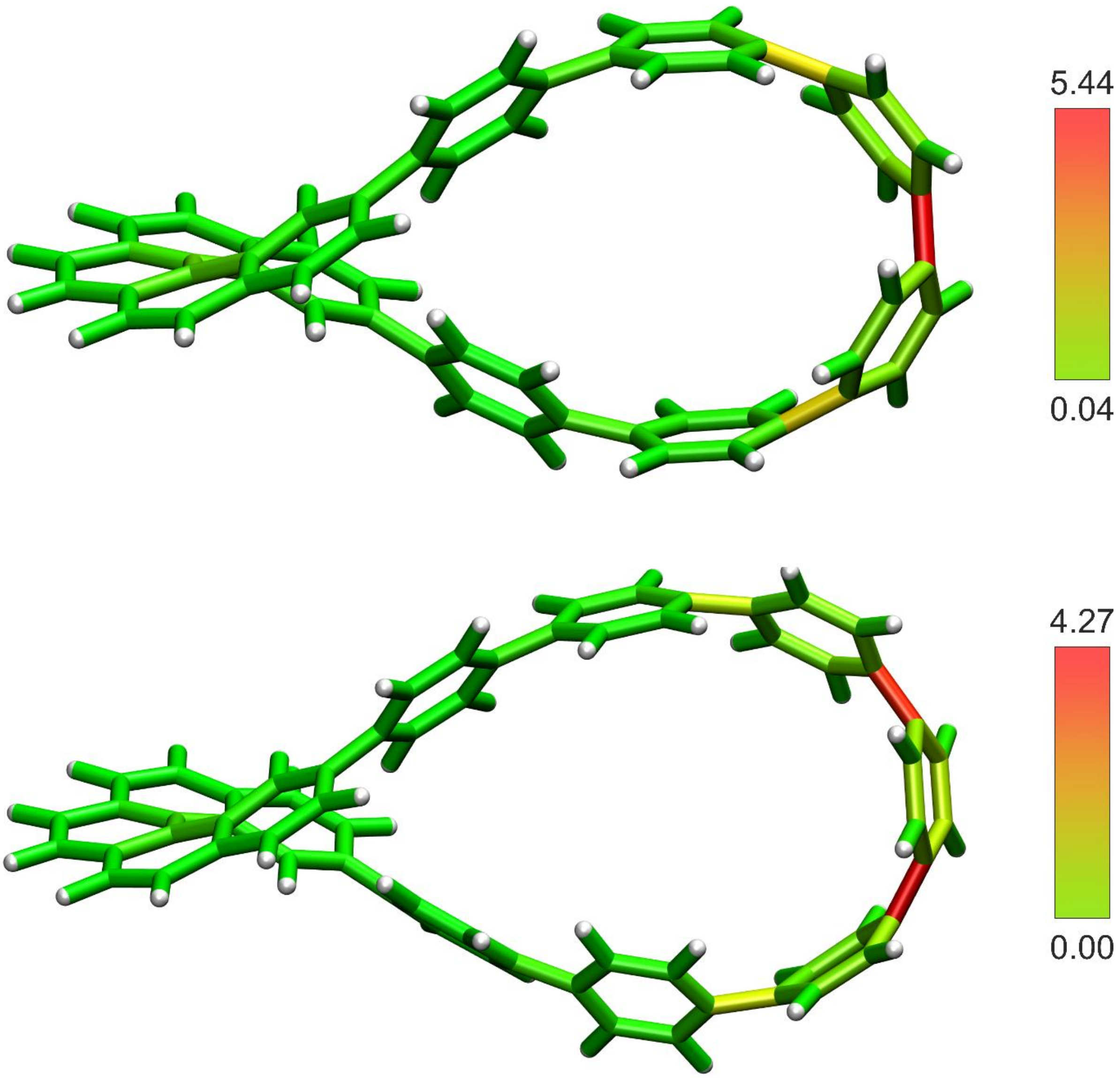

Figure S66. Total strain visualization of [5,*m*]HPPs (m = 6,7) by StrainViz with energies in kcal mol^{-1}.

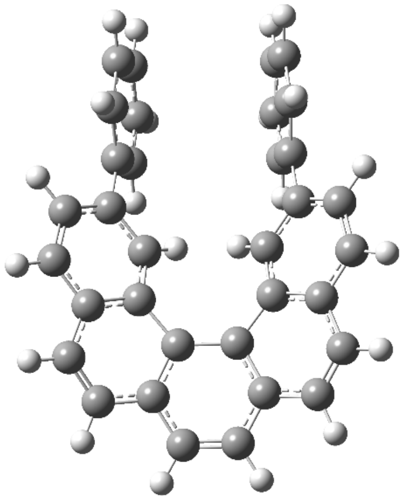

2,13-diphenyl-[5]helicene

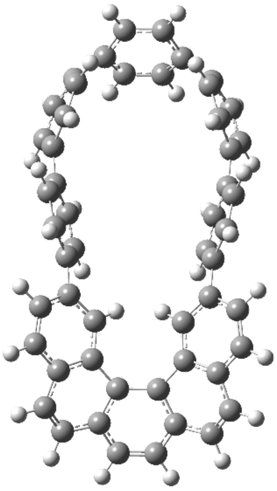

[5,5]HPP

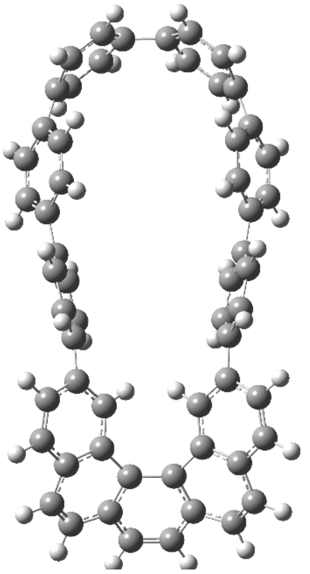

[5,6]HPP

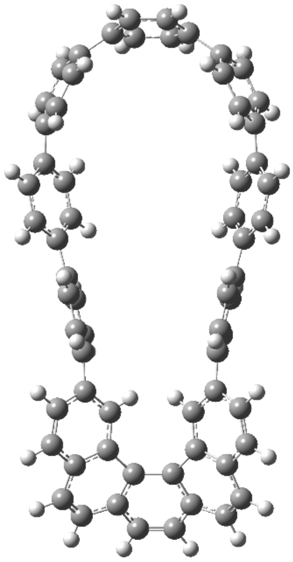

[5,7]HPP

Figure S67. Transition state geometries of [5,*m*]HPPs (*m* = 5-7) and 2,13-diphenyl-[5]helicene.

Table S4. Calculated energies of activation[a,b] (at 0K) of enantiomerization of [5]helicene, 2,13-diphenyl-[5]helicene, and [5,*m*]HPPs (m = 5−7).

Molecule	$\Delta H^{\ddagger}$ / kcal mol^{-1}				
	B3LYP-D3	wB97XD	M06-2X-D3	MN15	BMK-D3
[5]helicene	23.8	24.6	25.3	24.7	25.1
2,13-diphenyl-[5]helicene	24.9	25.5	26.8	26.2	27.3
**[5,5]HPP**	25.6	25.9	26.5	26.2	27.2
**[5,6]HPP**	28.0	29.3	29.7	29.4	30.1
**[5,7]HPP**	29.2	29.9	30.6	30.6	31.3
**[5,8]HPP**	30.1	31.1	31.8	31.6	33.0

[a]The enthalpy correction to 298 K decreases the barriers by 0.3−0.4 kcal mol^{-1} for all transition states. [b]With cc-pVTZ basis set on B3LYP-D3/6-31G(d) geometries; energies involve unscaled ZPVE correction.

Table S5. Calculated geometry parameters[a] d_{1-14} and θ in the energy minima of [5]helicene, 2,13-diphenyl-[5]helicene, and [5,*m*]HPPs (m = 5−7).

Molecule	d_{1-14} / Å	θ / °
[5]helicene	2.949	22.06
2,13-diphenyl-[5]helicene	2.914	22.24
**[5,5]HPP**	3.035	22.76
**[5,6]HPP**	2.999	22.9
**[5,7]HPP**	2.946	22.58

[a]Parameters are defined according to ref[6]. Briefly, d_{1-14} is the C1–C14 distance in Å and the twist angle θ is calculated as an average value of torsional angles of all phenanthrene subunits of [5]helicene fragments in individual compounds.

Table S6. Calculated entropies (in cal mol^{-1} K^{-1}) of [5]helicene, 2,13-diphenyl-[5]helicene, [5,*m*]HPPs (m = 5−7), and their transition states of enantiomerization.

Molecule	Energy minimum	Transition state	Enantiomerization	T$\Delta S^{\ddagger}$ (kcal mol^{-1}), 298.15 K
[5]helicene	117.8	116.7	–1.1	–0.3
2,13-diphenyl-[5]helicene	164.5	162.4	–2.1	–0.6
**[5,5]HPP**	219.1	212.7	–6.4	–1.9
**[5,6]HPP**	237.1	236.4	–0.7	–0.2
**[5,7]HPP**	260.8	258.8	–2.0	–0.6

[a]Parameters are defined according to ref[6]. Briefly, d_{1-14} is the C1–C14 distance in Å and the twist angle θ is calculated as an average value of torsional angles of all phenanthrene subunits of [5]helicene fragments in individual compounds.

TD-DFT

CD Spectra

The absolute configurations of respective enantiomers of [5,*m*]HPPs (*m* = 5−7) were assigned by comparison of the calculated and measured CD spectra. The CD spectra were obtained calculating first 50 singlet excitations on ground state geometries at CAM-B3LYP/6-31g(d) level of theory. Solvent effects were simulated using PCM model. The ECD spectra were compared using SpecDis (release 1.71).[7] Empirical vibrational broadening of 0.16 eV was used and the calculated spectra were shifted by –0.20 eV for **[5,6]HPP** and by –0.3 eV for **[5,7]HPP**. The velocity form of the rotatory strength was used. A good agreement of the experimental and the calculated data was observed.

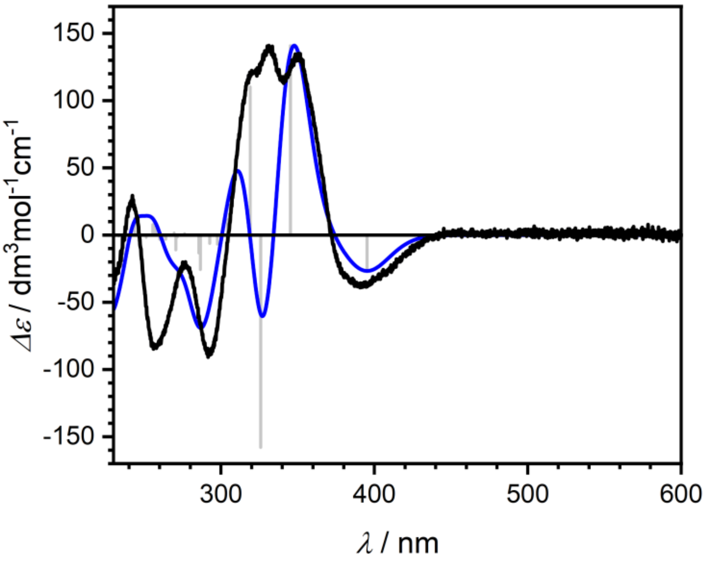

Figure S68. Experimental (black) and calculated (blue) CD spectra of ***(P)*-[5,6]HPP** with excitation transitions (grey lines) in DCM.

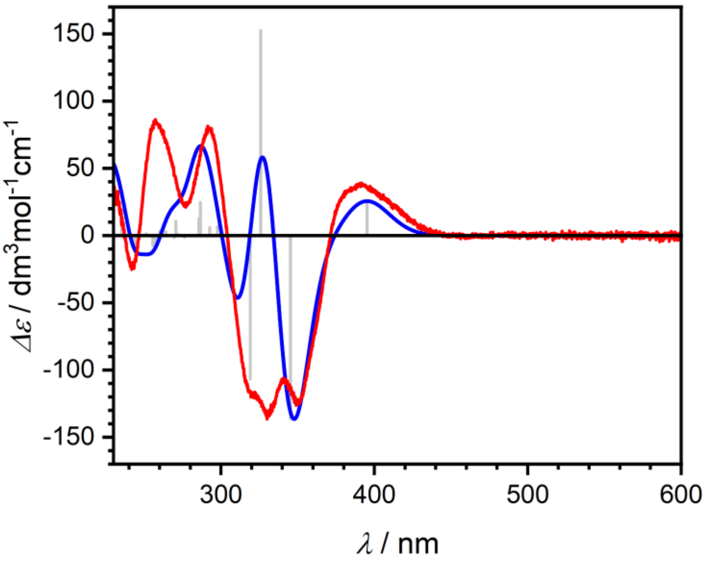

Figure S69. Experimental (red) and calculated (blue) CD spectra of ***(M)*-[5,6]HPP** with excitation transitions (grey lines) in DCM.

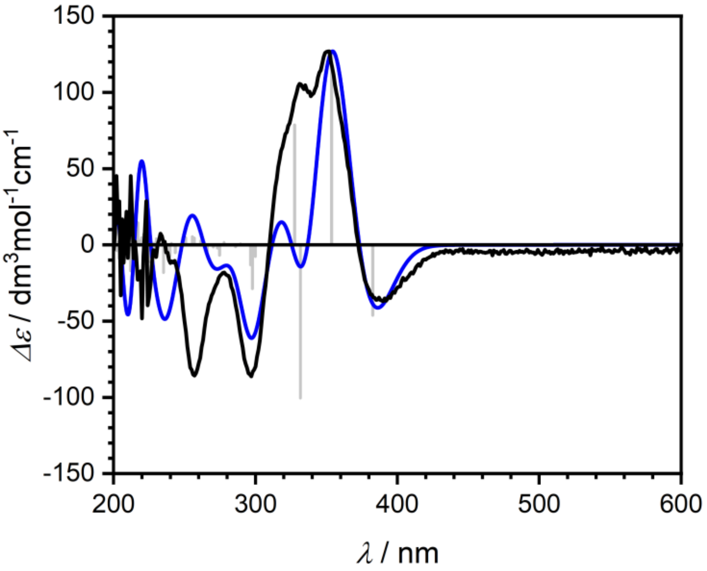

Figure S70. Experimental (black) and calculated (blue) CD spectra of ***(P)*-[5,7]HPP** with excitation transitions (grey lines) in DCM.

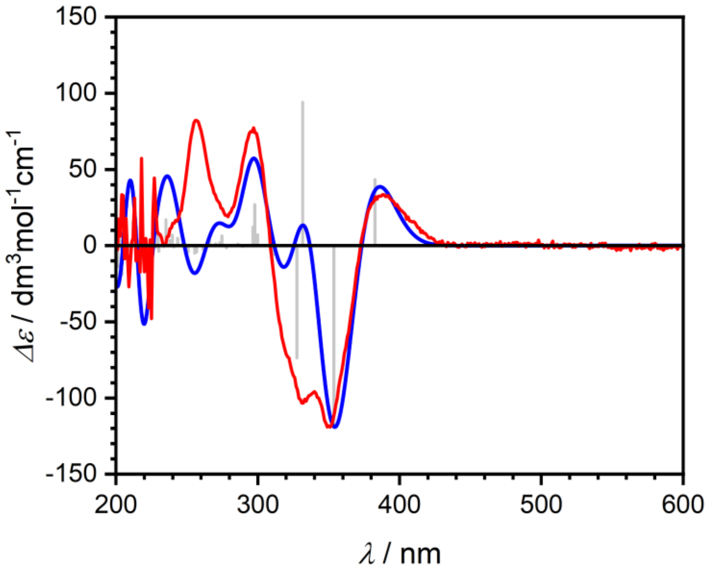

Figure S71. Experimental (red) and calculated (blue) CD spectra of ***(M)*-[5,7]HPP** with excitation transitions (grey lines) in DCM.

Excited-state Lifetime Measurements

The excited state lifetimes of the compounds were determined from luminescence intensity decay curves using time-correlated single photon counting (TCSPC) method performed on a LifeSpec II spectrometer (Edinburgh Instruments) employing a picosecond pulsed diode laser (ca. 60 ps pulse width). The excitation wavelength used to excite the degassed CH_2Cl_2 samples (c ~ 10^{-6} M) was 405 nm. The decay curves were fitted with a single exponential convoluted with the instrument response.

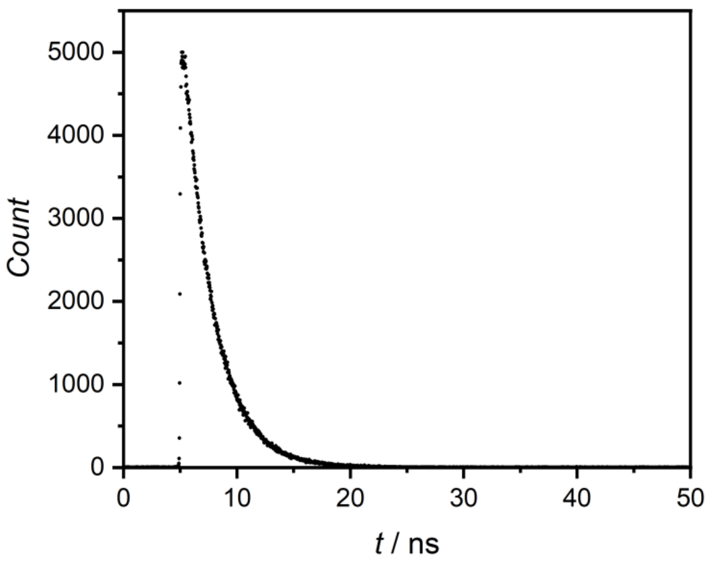

Figure S72. Lifetime measurement of **[5,6]HPP**, excitation at 405 nm and detection at 510 nm in CH_2Cl_2.

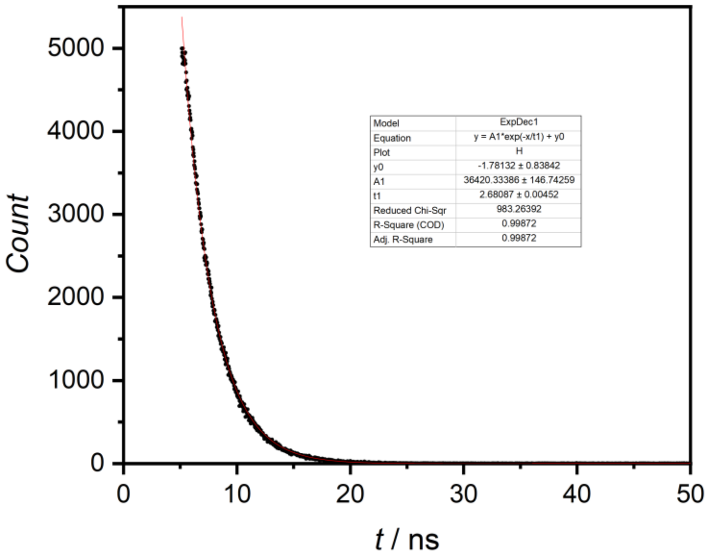

Figure S73. Lifetime measurement of **[5,6]HPP**, excitation at 405 nm and detection at 510 nm with a fitted curve.

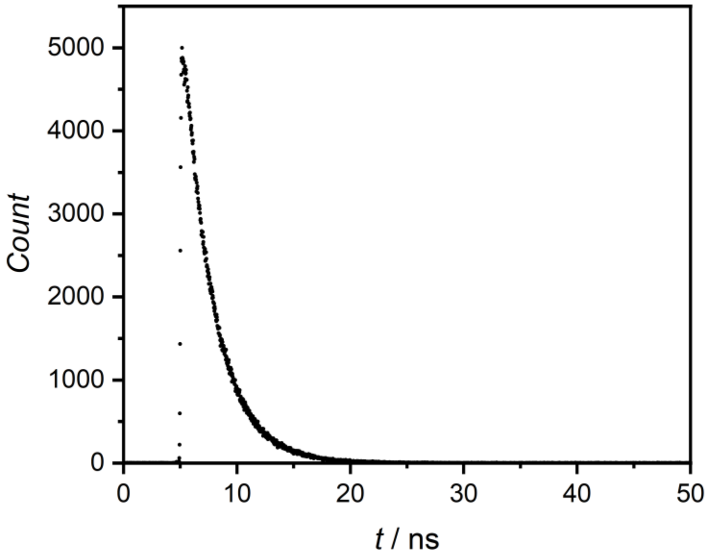

Figure S74. Lifetime measurement of **[5,7]HPP**, excitation at 405 nm and detection at 510 nm in CH_2Cl_2.

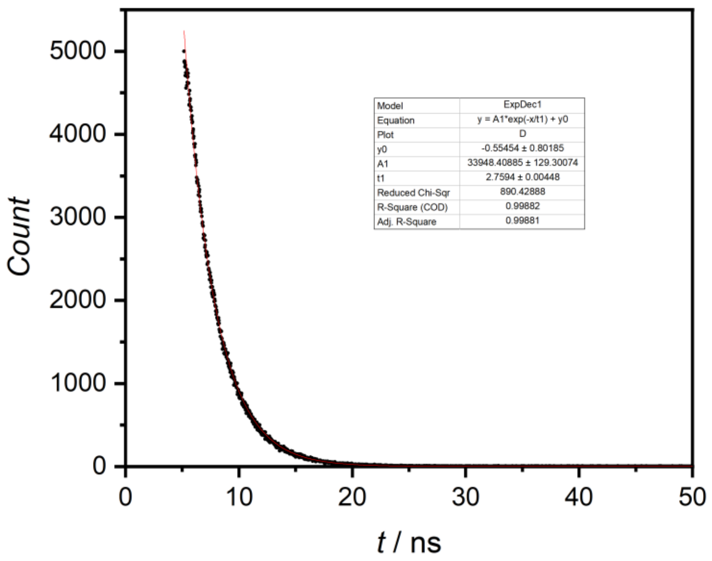

Figure S75. Lifetime measurement of **[5,7]HPP**, excitation at 405 nm and detection at 510 nm with a fitted curve.

Cartesian coordinates

[5]helicene, E_{el} (B3LYP-D3/6-31G(d)) = −846.841951461 Hartree, Imaginary Frequencies = 0

6	0.164049	0.662811	3.249319
6	-0.164049	-0.662811	3.249319
6	0.236581	1.387249	2.025660
6	-0.236581	-1.387249	2.025660
6	0.005188	0.724775	0.788022
6	-0.005188	-0.724775	0.788022
6	0.506844	2.792724	2.050963
6	0.458772	3.542924	0.915941
6	-0.005188	2.966194	-0.307278
6	-0.295061	1.566497	-0.365162
1	0.312771	1.199655	4.182882
1	-0.312771	-1.199655	4.182882
1	0.758180	3.248774	3.005507
1	0.691594	4.604642	0.939540
6	-0.266626	3.786257	-1.432725
6	-0.987194	1.095247	-1.511055
6	-1.267471	1.922511	-2.580325
6	-0.874323	3.276142	-2.560292
1	-0.009267	4.841401	-1.375353
1	-1.318913	0.065365	-1.542057
1	-1.805544	1.526349	-3.437179
1	-1.081565	3.918528	-3.411753
6	-0.458772	-3.542924	0.915941
6	-0.506844	-2.792724	2.050963
6	0.005188	-2.966194	-0.307278
6	0.295061	-1.566497	-0.365162
6	0.266626	-3.786257	-1.432725
6	0.874323	-3.276142	-2.560292
6	1.267471	-1.922511	-2.580325
6	0.987194	-1.095247	-1.511055
1	-0.691594	-4.604642	0.939540
1	-0.758180	-3.248774	3.005507
1	0.009267	-4.841401	-1.375353
1	1.081565	-3.918528	-3.411753
1	1.318913	-0.065365	-1.542057
1	1.805544	-1.526349	-3.437179

Enantiomerization transition state (TS) of [5]helicene, E_{el} (B3LYP-D3/6-31G(d)) = −846.801512177 Hartree, Imaginary Frequencies = 1 (116.9 cm^{-1})

6	-3.220220	0.501542	-0.679759
6	-3.220220	0.501542	0.679759
6	-2.012695	0.265411	-1.383787
6	-2.012695	0.265411	1.383787
6	-0.734157	0.342249	-0.737545
6	-0.734157	0.342249	0.737545
6	-2.162941	-0.207939	-2.728827
6	-1.105684	-0.697609	-3.419919
6	0.213619	-0.477032	-2.920567
6	0.409139	0.206074	-1.669023
1	-4.149823	0.564859	-1.239332
1	-4.149823	0.564859	1.239332
1	-3.169423	-0.268247	-3.134615
1	-1.231899	-1.171968	-4.389624
6	1.314590	-0.799409	-3.747928
6	1.694473	0.774524	-1.515114
6	2.756221	0.501379	-2.362016
6	2.588563	-0.355198	-3.459151
1	1.122106	-1.364378	-4.657017
1	1.844593	1.535348	-0.775853
1	3.714613	0.980633	-2.181629
1	3.424377	-0.600203	-4.108385
6	-1.105684	-0.697609	3.419919
6	-2.162941	-0.207939	2.728827
6	0.213619	-0.477032	2.920567
6	0.409139	0.206074	1.669023
6	1.314590	-0.799409	3.747928
6	2.588563	-0.355198	3.459151
6	2.756221	0.501379	2.362016
6	1.694473	0.774524	1.515114
1	-1.231899	-1.171968	4.389624
1	-3.169423	-0.268247	3.134615
1	1.122106	-1.364378	4.657017
1	3.424377	-0.600203	4.108385
1	1.844593	1.535348	0.775853
1	3.714613	0.980633	2.181629

2,13-diphenyl-[5]helicene, E_{el} (B3LYP-D3/6-31G(d)) = −1308.98550355 Hartree, Imaginary Frequencies = 0

6	-2.012160	2.024270	1.199930
6	-2.402220	0.736590	1.609770
6	-2.770160	3.120630	1.646030
6	-3.511220	0.554370	2.433540
1	-1.850400	-0.130210	1.260300
6	-3.879640	2.937530	2.470000
1	-2.471480	4.127210	1.366590
6	-4.256490	1.652850	2.866680
1	-3.799320	-0.451570	2.727130
1	-4.445840	3.801190	2.809080
6	-2.012160	-2.024270	-1.199930
6	-2.770160	-3.120630	-1.646030
6	-2.402220	-0.736590	-1.609770
6	-3.879640	-2.937530	-2.470000
1	-2.471480	-4.127210	-1.366580
6	-3.511220	-0.554370	-2.433540
1	-1.850400	0.130210	-1.260310
6	-4.256490	-1.652850	-2.866680
1	-4.445840	-3.801190	-2.809080
1	-3.799310	0.451570	-2.727130
1	-1.697470	3.873340	-0.797920
6	-0.819170	3.249480	-0.661200
6	-0.836690	2.218340	0.315000
6	0.307630	3.471530	-1.419860
1	0.335050	4.297430	-2.126880
6	0.276620	1.394460	0.422290
1	0.286570	0.637910	1.195630
6	1.458720	2.656300	-1.286590
6	1.413540	1.530190	-0.409600
6	2.681140	2.990690	-1.946190
1	2.697510	3.844190	-2.619410
6	3.824750	2.308660	-1.655390
6	2.573220	0.651600	-0.314120
6	3.808200	1.156130	-0.806080
1	4.777410	2.622560	-2.075020
6	2.573220	-0.651600	0.314120
6	1.413540	-1.530190	0.409600
6	-0.819170	-3.249480	0.661200
6	0.307630	-3.471530	1.419860
6	-0.836690	-2.218340	-0.315000
6	1.458720	-2.656300	1.286590
6	5.031450	0.531240	-0.429670
6	0.276620	-1.394460	-0.422290
6	3.808200	-1.156130	0.806080
1	-1.697470	-3.873340	0.797920
6	2.681140	-2.990690	1.946190
6	5.031450	-0.531240	0.429670
1	0.335050	-4.297420	2.126880
6	3.824750	-2.308660	1.655390
1	5.965720	0.953230	-0.791530
1	2.697510	-3.844190	2.619410
1	4.777410	-2.622560	2.075020
1	5.965720	-0.953230	0.791520
1	0.286570	-0.637910	-1.195630
1	-5.122840	1.509570	3.506860
1	-5.122830	-1.509570	-3.506860

Enantiomerization TS of 2,13-diphenyl-[5]helicene, E_{el} (B3LYP-D3/6-31G(d)) = −1308.94227643 Hartree, Imaginary Frequencies = 1 (58.3 cm^{-1})

6	1.150513	-5.011688	0.680216
6	1.150513	-5.011688	-0.680216
6	0.613899	-3.904155	1.384125
6	0.613899	-3.904155	-1.384125
6	0.371248	-2.647936	0.735817
6	0.371248	-2.647936	-0.735817
6	0.175581	-4.168198	2.723800
6	-0.585007	-3.271025	3.398308
6	-0.702333	-1.941329	2.893623
6	-0.057395	-1.575131	1.661721
1	1.444900	-5.896095	1.239276
1	1.444900	-5.896095	-1.239276
1	0.368656	-5.155568	3.135010
1	-1.031006	-3.513444	4.359366
6	-1.319822	-0.952279	3.692389
6	0.185695	-0.193637	1.515062
6	-0.359773	0.786942	2.341146
6	-1.198046	0.389084	3.403202
1	-1.845512	-1.271303	4.589295
1	0.889749	0.137313	0.778134
1	-1.650025	1.137072	4.047931
6	-0.585007	-3.271025	-3.398308
6	0.175581	-4.168198	-2.723800
6	-0.702333	-1.941329	-2.893623
6	-0.057395	-1.575131	-1.661721
6	-1.319822	-0.952279	-3.692389
6	-1.198046	0.389084	-3.403202
6	-0.359773	0.786942	-2.341146
6	0.185695	-0.193637	-1.515062
1	-1.031006	-3.513444	-4.359366
1	0.368656	-5.155568	-3.135010
1	-1.845512	-1.271303	-4.589295
1	-1.650025	1.137072	-4.047931
1	0.889749	0.137313	-0.778134
6	-0.030374	2.219517	-2.139331
6	-1.030242	3.202275	-2.219494
6	1.288766	2.629147	-1.887256
6	-0.721149	4.551105	-2.051422
1	-2.059970	2.900265	-2.391349
6	1.598515	3.977928	-1.725348
1	2.078942	1.884226	-1.844443
6	0.595378	4.944760	-1.806657
1	-1.511806	5.295055	-2.103994
1	2.626750	4.274424	-1.535950
1	0.836595	5.995790	-1.673382
6	-0.030374	2.219517	2.139331
6	-1.030242	3.202275	2.219494
6	1.288766	2.629147	1.887256
6	-0.721149	4.551105	2.051422
1	-2.059970	2.900265	2.391349
6	1.598515	3.977928	1.725348
1	2.078942	1.884226	1.844443
6	0.595378	4.944760	1.806657
1	-1.511806	5.295055	2.103994
1	2.626750	4.274424	1.535950
1	0.836595	5.995790	1.673382

[5,5]HPP, E_{el} (B3LYP-D3/6-31G(d)) = −2000.89906597 Hartree, Imaginary Frequencies = 0

6	-0.868075	-2.594164	0.188262	6	7.045363	1.222214	0.634217
6	-0.260829	-1.588315	0.956726	6	7.045367	-1.222212	-0.634215
6	-0.222487	-3.844164	0.130506	1	7.067289	-0.131934	-2.460799
6	0.996317	-1.782997	1.520431	6	7.059473	0.049334	1.378191
1	-0.730499	-0.611873	1.035703	1	7.017844	2.161607	1.176841
6	1.027650	-4.042835	0.706332	6	6.811757	-1.193575	0.758054
1	-0.700886	-4.666823	-0.393880	1	7.017853	-2.161605	-1.176839
6	1.699309	-2.993552	1.358051	1	7.067281	0.131936	2.460801
1	1.485007	-0.940516	1.999720	1	-1.297692	-3.473961	-2.311430
1	1.506170	-5.014386	0.615194	6	-2.130970	-2.883112	-1.943950
6	-0.868075	2.594164	-0.188264	6	-2.066673	-2.320427	-0.641652
6	-0.222487	3.844164	-0.130509	6	-3.234946	-2.683663	-2.736339
6	-0.260830	1.588314	-0.956726	1	-3.295640	-3.149275	-3.717244
6	1.027649	4.042834	-0.706335	6	-3.108781	-1.501572	-0.217992
1	-0.700887	4.666824	0.393877	1	-3.071495	-1.091038	0.783779
6	0.996317	1.782995	-1.520432	6	-4.328667	-1.907208	-2.287181
1	-0.730499	0.611871	-1.035702	6	-4.245511	-1.236123	-1.025712
6	1.699309	2.993551	-1.358054	6	-5.540314	-1.877167	-3.041204
1	1.506169	5.014386	-0.615198	1	-5.566787	-2.367969	-4.010813
1	1.485007	0.940514	-1.999720	6	-6.665851	-1.340597	-2.496839
6	3.155744	-3.020631	1.632915	6	-5.389516	-0.450943	-0.567382
6	4.033126	-3.658663	0.735677	6	-6.629683	-0.675075	-1.231344
6	3.746049	-2.174485	2.588271	1	-7.620365	-1.410807	-3.012546
6	5.358221	-3.253947	0.613761	6	-5.389515	0.450943	0.567384
1	3.642535	-4.373612	0.017125	6	-4.245510	1.236123	1.025713
6	5.064051	-1.757341	2.454508	6	-2.130970	2.883113	1.943949
1	3.135422	-1.743556	3.377030	6	-3.234944	2.683664	2.736339
6	5.856189	-2.165460	1.359165	6	-2.066673	2.320428	0.641652
1	5.953945	-3.688323	-0.183205	6	-4.328666	1.907208	2.287182
1	5.418289	-0.981826	3.125024	6	-7.853773	-0.279500	-0.622252
6	3.155744	3.020629	-1.632917	6	-3.108781	1.501572	0.217992
6	3.746049	2.174481	-2.588271	6	-6.629682	0.675075	1.231347
6	4.033126	3.658665	-0.735681	1	-1.297692	3.473963	2.311428
6	5.064051	1.757337	-2.454508	6	-5.540313	1.877168	3.041205
1	3.135422	1.743550	-3.377029	6	-7.853772	0.279499	0.622256
6	5.358221	3.253949	-0.613765	1	-3.295638	3.149277	3.717244
1	3.642535	4.373615	-0.017131	6	-6.665849	1.340597	2.496842
6	5.856189	2.165460	-1.359165	1	-8.786239	-0.494055	-1.138085
1	5.418288	0.981821	-3.125021	1	-5.566785	2.367970	4.010814
1	5.953945	3.688328	0.183200	1	-7.620363	1.410807	3.012549
6	6.811757	1.193577	-0.758053	1	-8.786238	0.494055	1.138090
6	7.059477	-0.049332	-1.378189	1	-3.071495	1.091037	-0.783778

Enantiomerization TS of **[5,5]HPP**, E_{el} (B3LYP-D3/6-31G(d)) = −2000.85483213 Hartree, Imaginary Frequencies = 1 (21.2 cm^{-1})

6	-7.358263	0.679763	2.160462	1	1.624247	2.434036	1.661388
6	-7.358268	-0.679775	2.160451	1	1.928618	3.700884	-2.435274
6	-6.491689	1.377309	1.282250	6	-0.819184	-2.636020	-0.709345
6	-6.491696	-1.377313	1.282231	6	-0.013604	-3.093398	-1.768461
6	-5.366563	0.733272	0.664280	6	-0.193453	-2.415102	0.528433
6	-5.366565	-0.733273	0.664271	6	1.344386	-3.334879	-1.595325
6	-6.920634	2.692447	0.909519	1	-0.458674	-3.259856	-2.745486
6	-6.322449	3.347096	-0.114599	6	1.167423	-2.648577	0.699666
6	-5.061545	2.876561	-0.588531	1	-0.787310	-2.084595	1.376244
6	-4.475966	1.672700	-0.061889	6	1.970058	-3.120269	-0.355104
1	-8.100974	1.240974	2.721403	1	1.928623	-3.700905	-2.435249
1	-8.100982	-1.240991	2.721383	1	1.624241	-2.434025	1.661401
1	-7.812290	3.091470	1.385654	6	3.437741	3.213431	-0.179033
1	-6.715834	4.284001	-0.499803	6	4.032057	3.523963	1.059741
6	-4.307749	3.725393	-1.426668	6	4.294629	2.774869	-1.201213
6	-3.066341	1.596804	-0.164426	6	5.352551	3.178931	1.332239
6	-2.293536	2.543207	-0.846631	1	3.428470	3.972283	1.844571
6	-2.952359	3.558627	-1.574817	6	5.608130	2.416914	-0.926298
1	-4.801705	4.588749	-1.865979	1	3.891829	2.555730	-2.185753
1	-2.540469	0.842181	0.396259	6	6.125132	2.478264	0.383756
1	-2.376134	4.284995	-2.138558	1	5.743906	3.345846	2.332495
6	-6.322457	-3.347085	-0.114641	1	6.170225	1.906428	-1.701115
6	-6.920646	-2.692444	0.909481	6	3.437740	-3.213435	-0.179010
6	-5.061548	-2.876551	-0.588558	6	4.294629	-2.774880	-1.201192
6	-4.475968	-1.672698	-0.061902	6	4.032055	-3.523956	1.059767
6	-4.307748	-3.725380	-1.426695	6	5.608130	-2.416924	-0.926280
6	-2.952356	-3.558619	-1.574830	1	3.891830	-2.555750	-2.185735
6	-2.293534	-2.543207	-0.846633	6	5.352549	-3.178922	1.332264
6	-3.066342	-1.596806	-0.164428	1	3.428467	-3.972269	1.844600
1	-6.715845	-4.283984	-0.499858	6	6.125131	-2.478264	0.383776
1	-7.812306	-3.091469	1.385606	1	6.170226	-1.906444	-1.701100
1	-4.801703	-4.588730	-1.866017	1	5.743902	-3.345828	2.332522
1	-2.376130	-4.284987	-2.138570	6	7.081731	1.403574	0.767701
1	-2.540472	-0.842187	0.396266	6	7.828665	0.695576	-0.199751
6	-0.819184	2.636016	-0.709352	6	6.779679	0.694590	1.946998
6	-0.193450	2.415106	0.528425	6	7.828665	-0.695581	-0.199746
6	-0.013608	3.093384	-1.768475	1	8.244286	1.219707	-1.056731
6	1.167426	2.648580	0.699652	6	6.779678	-0.694577	1.947003
1	-0.787306	2.084605	1.376240	1	6.288872	1.198909	2.773375
6	1.344383	3.334865	-1.595345	6	7.081731	-1.403571	0.767712
1	-0.458682	3.259837	-2.745499	1	8.244286	-1.219718	-1.056722
6	1.970059	3.120264	-0.355124	1	6.288872	-1.198889	2.773384

[5,6]HPP, E_{el} (B3LYP-D3/6-31G(d)) = −2231.97778112 Hartree, Imaginary Frequencies = 0

6	0.293130	2.574110	1.791960
6	-0.699130	1.779610	1.194100
6	0.589290	3.810790	1.189940
6	-1.287690	2.158960	-0.010950
1	-0.955280	0.818760	1.632810
6	-0.003330	4.191350	-0.007690
1	1.302870	4.477670	1.665880
6	-0.915370	3.347770	-0.664980
1	-1.977820	1.476630	-0.499190
1	0.262010	5.145440	-0.455210
6	-0.293130	-2.574110	1.791960
6	-0.589290	-3.810790	1.189940
6	0.699130	-1.779610	1.194100
6	0.003330	-4.191350	-0.007690
1	-1.302870	-4.477670	1.665880
6	1.287690	-2.158960	-0.010950
1	0.955280	-0.818760	1.632810
6	0.915370	-3.347770	-0.664980
1	-0.262010	-5.145440	-0.455210
1	1.977820	-1.476630	-0.499190
6	-1.300740	3.575400	-2.078600
6	-0.321900	4.014430	-2.986050
6	-2.537140	3.176290	-2.616710
6	-0.494890	3.871410	-4.356500
1	0.643880	4.343280	-2.613680
6	-2.717460	3.052120	-3.992020
1	-3.349530	2.906130	-1.946930
6	-1.659510	3.290150	-4.892870
1	0.351180	4.079600	-5.002960
1	-3.670700	2.684950	-4.362000
6	-1.569860	2.623720	-6.219340
6	-2.240760	1.401580	-6.407580
6	-0.538090	2.915180	-7.137360
6	-1.696880	0.405560	-7.209010
1	-3.081110	1.139050	-5.773060
6	0.015750	1.915190	-7.931520
1	-0.074660	3.897980	-7.137130
6	-0.453560	0.589560	-7.844240
1	-2.128420	-0.589160	-7.154850
1	0.908340	2.134850	-8.512220
6	1.300740	-3.575400	-2.078600
6	2.537140	-3.176290	-2.616710
6	0.321900	-4.014430	-2.986050
6	2.717460	-3.052120	-3.992020
1	3.349530	-2.906130	-1.946930
6	0.494890	-3.871410	-4.356500
1	-0.643880	-4.343280	-2.613680
6	1.659510	-3.290150	-4.892870
1	3.670700	-2.684950	-4.362000
1	-0.351180	-4.079600	-5.002960
6	1.569860	-2.623720	-6.219340
6	0.538090	-2.915180	-7.137360
6	2.240760	-1.401580	-6.407580
6	-0.015750	-1.915190	-7.931520
1	0.074660	-3.897980	-7.137130
6	1.696880	-0.405560	-7.209010
1	3.081110	-1.139050	-5.773060
6	0.453560	-0.589560	-7.844240
1	-0.908340	-2.134850	-8.512220
1	2.128420	0.589160	-7.154850
1	2.946510	2.864630	2.158750
6	2.471940	2.375060	3.003740
6	1.077330	2.108170	2.961760
6	3.215930	2.028160	4.105790
1	4.273890	2.275750	4.151520
6	0.501730	1.412860	4.018310
1	-0.566020	1.232660	3.998710
6	2.622580	1.382940	5.216850
6	1.248830	0.991880	5.148820
6	3.356890	1.213070	6.429790
1	4.407120	1.493060	6.450180
6	2.717460	0.813150	7.563270
6	0.645910	0.326280	6.299100
6	1.343140	0.416640	7.536560
1	3.240990	0.786920	8.515770
6	-0.645910	-0.326280	6.299100
6	-1.248830	-0.991880	5.148820
6	-2.471940	-2.375060	3.003740
6	-3.215930	-2.028160	4.105790
6	-1.077330	-2.108170	2.961760
6	-2.622580	-1.382940	5.216850
6	0.665580	0.151640	8.760430
6	-0.501730	-1.412860	4.018310
6	-1.343140	-0.416640	7.536560
1	-2.946510	-2.864630	2.158750
6	-3.356890	-1.213070	6.429790
6	-0.665580	-0.151640	8.760430
1	-4.273890	-2.275750	4.151520
6	-2.717460	-0.813150	7.563270
1	1.212630	0.260110	9.693530
1	-4.407120	-1.493060	6.450180
1	-3.240990	-0.786920	8.515770
1	-1.212630	-0.260110	9.693530
1	0.566020	-1.232660	3.998710

Enantiomerization TS of **[5,6]HPP**, E_{el} (B3LYP-D3/6-31G(d)) = −2231.92883544 Hartree, Imaginary Frequencies = 1 (22.0 cm^{-1})

6	-8.333578	0.680108	2.331669
6	-8.333578	-0.680110	2.331668
6	-7.482549	1.378923	1.438983
6	-7.482550	-1.378924	1.438981
6	-6.372062	0.732774	0.799315
6	-6.372062	-0.732774	0.799314
6	-7.911709	2.696363	1.073740
6	-7.335733	3.344479	0.032067
6	-6.093446	2.862752	-0.479364
6	-5.499426	1.662284	0.043772
1	-9.066343	1.240616	2.906300
1	-9.066343	-1.240619	2.906298
1	-8.788990	3.102449	1.570265
1	-7.734702	4.281899	-0.346221
6	-5.363599	3.688215	-1.361521
6	-4.094939	1.574598	-0.102177
6	-3.342270	2.490369	-0.842265
6	-4.018179	3.497043	-1.567254
1	-5.864733	4.549579	-1.796655
1	-3.553286	0.837386	0.465750
1	-3.455154	4.198583	-2.174613
6	-7.335733	-3.344479	0.032063
6	-7.911710	-2.696363	1.073736
6	-6.093446	-2.862751	-0.479367
6	-5.499426	-1.662284	0.043770
6	-5.363599	-3.688214	-1.361525
6	-4.018178	-3.497041	-1.567257
6	-3.342270	-2.490369	-0.842266
6	-4.094939	-1.574598	-0.102177
1	-7.734702	-4.281898	-0.346226
1	-8.788991	-3.102450	1.570261
1	-5.864733	-4.549577	-1.796660
1	-3.455153	-4.198581	-2.174616
1	-3.553287	-0.837387	0.465751
6	-1.862407	2.567135	-0.769434
6	-1.196754	2.449345	0.461674
6	-1.095397	2.923563	-1.892605
6	0.164539	2.717885	0.571657
1	-1.763527	2.189063	1.351577
6	0.264530	3.191257	-1.782775
1	-1.576438	3.003081	-2.863743
6	0.922858	3.114308	-0.543635
1	0.652609	2.611271	1.536395
1	0.820716	3.492290	-2.666404
6	-1.862407	-2.567135	-0.769434
6	-1.095396	-2.923562	-1.892605
6	-1.196754	-2.449346	0.461674
6	0.264531	-3.191256	-1.782775
1	-1.576437	-3.003079	-2.863744
6	0.164538	-2.717886	0.571657
1	-1.763528	-2.189065	1.351577
6	0.922858	-3.114308	-0.543634
1	0.820717	-3.492289	-2.666403
1	0.652608	-2.611272	1.536396
6	2.376206	3.369875	-0.415975
6	3.267643	2.917773	-1.401372
6	2.931872	3.973246	0.727514
6	4.640057	2.933612	-1.188875
1	2.879257	2.446209	-2.299684
6	4.308102	4.001198	0.935351
1	2.271759	4.401372	1.477198
6	5.194675	3.408659	0.016277
1	5.280807	2.449943	-1.918809
1	4.694240	4.439416	1.851897
6	2.376206	-3.369875	-0.415974
6	2.931871	-3.973246	0.727515
6	3.267644	-2.917773	-1.401371
6	4.308101	-4.001198	0.935353
1	2.271759	-4.401372	1.477199
6	4.640058	-2.933612	-1.188874
1	2.879257	-2.446208	-2.299683
6	5.194675	-3.408659	0.016279
1	4.694239	-4.439416	1.851899
1	5.280807	-2.449942	-1.918806
6	6.556159	2.947161	0.382628
6	6.800929	2.505406	1.696244
6	7.487553	2.531487	-0.589743
6	7.656181	1.437817	1.939533
1	6.178568	2.858811	2.512975
6	8.329055	1.451971	-0.349021
1	7.450601	2.953589	-1.590466
6	8.268869	0.746407	0.873052
1	7.670635	1.024640	2.942435
1	8.900917	1.057044	-1.182950
6	6.556159	-2.947161	0.382630
6	7.487553	-2.531488	-0.589740
6	6.800928	-2.505405	1.696246
6	8.329056	-1.451973	-0.349019
1	7.450602	-2.953592	-1.590463
6	7.656180	-1.437816	1.939534
1	6.178566	-2.858809	2.512977
6	8.268869	-0.746407	0.873053
1	8.900919	-1.057046	-1.182949
1	7.670633	-1.024637	2.942435

[5,7]HPP, E_{el} (B3LYP-D3/6-31G(d)) = −2463.05497658 Hartree, Imaginary Frequencies = 0

6	2.797430	2.140760	-1.307480
6	2.217810	1.802300	-0.072950
6	2.186200	3.159630	-2.059160
6	1.040410	2.410100	0.355140
1	2.656060	1.009850	0.527120
6	1.015140	3.771100	-1.626500
1	2.641530	3.481120	-2.991920
6	0.393940	3.384830	-0.426020
1	0.574990	2.068310	1.275300
1	0.571320	4.558170	-2.230010
6	2.592810	-2.073290	1.195870
6	1.768870	-2.890370	1.989020
6	2.242080	-1.928020	-0.158700
6	0.617100	-3.473420	1.476430
1	2.026100	-3.064620	3.029610
6	1.099570	-2.531690	-0.676080
1	2.845730	-1.299960	-0.808170
6	0.234920	-3.290130	0.135430
1	-0.022559	-4.038910	2.146790
1	0.864970	-2.387190	-1.726580
6	-0.962200	3.864600	-0.066910
6	-1.929020	4.033240	-1.072480
6	-1.384880	4.023380	1.265090
6	-3.270390	4.215190	-0.759950
1	-1.644960	3.911270	-2.113660
6	-2.728410	4.213650	1.578430
1	-0.654150	3.966040	2.067510
6	-3.714570	4.241840	0.574720
1	-3.997660	4.216680	-1.566000
1	-3.021480	4.295000	2.621800
6	-5.153590	4.002770	0.849110
6	-5.513240	3.116630	1.879070
6	-6.166100	4.362720	-0.062250
6	-6.722930	2.433050	1.845210
1	-4.776270	2.827350	2.622220
6	-7.377190	3.677530	-0.098130
1	-5.968040	5.125180	-0.811020
6	-7.623570	2.601719	0.776590
1	-6.881850	1.628000	2.556070
1	-8.093950	3.908929	-0.882070
6	-1.096799	-3.751920	-0.329560
6	-1.795920	-3.034480	-1.315790
6	-1.786119	-4.805290	0.303120
6	-3.146880	-3.261100	-1.551220
1	-1.311250	-2.210310	-1.829970
6	-3.144169	-5.019560	0.083640
1	-1.262919	-5.441480	1.011170
6	-3.871229	-4.203590	-0.799150
1	-3.679330	-2.607730	-2.236320
1	-3.659699	-5.797880	0.640760
6	-5.346899	-4.056700	-0.749850
6	-5.954909	-3.904750	0.507550
6	-6.116949	-3.714630	-1.876020
6	-7.139850	-3.190580	0.644400
1	-5.408789	-4.190770	1.402180
6	-7.317270	-3.024230	-1.738230
1	-5.723869	-3.906450	-2.871300
6	-7.774960	-2.609121	-0.470000
1	-7.467580	-2.938320	1.647460
1	-7.838150	-2.702851	-2.635650
6	-8.515660	-1.331171	-0.281550
6	-8.405830	-0.342331	-1.277670
6	-8.933790	-0.885621	0.990540
6	-8.373260	1.006519	-0.948030
1	-8.142050	-0.619451	-2.292760
6	-8.889130	0.464799	1.325250
1	-9.168390	-1.606581	1.768750
6	-8.443790	1.423199	0.393870
1	-8.082280	1.716289	-1.716420
1	-9.077670	0.760149	2.354410
1	3.232180	1.355850	-3.870890
6	4.060140	1.101220	-3.215870
6	3.979270	1.416030	-1.833530
6	5.181380	0.489270	-3.725890
1	5.258640	0.292310	-4.792650
6	5.021270	1.025100	-1.002280
1	4.976280	1.302790	0.042680
6	6.268200	0.131520	-2.891240
6	6.160010	0.329340	-1.479930
6	7.495680	-0.335600	-3.453070
1	7.551780	-0.499740	-4.526290
6	8.600180	-0.470790	-2.667360
6	7.273960	-0.058270	-0.620680
6	8.529020	-0.287610	-1.249840
1	9.562830	-0.724370	-3.104510
6	7.219150	-0.127160	0.822930
6	6.022050	-0.428360	1.597680
6	3.725960	-0.967140	3.152730
6	4.835950	-0.408990	3.741880
6	3.755290	-1.370010	1.789540
6	6.021090	-0.175030	3.003150
6	9.723910	-0.297250	-0.474850
6	4.895570	-1.087090	1.046950
6	8.432760	0.034700	1.546550
1	2.823010	-1.114240	3.736900
6	7.228180	0.223590	3.654130
6	9.681480	-0.042290	0.866560
1	4.823870	-0.157840	4.799930
6	8.401490	0.248920	2.961430
1	10.673460	-0.445920	-0.982820
1	7.206710	0.423360	4.722530
1	9.341210	0.446440	3.471340
1	10.597390	0.048190	1.445100
1	4.942140	-1.416630	0.016840

Enantiomerization TS of **[5,7]HPP**, E_{el} (B3LYP-D3/6-31G(d)) = −2463.00385560 Hartree, Imaginary Frequencies = 1 (20.3 cm^{-1})

6	-9.536690	0.681107	2.121739	1	-0.049108	-3.067717	-2.437319
6	-9.536660	-0.680106	2.122122	1	-0.537178	-3.020394	1.836168
6	-8.620022	1.380758	1.296732	6	1.329430	3.481197	-0.119423
6	-8.619992	-1.380178	1.297471	6	2.010296	4.239563	-1.090925
6	-7.466363	0.732113	0.744881	6	2.072392	3.045617	0.990890
6	-7.466366	-0.731804	0.745233	6	3.381010	4.468547	-1.005906
6	-9.015588	2.697563	0.891753	1	1.459102	4.652026	-1.931034
6	-8.368200	3.331563	-0.117193	6	3.437218	3.289200	1.086263
6	-7.099058	2.837598	-0.546347	1	1.593740	2.441608	1.755991
6	-6.543861	1.648746	0.039771	6	4.133169	3.965539	0.067826
1	-10.312346	1.240871	2.637924	1	3.879936	5.029552	-1.792135
1	-10.312290	-1.239612	2.638627	1	3.994774	2.863989	1.915796
1	-9.922770	3.112728	1.322829	6	1.329380	-3.481166	-0.119779
1	-8.738698	4.265006	-0.532598	6	2.010194	-4.239426	-1.091402
6	-6.315626	3.629823	-1.413638	6	2.072430	-3.045651	0.990502
6	-5.133508	1.554013	-0.012228	6	3.380922	-4.468386	-1.006511
6	-4.337883	2.433021	-0.748016	1	1.458952	-4.651840	-1.931503
6	-4.963732	3.414669	-1.548000	6	3.437263	-3.289227	1.085758
1	-6.784690	4.480504	-1.902308	1	1.593839	-2.441707	1.755691
1	-4.628544	0.844697	0.621703	6	4.133148	-3.965478	0.067219
1	-4.358363	4.079944	-2.156172	1	3.879802	-5.029304	-1.792832
6	-8.368286	-3.331661	-0.115529	1	3.994875	-2.864086	1.915289
6	-9.015569	-2.697194	0.893191	6	5.613618	3.884207	0.060102
6	-7.099218	-2.837863	-0.545094	6	6.378742	3.954677	1.238753
6	-6.543940	-1.648759	0.040439	6	6.260942	3.419721	-1.096117
6	-6.315934	-3.630420	-1.412214	6	7.635216	3.361873	1.312667
6	-4.964076	-3.415269	-1.546944	1	5.945588	4.400501	2.130584
6	-4.338099	-2.433302	-0.747453	6	7.505245	2.804175	-1.015725
6	-5.133593	-1.554043	-0.011826	1	5.715689	3.382226	-2.035184
1	-8.738816	-4.265306	-0.530450	6	8.157931	2.638054	0.221264
1	-9.922695	-3.112169	1.324566	1	8.158678	3.358949	2.264924
1	-6.785073	-4.481309	-1.900451	1	7.879111	2.289780	-1.895114
1	-4.358803	-4.080740	-2.154997	6	5.613599	-3.884164	0.059448
1	-4.628515	-0.844523	0.621787	6	6.260880	-3.419444	-1.096700
6	-2.863632	2.508026	-0.598932	6	6.378762	-3.954878	1.238059
6	-2.281768	2.588091	0.676295	6	7.505187	-2.803916	-1.016224
6	-2.024368	2.682009	-1.711108	1	5.715589	-3.381760	-2.035738
6	-0.933488	2.896022	0.832651	6	7.635242	-3.362095	1.312050
1	-2.912840	2.481735	1.554683	1	5.945632	-4.400876	2.129814
6	-0.672746	2.968317	-1.553984	6	8.157918	-2.638054	0.220776
1	-2.440601	2.590777	-2.711029	1	7.879023	-2.289337	-1.895518
6	-0.099560	3.117483	-0.277783	1	8.158740	-3.359366	2.264289
1	-0.537453	3.020597	1.836263	6	8.985124	1.415960	0.410478
1	-0.048598	3.067407	-2.437134	6	9.532664	0.696047	-0.672140
6	-2.863812	-2.508217	-0.598660	6	8.807938	0.693963	1.605735
6	-2.024755	-2.682321	-1.710970	6	9.532660	-0.695889	-0.672270
6	-2.281708	-2.588088	0.676468	1	9.826673	1.219327	-1.578271
6	-0.673087	-2.968542	-1.554060	6	8.807933	-0.694222	1.605606
1	-2.441188	-2.591260	-2.710823	1	8.450383	1.199052	2.496975
6	-0.933390	-2.895955	0.832610	6	8.985118	-1.415999	0.410214
1	-2.912620	-2.481627	1.554957	1	9.826664	-1.219003	-1.578499
6	-0.099652	-3.117521	-0.277948	1	8.450374	-1.199476	2.496750

[5,8]HPP, E_{el} (B3LYP-D3/6-31G(d)) = −2694.13193315 Hartree, Imaginary Frequencies = 0

6	10.722087	0.169175	0.662093
6	10.722163	-0.168083	-0.661657
6	9.498638	0.248551	1.386482
6	9.498769	-0.247899	-1.386092
6	8.262602	0.011800	0.723507
6	8.262637	-0.011482	-0.723186
6	9.518946	0.536674	2.788382
6	8.377851	0.509127	3.532358
6	7.156856	0.044853	2.954774
6	7.104894	-0.274627	1.563293
1	11.655937	0.322222	1.197142
1	11.656083	-0.320821	-1.196679
1	10.472212	0.788907	3.246367
1	8.395433	0.760621	4.589851
6	6.013525	-0.185947	3.758112
6	5.967055	-0.975668	1.094521
6	4.863787	-1.239216	1.897588
6	4.889153	-0.789686	3.245132
1	6.045604	0.108049	4.804627
1	5.970060	-1.351260	0.079639
1	4.016619	-0.933688	3.875040
6	8.378092	-0.509096	-3.531966
6	9.519188	-0.536186	-2.787957
6	7.156965	-0.045048	-2.954468
6	7.104891	0.274568	-1.563028
6	6.013659	0.185548	-3.757902
6	4.889212	0.789282	-3.245059
6	4.863723	1.238893	-1.897543
6	5.966947	0.975510	-1.094380
1	8.395766	-0.760732	-4.589421
1	10.472541	-0.788188	-3.245893
1	6.045844	-0.108508	-4.804398
1	4.016735	0.933271	-3.875055
1	5.969852	1.351257	-0.079547
6	3.678156	-1.957046	1.371027
6	3.249781	-1.791014	0.041856
6	2.912733	-2.795686	2.199667
6	2.085358	-2.397158	-0.417929
1	3.810460	-1.147332	-0.629466
6	1.744689	-3.394666	1.740752
1	3.232651	-2.976494	3.221956
6	1.287891	-3.189129	0.427671
1	1.781170	-2.238241	-1.448577
1	1.151836	-3.993065	2.426674
6	3.678092	1.956834	-1.371153
6	2.912981	2.795724	-2.199822
6	3.249386	1.790565	-0.042130
6	1.744906	3.394777	-1.741057
1	3.233152	2.976627	-3.222019
6	2.084902	2.396706	0.417486
1	3.809851	1.146692	0.629188
6	1.287753	3.188965	-0.428135
1	1.152303	3.993411	-2.426991
1	1.780419	2.237577	1.448010
6	-0.041089	-3.690154	0.003057
6	-0.594462	-4.884500	0.500394
6	-0.849372	-2.906103	-0.836951
6	-1.917618	-5.234795	0.239327

1	0.017840	-5.543983	1.109526
6	-2.167143	-3.256096	-1.099226
1	-0.469361	-1.963879	-1.220657
6	-2.748181	-4.407459	-0.535817
1	-2.316743	-6.156090	0.655878
1	-2.784487	-2.574221	-1.675822
6	-0.041266	3.689967	-0.003636
6	-0.594512	4.884488	-0.500700
6	-0.849688	2.905718	0.836063
6	-1.917645	5.234848	-0.239579
1	0.017878	5.544083	-1.109617
6	-2.167432	3.255768	1.098367
1	-0.469776	1.963345	1.219494
6	-2.748295	4.407397	0.535331
1	-2.316657	6.156303	-0.655883
1	-2.784905	2.573785	1.674703
6	-4.218737	-4.573802	-0.628484
6	-4.973912	-4.961103	0.493169
6	-4.930316	-4.115140	-1.750506
6	-6.340980	-4.705904	0.562842
1	-4.467369	-5.360449	1.367673
6	-6.290734	-3.843044	-1.674603
1	-4.394074	-3.880239	-2.665787
6	-7.007488	-4.031308	-0.477281
1	-6.873431	-4.941236	1.480419
1	-6.770643	-3.354998	-2.517322
6	-4.218832	4.573853	0.628231
6	-4.974145	4.961173	-0.493322
6	-4.930264	4.115216	1.750344
6	-6.341230	4.706011	-0.562820
1	-4.467698	5.360484	-1.367902
6	-6.290702	3.843164	1.674629
1	-4.393910	3.880274	2.665547
6	-7.007613	4.031430	0.477403
1	-6.873795	4.941341	-1.480326
1	-6.770498	3.355123	2.517415
6	-8.225750	3.213261	0.245472
6	-9.025978	2.721451	1.296136
6	-9.702834	1.510980	1.174509
6	-8.378452	2.590463	-1.006529
6	-9.054228	1.382943	-1.128538
6	-9.604795	0.743107	-0.002929
6	-9.702943	-1.510958	-1.173954
6	-9.604740	-0.743069	0.003461
6	-9.053921	-1.382857	1.128974
6	-8.378098	-2.590346	1.006855
6	-9.026042	-2.721393	-1.295685
6	-8.225608	-3.213159	-0.245160
1	-9.033583	3.235247	2.253901
1	-10.216905	1.104453	2.041764
1	-7.811015	2.948157	-1.859844
1	-8.981418	-0.846359	2.069896
1	-7.810482	-2.947999	1.860066
1	-9.033786	-3.235198	-2.253444
1	-10.217190	-1.104467	-2.041120
1	-8.981869	0.846463	-2.069482

Enantiomerization TS of **[5,8]HPP**, E_{el} (B3LYP-D3/6-31G(d)) = −2694.07911788 Hartree, Imaginary Frequencies = 1 (23.1 cm^{-1})

6	10.533924	-0.678124	2.206062	6	-2.257854	-4.568101	-1.076834
6	10.579721	0.680017	2.137771	1	-0.331834	-4.657814	-2.005692
6	9.609648	-1.389474	1.399048	6	-2.416682	-3.212317	0.900644
6	9.682041	1.369180	1.284556	1	-0.657133	-2.134677	1.462557
6	8.487324	-0.737024	0.787721	6	-3.041567	-4.063426	-0.027640
6	8.513151	0.728047	0.756373	1	-2.704224	-5.250278	-1.795870
6	9.975696	-2.737463	1.079949	1	-3.007934	-2.779479	1.702810
6	9.341354	-3.408244	0.087453	6	-0.312867	3.373574	-0.083642
6	8.112320	-2.894549	-0.423933	6	-0.878727	4.438642	-0.807820
6	7.573624	-1.655647	0.069308	6	-1.152013	2.664866	0.790248
1	11.283432	-1.235713	2.761589	6	-2.228004	4.760174	-0.689452
1	11.368624	1.239956	2.633275	1	-0.244843	5.034809	-1.458449
1	10.854387	-3.155386	1.564036	6	-2.500085	2.983044	0.905507
1	9.693708	-4.376060	-0.259486	1	-0.754961	1.817553	1.342679
6	7.347870	-3.706052	-1.291497	6	-3.075625	4.028726	0.158915
6	6.176458	-1.508379	-0.091880	1	-2.628103	5.596752	-1.256273
6	5.387669	-2.399248	-0.819448	1	-3.133866	2.367628	1.536798
6	6.015066	-3.453751	-1.518106	6	-4.508310	-4.261526	0.047785
1	7.812212	-4.598183	-1.704983	6	-5.172828	-4.453507	1.271739
1	5.660377	-0.732608	0.441217	6	-5.300866	-4.059499	-1.092761
1	5.419873	-4.134825	-2.118833	6	-6.549520	-4.282877	1.376286
6	9.442780	3.299475	-0.161743	1	-4.593165	-4.689204	2.160520
6	10.099325	2.667212	0.842303	6	-6.673365	-3.861959	-0.983267
6	8.151578	2.828270	-0.547587	1	-4.820572	-3.923714	-2.058028
6	7.592342	1.653880	0.062572	6	-7.317960	-3.875053	0.268326
6	7.353478	3.633476	-1.388037	1	-7.020308	-4.392086	2.349372
6	5.996553	3.435133	-1.486942	1	-7.222921	-3.554852	-1.867446
6	5.373731	2.456417	-0.679099	6	-4.544220	4.224274	0.197122
6	6.180381	1.582494	0.052503	6	-5.275956	4.504832	-0.970883
1	9.822453	4.218510	-0.600238	6	-5.279774	3.952321	1.363075
1	11.023424	3.070348	1.248134	6	-6.657562	4.338112	-1.013309
1	7.818816	4.479546	-1.888083	1	-4.747717	4.756661	-1.886429
1	5.391137	4.112534	-2.080471	6	-6.655972	3.768658	1.317216
1	5.690207	0.893648	0.721316	1	-4.757237	3.805327	2.304180
6	3.906767	-2.406699	-0.733088	6	-7.366500	3.861056	0.105010
6	3.275924	-2.344277	0.520000	1	-7.176760	4.489348	-1.955870
6	3.102047	-2.663290	-1.855016	1	-7.164809	3.426968	2.213219
6	1.917530	-2.612675	0.655041	6	-8.649766	3.123014	-0.015552
1	3.876680	-2.167900	1.408164	6	-9.468157	2.835580	1.095702
6	1.740031	-2.911251	-1.721229	6	-10.205452	1.656475	1.153330
1	3.554540	-2.676420	-2.843205	6	-8.862844	2.347939	-1.169662
6	1.121545	-2.936973	-0.457742	6	-9.597903	1.170549	-1.112529
1	1.485815	-2.637765	1.651222	6	-10.146359	0.718873	0.102416
1	1.146343	-3.089656	-2.612997	6	-10.206014	-1.691488	-0.705480
6	3.897029	2.498138	-0.548189	6	-10.129316	-0.748939	0.339488
6	3.089904	2.818195	-1.654266	6	-9.536061	-1.187945	1.537947
6	3.266541	2.368080	0.700202	6	-8.783675	-2.354419	1.576867
6	1.731813	3.071184	-1.506990	6	-9.453821	-2.862179	-0.664199
1	3.536269	2.871711	-2.643354	6	-8.597490	-3.135451	0.421987
6	1.904389	2.618328	0.847164	1	-9.435530	3.479865	1.970485
1	3.863440	2.141359	1.579570	1	-10.722916	1.402467	2.075010
6	1.114056	3.010839	-0.246359	1	-8.291972	2.551215	-2.070201
1	1.134799	3.311181	-2.382399	1	-9.483891	-0.516612	2.389234
1	1.459499	2.571912	1.837472	1	-8.185587	-2.544776	2.462145
6	-0.299769	-3.330754	-0.300941	1	-9.444649	-3.511186	-1.535857
6	-0.916194	-4.218859	-1.202424	1	-10.754115	-1.448789	-1.612381
6	-1.082713	-2.846610	0.761374	1	-9.561889	0.502432	-1.967261

References

(1) Mannancherry, R.; Šolomek, T.; Cavalli, D.; Malinčík, J.; Häussinger, D.; Prescimone, A.; Mayor, M. Sulfone "Geländer" Helices: Revealing Unexpected Parameters Controlling the Enantiomerization Process. *J. Org. Chem.* **2021**, *86* (8), 5431–5442. https://doi.org/10.1021/acs.joc.0c03016.

(2) Frisch, M. J.; Trucks, G. W.; Schlegel, H. B.; Scuseria, G. E.; Robb, M. A.; Cheeseman, J. R.; Scalmani, G.; Barone, V.; Mennucci, B.; Petersson, G. A.; Nakatsuji, H.; Caricato, M.; Li, X.; Hratchian, H. P.; Izmaylov, A. F.; Bloino, J.; Zheng, G.; Sonnenberg, J. L.; Hada, M.; Ehara, M.; Toyota, K.; Fukuda, R.; Hasegawa, J.; Ishida, M.; Nakajima, T.; Honda, Y.; Kitao, O.; Nakai, H.; Vreven, T.; Montgomery, J. A.; Peralta, J. E.; Ogliaro, F.; Bearpark, M.; Heyd, J. J.; Brothers, E.; Kudin, K. N.; Staroverov, V. N.; Kobayashi, R.; Normand, J.; Raghavachari, K.; Rendell, A.; Burant, J. C.; Iyengar, S. S.; Tomasi, J.; Cossi, M.; Rega, N.; Millam, J. M.; Klene, M.; Knox, J. E.; Cross, J. B.; Bakken, V.; Adamo, C.; Jaramillo, J.; Gomperts, R.; Stratmann, R. E.; Yazyev, O.; Austin, A. J.; Cammi, R.; Pomelli, C.; Ochterski, J. W.; Martin, R. L.; Morokuma, K.; Zakrzewski, V. G.; Voth, G. A.; Salvador, P.; Dannenberg, J. J.; Dapprich, S.; Daniels, A. D.; Farkas, Ö.; Foresman, J. B.; Ortiz, J. V.; Cioslowski, J.; Fox, D. J. *Gaussian 09 Revision D.01*; 2009.

(3) Frisch, M. J.; Trucks, G. W.; Schlegel, H. B.; Scuseria, G. E.; Robb, M. A.; Cheeseman, J. R.; Scalmani, G.; Barone, V.; Petersson, G. A.; Nakatsuji, H.; Li, X.; Caricato, M.; Marenich, A. V.; Bloino, J.; Janesko, B. G.; Gomperts, R.; Mennucci, B.; Hratchian, H. P.; Ortiz, J. V.; Izmaylov, A. F.; Sonnenberg, J. L.; Williams; Ding, F.; Lipparini, F.; Egidi, F.; Goings, J.; Peng, B.; Petrone, A.; Henderson, T.; Ranasinghe, D.; Zakrzewski, V. G.; Gao, J.; Rega, N.; Zheng, G.; Liang, W.; Hada, M.; Ehara, M.; Toyota, K.; Fukuda, R.; Hasegawa, J.; Ishida, M.; Nakajima, T.; Honda, Y.; Kitao, O.; Nakai, H.; Vreven, T.; Throssell, K.; Montgomery Jr., J. A.; Peralta, J. E.; Ogliaro, F.; Bearpark, M. J.; Heyd, J. J.; Brothers, E. N.; Kudin, K. N.; Staroverov, V. N.; Keith, T. A.; Kobayashi, R.; Normand, J.; Raghavachari, K.; Rendell, A. P.; Burant, J. C.; Iyengar, S. S.; Tomasi, J.; Cossi, M.; Millam, J. M.; Klene, M.; Adamo, C.; Cammi, R.; Ochterski, J. W.; Martin, R. L.; Morokuma, K.; Farkas, O.; Foresman, J. B.; Fox, D. J. Gaussian 16 Rev. C.02, 2016.

(4) Hanwell, M. D.; Curtis, D. E.; Lonie, D. C.; Vandermeersch, T.; Zurek, E.; Hutchison, G. R. Avogadro: An Advanced Semantic Chemical Editor, Visualization, and Analysis Platform. *J. Cheminformatics* **2012**, *4* (1), 17. https://doi.org/10.1186/1758-2946-4-17.

(5) Colwell, C. E.; Price, T. W.; Stauch, T.; Jasti, R. Strain Visualization for Strained Macrocycles. *Chem. Sci.* **2020**, *11* (15), 3923–3930. https://doi.org/10.1039/D0SC00629G.

(6) Ravat, P.; Hinkelmann, R.; Steinebrunner, D.; Prescimone, A.; Bodoky, I.; Juríček, M. Configurational Stability of [5]Helicenes. *Org. Lett.* **2017**, *19* (14), 3707–3710. https://doi.org/10.1021/acs.orglett.7b01461.

(7) Bruhn, T.; Schaumlöffel, A.; Hemberger, Y.; Bringmann, G. SpecDis: Quantifying the Comparison of Calculated and Experimental Electronic Circular Dichroism Spectra. *Chirality* **2013**, *25* (4), 243–249. https://doi.org/10.1002/chir.22138.
